# Directional immuno–metabolic signatures following severe trauma are associated with organ-specific clinical endpoints in a prospective cohort

**DOI:** 10.3389/fimmu.2026.1964036

**Published:** 2026-10-28

**Authors:** Marcin Osuchowski, Gerhard Fritsch, Gebhard Woisetschläger, Johannes Grillari, Franz-Josef Nierscher, Alexandra Pomberger, Hubert Hetz, Heinz Steltzer, Roman Ullrich, Carolina Zeller, Behnaz Jafarmadar, Susanne Kerbl, Soheyl Bahrami, Johannes Zipperle

**Affiliations:** 1 Ludwig Boltzmann Institute for Traumatology, the Research Center in Cooperation with AUVA, Vienna, Austria; 2 AUVA Trauma Center Salzburg, Department of Anesthesiology and Intensive Care Medicine, Academic Teaching Hospital of the Paracelsus Medical University, Salzburg, Austria; 3 Institute for Molecular Biotechnology, BOKU University, Vienna, Austria; 4 Department of Anesthesiology and Intensive Care Medicine, AUVA Trauma Center Vienna, Vienna, Austria

**Keywords:** trauma, acute kidney injury, ARDS, biomarker, ICU, immunosuppression, organ failure, sepsis

## Abstract

**Background:**

Severe trauma elicits evolving immune-metabolic responses that may differ across clinical endpoints. We characterized time-resolved innate and adaptive immune patterns associated with higher or lower endpoint odds during the first 96 h after injury.

**Methods:**

Of 62 patients prospectively enrolled in the observational PRIME study (ISS >15), 52 met the prespecified analytical completeness criterion. Clinical and immune biomarkers were assessed at emergency room admission (ER), 24, 48, and 96 h. Time-point-specific quartile deviations were evaluated using logistic regression against microbiological culture positivity during hospitalization, creatinine-defined renal impairment, Berlin-defined ARDS of any severity, hepatic enzyme elevation, and four hemodynamic endpoints. Complementary sensitivity analyses and bootstrap resampling assessed the exploratory associations and their internal stability.

**Results:**

Microbiological culture positivity was associated with lower admission base excess and temperature, reduced CD4/CD8 ratios and CD4^+^ T-cell proportions at 24–48 h, and lower monocyte measures with higher IL-8 at 96 h. The 96-h monocyte CBC/IL-8 combination showed an apparent within-cohort AUC of 0.78. Creatinine-defined renal impairment was associated with lower admission pH and base excess and a higher-IL-8/lower-CD4/CD8 combination (apparent AUC 0.88), alongside later myeloid and lymphocyte-related alterations. Berlin-defined ARDS showed predominantly low-odds associations, including higher early pH and phagocytic capacity; its 96-h combination of lower phagocyte CD64 expression and lower lymphocyte percentage yielded an apparent AUC of 0.80. Hepatic enzyme elevation showed low-odds associations involving higher 96-h monocyte HLA-DR expression and lower CD64^+^ phagocyte counts (apparent AUC 0.78). Hemodynamic endpoints showed distinct, endpoint-specific high-odds configurations. Bootstrap selection reproducibility was limited for the retained multivariable combinations.

**Conclusion:**

The evaluated clinical endpoints were associated with distinct directional immune-metabolic configurations rather than a uniform inflammatory response. Their hypothesis-generating contribution lies in the endpoint-specific organization of established biological components across serial sampling points. These exploratory candidate signatures require independent validation before their potential clinical relevance can be established.

## Introduction

Severe traumatic injury remains one of the leading causes of death and long-term morbidity in people under 65 years of age. Advances in prehospital care, rapid evacuation, damage-control resuscitation, and early in-hospital trauma management have substantially reduced immediate trauma-related mortality (1–3). However, patients who survive the initial phase of hemorrhage and tissue damage remain at risk for evolving complications, including secondary infections, organ dysfunction, and circulatory deterioration.

These complications arise from a multilayered post-traumatic disruption of cellular homeostasis, involving acute-phase proteins, cytokines, alarmins, and other humoral mediators that interact through auto-, para-, and endocrine signaling pathways (4–6). Cellular injury releases damage-associated molecular patterns (DAMPs), which can trigger systemic inflammatory response syndrome (SIRS) and initiate broad innate immune activation (7, 8). However, the magnitude and direction of this response are shaped by host factors such as age, comorbidities, medication, frailty, and genetic predisposition. Consequently, post-traumatic immune trajectories may range from sterile hyperinflammation to cellular anergy and immune paralysis (9, 10).

Recent trauma and critical illness studies have further shown that early immune activation and immunosuppression may coexist, with elevated IL-6, IL-8, IL-10, IL-1ra, and MCP-1, altered monocyte HLA-DR and CD64 expression, and lymphocyte depletion being associated with heterogeneous trajectories of infection, organ dysfunction, and shock (11–14). Similar time-dependent host-response concepts have also been emphasized in recent sepsis immune-profiling and biomarker frameworks, which argue for multiparametric, longitudinal “combitypes” rather than single-marker decision rules (15, 16).

Characterization of clinically relevant post-traumatic phenotypes in polytrauma populations is further complicated by heterogeneity in demographics, preexisting conditions, injury pattern, anatomical distribution, hemorrhagic burden, resuscitation requirements, and the dynamic evolution of physiology over time. Established scoring systems such as the Injury Severity Score (ISS), Abbreviated Injury Scale (AIS), Trauma and Injury Severity Score (TRISS), and International Classification of Diseases-based Injury Severity Score (ICISS) provide standardized descriptions of trauma severity, but they offer only a limited snapshot of evolving post-traumatic immune–metabolic interactions (17). Their comparative performance is also affected by methodological heterogeneity across cohorts and endpoints (18). Machine-learning and multi-omics approaches may improve phenotypic stratification, but they often require large datasets, external validation, and complex infrastructures that are not readily available in routine trauma settings (19–21). At the same time, mechanistic insight frequently arises from smaller but deeply phenotyped cohorts, where Dynamic Network Analysis, fuzzy clustering, multi-omics integration, and single-cell transcriptomics have revealed reproducible immune–metabolic trajectories from hyperacute cytokine release to later immunodysfunction and organ-specific complications (22–25). These studies illustrate that deeply phenotyped cohorts can reveal biologically plausible and hypothesis-generating patterns that complement findings from larger studies, but require analytic strategies that prioritize interpretability, robustness, and biological directionality.

The primary aim of this study was to characterize time-resolved immune–metabolic patterns across the first 96 h after severe polytrauma, conceptualized as an early post-traumatic immune–metabolic continuum, and to determine whether these patterns were associated with organ- and system-specific clinical endpoints. We integrated routine clinical, blood gas, and hematological parameters with non-routine innate and adaptive immune markers and circulating cytokines measured from emergency room admission through 96 h. Using a quartile-based directional approach, we specifically assessed whether lower- or upper-range biomarker deviations identified distinct time-resolved high- and low-odds signatures across microbiological culture positivity, creatinine-defined renal impairment, Berlin-defined ARDS of any severity, hepatic enzyme elevation, and hemodynamic endpoints. This directionality-focused framework was intended to capture biologically interpretable differences across serial sampling time points within the evolving post-traumatic continuum rather than to derive clinically validated cut-offs or prognostic models.

We hypothesized that the direction and timing of these biomarker deviations would reveal endpoint-specific immune–metabolic phenotypes with distinct temporal organization across the early post-traumatic continuum.

## Materials and methods

### Study design and patients

This prospective two-site observational study, termed “Polytrauma and Resuscitation Impact on Innate Immunity (PRIME), “ enrolled 62 patients between June 2017 and January 2022. For the present time-resolved analysis, patients were included if serial clinical and immunological data were available at all scheduled sampling time points (ER, 24 h, 48 h, and 96 h) through the end of the 96-h observation period or, for patients who died earlier, through the time of death. Fifty-two patients fulfilled this complete serial analytical data criterion and constituted the final analysis cohort. For the longitudinal analysis, dataset completeness was defined as availability of all scheduled study measurements up to the end of the 96-h observation period or, in patients who died earlier, up to the time of death. Measurements scheduled after death were considered structurally unavailable and did not constitute missing longitudinal data. Death during the observation period was therefore not itself a reason for exclusion. Clinical characterization included ISS, RTS, SAPS III, prehospital GCS, documented TBI status and diagnosis, and available information on operative treatment and timing relative to initial blood sampling. The study was approved by the ethics committee of the Austrian Workers’ Compensation Board (AUVA, Votum Nr. 9/2016, amendment No. 09/2019) and registered at ClinicalTrials.gov under the identifier NCT06314841. Participants admitted to the emergency rooms of either of the two AUVA Trauma Center Vienna sites (Meidling or Brigittenau) within 3 h after injury were considered for enrollment. The two study sites belonged to the same trauma center organization and followed shared clinical and laboratory SOPs, centralized quality assurance, and common data-management procedures. All samples were transported using the same system, and laboratory processing was standardized to a fixed time point 1 h after blood collection; samples arriving earlier were held until this predefined processing time. Eligible patients were at least 18 years old and hospitalized with an Injury Severity Score (ISS) >15, a commonly used threshold for defining severely injured trauma cohorts. Exclusion criteria included recent intake of anti-inflammatory medication (NSAIDs or corticosteroids) prior to injury, known arterial hypertension, valvular heart disease, atrial fibrillation, heart failure, coronary artery disease, peripheral arterial disease, chronic obstructive pulmonary disease, asthma, diabetes mellitus, rheumatoid arthritis, inflammatory bowel disease, malignant neoplasia, other autoimmune diseases, neurological diseases, or renal failure, and active pregnancy or breastfeeding. Pre-existing conditions beyond these prespecified exclusion criteria were not systematically recorded. Informed consent was obtained from participants or their healthcare proxy using institutionally approved methods. For patients who died before informed consent could be obtained, the requirement for consent was waived in accordance with the ethics approval. The study adhered to the principles outlined in the Declaration of Helsinki and Good Clinical Practice standards.

### Blood samples

Blood samples were collected through a peripheral venous line in the emergency room (ER) and through a central venous catheter upon admission to the intensive care unit (ICU). Samples were collected into 3.5-mL tubes containing either buffered 3.2% trisodium citrate, EDTA, or lithium heparin (Vacuette, Greiner Bio-One, Linz, Austria) at baseline (ER, 0 h) and 24, 48, and 96 h after admission. Fresh blood samples were transported using a standardized transport system within 1 h to the Ludwig Boltzmann Institute for Traumatology, a biomedical research facility in cooperation with AUVA, for characterization of patient immune function using flow cytometry and cytokine immunoassays. Blood samples were then prepared for flow cytometry, and the residual blood was centrifuged at 2, 500 x g for 15 min to obtain platelet-poor plasma. A minimum of 500 µL of plasma from each anticoagulant was aliquoted into cryotubes and stored at -80 °C for further analysis. Clinical chemistry, blood gas, and complete blood count analyses were performed at the enrolling sites, and the results were incorporated into the central study database to complement flow cytometry and cytokine data. Biomarker assays were performed under pseudonymized study identifiers and independently of clinical endpoint assessment, with re-identification of individual participants restricted to the study leadership after study completion.

### Flow cytometry

Anticoagulated blood samples containing either citrate, EDTA, or lithium heparin were transferred to round-bottom FACS tubes (Greiner Bio-One, Linz, Austria), depending on the specific flow cytometry panel. For leukocyte subset characterization, 2 × 50 µL of EDTA whole blood was stained with monocyte- or lymphocyte-targeted antibody combinations. To assess human leukocyte antigen-DR (HLA-DR) and integrin expression on monocytes and granulocytes, 50 µL of whole blood was stained with 3 µL of fluorophore-labeled antibodies against the monocyte/macrophage marker and LPS co-receptor CD14 (clone 61D3, PE-Cyanine7), HLA-DR (clone L243, PE), integrin alpha subunit CD11b (clone ICRF44, FITC), and Fc receptor CD64 (clone 10.1, PerCP-eFluor™ 710). Lymphocyte subsets were identified using conjugated antibodies against CD4 (clone RPA-T4, FITC), CD8a (clone OKT8, PE-Cyanine7), CD25 (clone BC96, PE-Cyanine5), and CD127 (clone eBioRDR5, PE). Platelet-leukocyte aggregates were assessed in citrate-anticoagulated blood using antibodies targeting CD11b, CD14, and platelet-specific P-selectin (CD62p, clone Psel.KO.2.12, PE). Phagocytic capacity was evaluated using heparinized blood with CD11b antibodies and a pH-sensitive E. coli-conjugated biosensor (pHrodo™ BioParticles™, Invitrogen™). After vortexing, samples were incubated with antibodies at room temperature for 30 min in darkness, with one tube placed on ice for comparison. Red cell lysis was performed using 1× multispecies RBC lysis buffer (Thermo Fisher, Waltham, MA, USA), followed by washing steps with PBS. Samples were resuspended in 300 µL PBS and kept on ice until analysis on a CytoFLEX flow cytometer (Beckman Coulter Life Sciences, Brea, CA, USA). Data were analyzed using CytExpert 1.1 software (Beckman Coulter, USA). Leukocytes were gated by morphology (FSC-SSC) and surface antigen expression. Granulocytes were defined as SSC^high CD11b^high-CD14^- and monocytes as SSC^low-CD11b^+ CD14^high. Expression levels of CD11b, HLA-DR, and CD64 were reported as mean or median fluorescence intensity in arbitrary units (a.u.), as specified for the respective marker. Lymphocyte subsets were further gated by lineage-specific markers, with regulatory T-cells defined as CD4^+CD25^+CD127^low. Phagocytic activity was determined by gating for CD11b^+ cells and calculating the percentage of E. coli^+ cells. Platelet-leukocyte aggregates (PLAs) were identified as SSC^high CD11b^high-CD14^-CD62p^+ for granulocyte-platelet aggregates and SSC^low-CD11b^+CD14^high-CD62p^+ for monocyte-platelet aggregates. Signal specificity was validated using isotype controls and fluorescence-minus-one (FMO) staining. All antibodies used for flow-cytometric phenotyping, including clone, fluorochrome conjugate, and manufacturer information, are also reported in the accompanying cytometry reporting checklist. A representative gating strategy, including singlet discrimination and representative controls for the monocyte, lymphocyte, phagocytosis, and platelet–leukocyte aggregate panels, is provided in **Supplementary Figure S1**.

### Cytokine immunoassays

Plasma samples were thawed at 4 °C, vortexed, and prepared for a commercial bead-based Luminex immunoassay (ProcartaPlex™, Invitrogen™, Waltham, MA, USA). The assay targeted interleukin-1 receptor antagonist (IL-1ra), interleukins IL-6, IL-8, IL-10, interferon gamma-induced protein 10 (IP-10), and monocyte chemoattractant protein-1 (MCP-1). Samples were incubated with color-coded beads pre-coated with analyte-specific capture antibodies, followed by the addition of biotinylated detection antibodies to form antibody-antigen complexes. Phycoerythrin (PE)-conjugated streptavidin was added to enable fluorescence-based detection. Analyte concentrations were calculated from median fluorescence intensity values using assay-specific standard curves and were reported in pg/mL.

### Statistical analysis

Clinical endpoints were analyzed as binary outcomes. Microbiological culture positivity was defined as any positive microbiological culture during the entire hospitalization. Hepatic enzyme elevation was defined as GPT/ALAT ≥35 U/L, creatinine-defined renal impairment as serum creatinine >1.2 mg/dL, and the pulmonary endpoint as Berlin-defined ARDS of any severity. The originally prespecified additional criterion of PaO_2_/FiO_2_ <200 mmHg did not identify any additional patients because all patients meeting this criterion also fulfilled Berlin ARDS criteria. Hemodynamic endpoints comprised tachycardia (HR ≥100/min), hypotension (MAP ≤60 mmHg), catecholamine requirement, and lactate increase relative to admission. For all endpoints except microbiological culture positivity, the observation period extended from ER admission through 96 h. Patients were classified as endpoint-positive if the respective endpoint occurred at any time during its predefined observation period and endpoint-negative otherwise.

Biomarkers measured at ER, 24 h, 48 h, and 96 h were categorized using time-point-specific 25th and 75th percentiles. Lower- and upper-quartile thresholds were calculated separately for each biomarker at each sampling time point; measurements were not pooled across time points. Two directional binary variables were thereby generated for each biomarker, representing lower- and upper-quartile deviations relative to the remainder of the respective time-point-specific distribution. Across the prespecified biomarker set, this resulted in 144 primary directional tests (72 biomarkers × 2 directions). Univariable binary logistic regression with a logit link was used to relate each directional biomarker variable to endpoint status. Candidate associations were retained when the respective coefficient had p <0.05, the likelihood-ratio test indicated improvement over the null model, and the apparent AUC was ≥0.65. AUCs are reported with bootstrap 95% confidence intervals (1, 000 resamples, BCa method) and are interpreted as measures of apparent within-cohort discrimination rather than validated predictive performance. Odds ratios with 95% confidence intervals and additional descriptive model characteristics are reported in **Supplementary Table S1**. The cohort-derived quartile thresholds used in the primary analysis are likewise provided in **Supplementary Table S1** and should not be interpreted as clinically validated cut-offs.

Additive multivariable logistic-regression models comprising two or three retained variables, as well as two-way interaction models, were evaluated within the predefined model-selection framework. Retained multivariable combinations were required to satisfy the prespecified statistical and model-fit criteria, including significance of included terms, sensitivity and specificity ≥0.50 and lower AIC than the corresponding univariable models. Event counts and events per variable (EPV) were considered during model selection, with an intended minimum EPV of seven. Two reported two-predictor combinations, those for creatinine-defined renal impairment and tachycardia, had a tabulated EPV of six. These combinations are therefore presented as exploratory associations requiring particularly cautious interpretation. The corresponding EPV values and model estimates are reported in **Supplementary Tables S1**, **S3**. Given the limited event numbers, all retained multivariable combinations are interpreted as exploratory candidate signatures rather than clinical prediction models. Sparse-data sensitivity was assessed by re-estimating retained multivariable models using Firth-penalized logistic regression (**Supplementary Table S3**).

Several complementary sensitivity analyses were performed. Biomarkers retained in the primary quartile-based analysis were re-evaluated as continuous variables, with logarithmic transformation applied where appropriate (**Supplementary Table S2**). The signs of the estimated continuous-model coefficients were compared with the high- and low-odds directions identified in the primary quartile-based analysis. To explore major baseline covariates without overparameterizing the limited event sets, separate bivariate models were fitted in which each retained biomarker was adjusted individually for ISS, age, or sex (**Supplementary Tables S4A–C**). These analyses were intended to assess whether individual biomarker associations were materially altered by these covariates and were not designed to establish incremental prognostic value beyond a clinical reference model. For the clinical endpoints assessed through 96 h, an exploratory first-occurrence sensitivity analysis included only biomarker measurements obtained before the first recorded occurrence of the respective endpoint; the quartile thresholds from the primary analysis were carried forward unchanged and were not re-optimized (**Supplementary Table S5**). Microbiological culture positivity was not subjected to this analysis because the exact timing of first culture positivity could not be reliably reconstructed. Incident event counts were insufficient for estimation of selected time-restricted models. For endpoints observed through 96 h, first occurrence could only be assessed within this predefined study window; events occurring after 96 h were not captured by these endpoint definitions. The first-occurrence analyses therefore do not establish longer-term prognostic associations.

Coefficient stability was assessed by non-parametric bootstrap resampling (1, 000 resamples), including BCa 95% confidence intervals and sign stability. Coefficients whose BCa confidence interval included zero were considered to show reduced bootstrap stability and are marked accordingly in the main tables. In addition, the complete model-selection procedure was repeated in 500 bootstrap samples to assess selection reproducibility and optimism. Bootstrap inclusion probabilities, apparent AUCs, mean optimism, optimism-corrected AUCs, and coefficient-stability measures are reported in **Supplementary Table S6**. These analyses were used as internal sensitivity assessments and do not constitute external validation.

No formal multiplicity correction was applied. For the 144 primary directional tests, the first Benjamini–Hochberg critical value at a false-discovery rate of 0.05 would be 0.000347. The discrete p-value structure of the quartile-based small-sample comparisons further complicates interpretation of formal multiplicity-adjusted significance. Accordingly, the analysis was retained as an exploratory screening procedure, and all p values are reported as unadjusted and interpreted descriptively rather than confirmatorily. This approach does not control the false-discovery rate, and chance findings cannot be excluded. Formal model calibration was not performed because of the limited number of endpoint events and because the retained combinations were not developed or interpreted as clinical risk-prediction models. All retained associations therefore require validation in independent cohorts. Analyses were performed in R version 4.5.1 (R Foundation for Statistical Computing, Vienna, Austria); data preprocessing was performed in Microsoft Excel (Microsoft Corporation, Redmond, WA, USA), and graphical output was generated using GraphPad Prism (GraphPad Software, Boston, MA, USA).

## Results

### Cohort characteristics and definition of clinical endpoints

Of the 62 patients enrolled in PRIME, 52 fulfilled the complete analytical serial-data criterion and constituted the final analysis cohort. The 10 patients without complete longitudinal analytical data showed no marked descriptive imbalance in the available baseline characteristics. Median age was 44.5 years (IQR 35.0–55.75) versus 50.5 years (IQR 32.0–58.8), median ISS was 26.5 (IQR 23.25–36.5) versus 25.5 (IQR 18.3–32.8), and 7/10 (70.0%) versus 38/52 (73.1%) were male in the non-included and included groups, respectively. Importantly, none of the 10 non-included patients died during hospitalization. Reasons for non-inclusion were transfer to another institution (n=2), police/forensic circumstances interfering with serial study sampling (n=1), retrospective correction of ISS below the prespecified inclusion threshold (n=1), missed scheduled blood draws (n=2), and technical or analytical measurement failure (n=4). Of the 52 patients included in the analysis, 49 were enrolled at the Meidling site and 3 at the Brigittenau site. Patients were longitudinally profiled at ER, 24 h, 48 h, and 96 h. Operative treatment was documented in 48/52 patients; sufficiently complete operative information was unavailable for the remaining four patients. Among the 48 patients with documented operative timing, 43 (89.6%) underwent their first procedure within 24 h and 42 (87.5%) within ±7 h of initial blood sampling. Median RTS was 7.84 (IQR, 5.36–7.84), median SAPS III was 45 (IQR, 35.0–63.8), and median prehospital GCS was 13 (IQR, 7–15); these additional severity measures were available in 50 patients. Traumatic brain injury (TBI) was documented in 27/52 patients (51.9%), encompassing concussion, cerebral contusion, intracranial hemorrhage, and cranial or skull-base fractures. Median length of stay was 11 days (IQR, 7–23), and five of 52 patients (9.6%) died during hospitalization. One patient died after the initial ER blood collection and clinical assessment but before the subsequent scheduled sampling time points. This patient contributed the available ER biomarker and clinical endpoint data and remained included because measurements scheduled after death were considered structurally unavailable rather than missing. The remaining four patients who died had completed all scheduled measurements through 96 h. Clinical endpoints were analyzed as binary outcomes and related to biomarker patterns across the predefined observation periods.

The evaluated organ-specific endpoints comprised microbiological culture positivity (any culture-positive finding during hospitalization; n = 16, 30.8%), creatinine-defined renal impairment (serum creatinine > 1.2 mg/dL; n = 14, 26.9%), Berlin-defined ARDS of any severity; (n = 28, 53.8%; 13 mild, 10 moderate, and 5 severe according to maximum recorded severity), and hepatic enzyme elevation (GPT/ALAT ≥ 35 U/L; n = 34, 65.4%). Hemodynamic endpoints included tachycardia (heart rate ≥ 100/min; n = 18, 34.6%), hypotension (mean arterial pressure ≤ 60 mmHg; n = 11, 21.2%), catecholamine requirement (n = 11, 21.2%) and lactate increase relative to admission (n = 15, 28.8%). The temporal distribution of first recorded endpoint occurrence is summarized in **Table 1**. Several endpoints were already present at admission, whereas others accumulated during the subsequent 96 h. The number of newly recorded first events declined substantially at later time points for most endpoints, limiting the feasibility of time-restricted landmark analyses beyond the early observation period. An inclusion flow diagram depicting the composition of the final analysis cohort is provided in **Figure 1**.

**Table 1:** Temporal distribution of first recorded clinical endpoint occurrence.

Clinical endpoint	Overall endpoint-positive, n/N (%)	First recorded at ER, n	First recorded at 24 h, n	First recorded at 48 h, n	First recorded at 96 h, n
Microbiological culture positivity	16/52 (30.8%)	—	—	—	—
Creatinine-defined renal impairment	14/52 (26.9%)	10	1	1	2
Berlin-defined ARDS of any severity	28/52 (53.8%)	16	3	7	2
Hepatic enzyme elevation	34/52 (65.4%)	30	3	1	0
Tachycardia (HR ≥100/min)	18/52 (34.6%)	15	0	0	3
Catecholamine requirement	11/52 (21.2%)	3	6	0	2
Hypotension (MAP ≤60 mmHg)	11/52 (21.2%)	3	7	0	1
Lactate increase relative to admission	15/52 (28.8%)	0	12	2	1

For endpoints assessed during the first 96 h, columns indicate the first study time point at which the respective endpoint was recorded as positive; each endpoint-positive patient is counted once at the first recorded occurrence. Overall endpoint-positive counts refer to the respective predefined observation period. Microbiological culture positivity was assessed over the entire hospitalization; because the exact timing of first culture positivity was not reliably represented by the study time-point structure, first-occurrence timing is not displayed for this endpoint. ER, emergency room admission; ARDS, acute respiratory distress syndrome; HR, heart rate; MAP, mean arterial pressure.

**Figure 1:**
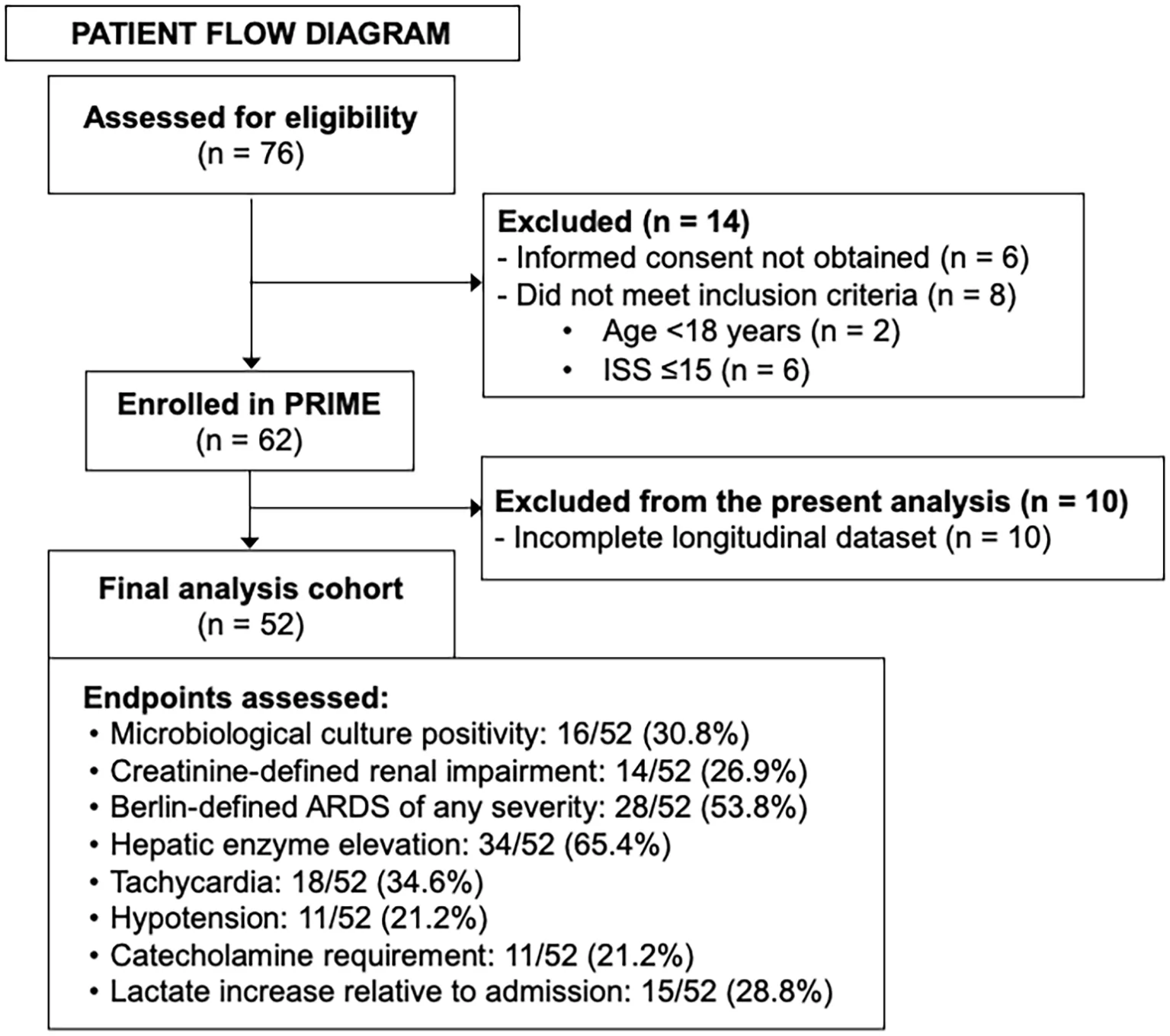
Flow diagram of patients assessed for eligibility, enrolled in PRIME, and included in the final analysis cohort. Exclusions at each stage are shown. Endpoint frequencies refer to the final analysis cohort and are not mutually exclusive. ISS, Injury Severity Score.

Detailed first-occurrence sensitivity analyses restricted to biomarker measurements preceding the respective endpoint are provided in **Supplementary Table S5**.

In bivariate sensitivity models considering each biomarker together with ISS, age, or sex separately, most biomarker associations retained their direction and nominal statistical evidence. Selected associations were attenuated, including IL-8 at 96 h for microbiological culture positivity and neutrophil CBC at 48 h for Berlin-defined ARDS after ISS adjustment, as well as selected renal and pulmonary markers after adjustment for ISS or age. Notably, for creatinine-defined renal impairment, both ISS and the admission CD4/CD8 ratio remained associated with the endpoint in the same model. Sex-adjusted models were additionally limited by the small number of women in the cohort (n=14). Continuous-biomarker sensitivity analyses showed that the estimated coefficient directions were consistent with the corresponding high- and low-odds patterns in all 48 evaluated associations. However, 14 of the 48 continuous models did not retain nominal statistical significance (p ≥0.05), and seven showed an apparent AUC below 0.65. Thus, directional consistency did not uniformly translate into comparable statistical evidence or discrimination when biomarkers were modeled continuously (**Supplementary Table S2**). Complete results of the ISS-, age-, and sex-adjusted sensitivity analyses are provided in **Supplementary Tables S4A–C**, respectively.

Bootstrap resampling of the complete model-selection procedure showed limited selection reproducibility of the displayed multivariable candidate patterns. For the displayed multivariable combinations, inclusion probabilities ranged from 0.16 to 0.54, whereas optimism-corrected AUCs ranged from 0.69 to 0.85. Selected individual biomarker associations showed higher inclusion probabilities, indicating that reproducibility differed between individual signals and their retained multivariable combinations. These findings indicate that apparent discrimination and selection reproducibility were not interchangeable and support interpretation of the retained models as exploratory candidate signatures rather than stable prognostic models (**Supplementary Table S6**).

### Time-resolved immune and physiologic signatures associated with microbiological culture-positivity

Among the 52 trauma patients included in the final analysis, 16 (30.8%) met the culture-positivity endpoint. Directionally interpretable associations with AUC ≥0.65 delineated a time-resolved pattern beginning with lower base excess and temperature at admission, followed by changes in adaptive immune markers and later reductions in monocyte-related measures (depicted in both CBC and flow cytometry) together with higher IL-8 (**Figure 2A**). At emergency room admission (ER), the microbiological culture-positivity endpoint was associated with lower base excess (↓base excess; AUC 0.68) and lower body temperature (↓temperature; AUC 0.67). Together, these findings characterized a physiologic high-odds profile associated with culture positivity at presentation.

**Figure 2:**
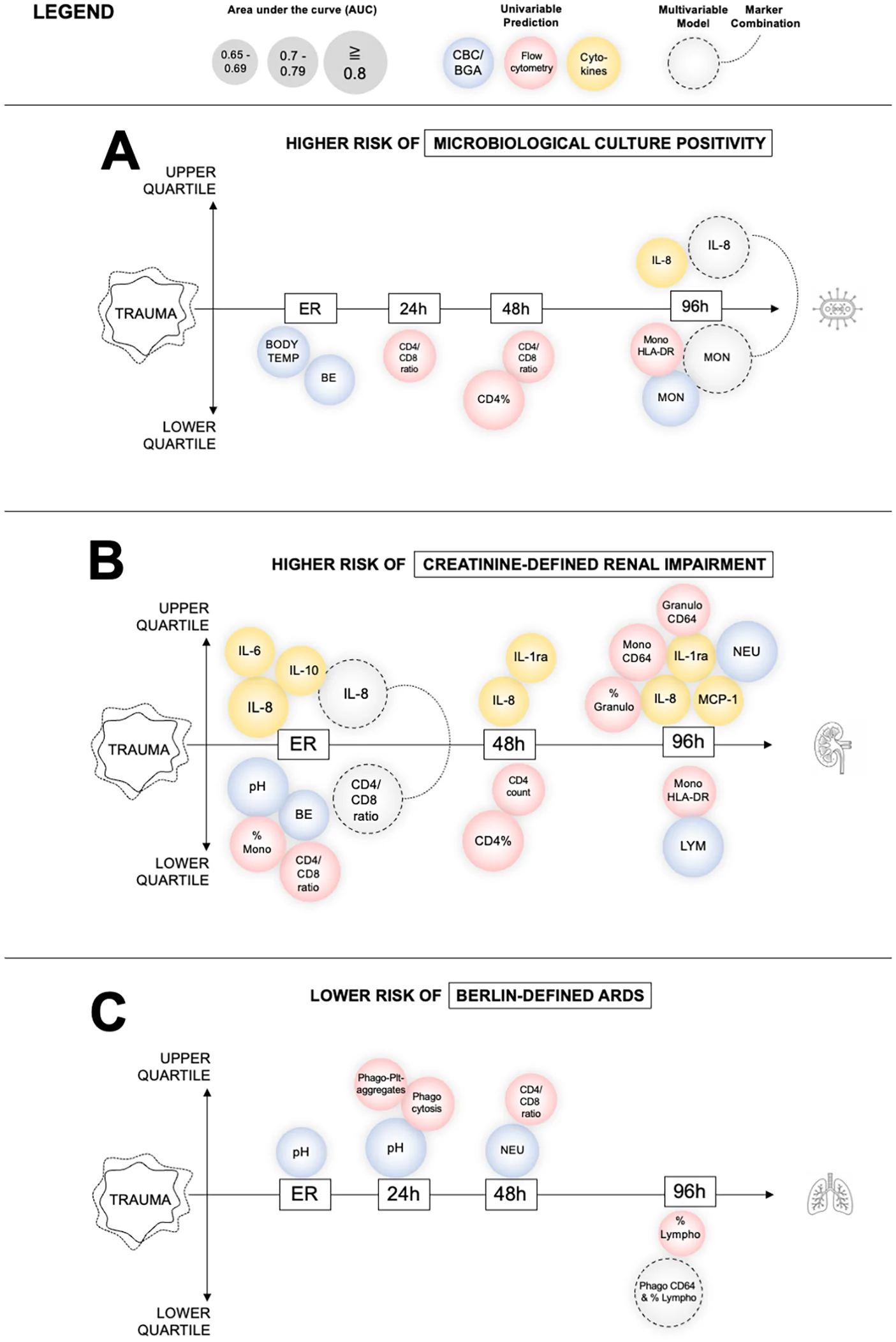
Time-resolved biomarker patterns associated with post-traumatic clinical endpoints. Temporal distribution of biomarker patterns associated with higher odds of microbiological culture positivity **(A)**, higher odds of creatinine-defined renal impairment **(B)**, and lower odds of Berlin-defined ARDS **(C)** after severe trauma. Biomarkers are positioned according to the sampling time point at which the respective association was observed: emergency room admission (ER), 24 h, 48 h, and 96 h after admission. The vertical axis indicates the direction of the association with markers above the timeline representing values in the upper quartile and markers below the timeline representing values in the lower quartile. Bubble size indicates apparent within-cohort discrimination according to the area under the receiver operating characteristic curve (AUC): small bubbles, AUC 0.65–0.69; medium bubbles, AUC 0.70–0.79; large bubbles, AUC ≥0.80. Corresponding AUCs with 95% confidence intervals are reported in the accompanying main-text tables; the AUCs shown here represent internally estimated, apparent within-cohort discrimination and should not be interpreted as externally validated predictive performance. Bubble color denotes biomarker domain: blue, routine clinical laboratory or blood gas analysis (CBC/BGA); red, flow-cytometric cellular immune markers; yellow, cytokines. Solid bubbles represent univariable associations. Dashed bubbles represent multivariable models or marker combinations; dotted connector lines indicate the univariable markers included in the corresponding multivariable model. For panels **(A, B)**, bubble locations indicate biomarker directions associated with higher odds of the respective endpoint. For panel **(C)** bubble locations indicate biomarker directions associated with lower odds of Berlin-defined ARDS. AUC, area under the curve; ARDS, acute respiratory distress syndrome; BE, base excess; BGA, blood gas analysis; CBC, complete blood count; ER, emergency room; HLA-DR, human leukocyte antigen-DR; IL, interleukin; LYM, lymphocyte count; MCP-1, monocyte chemoattractant protein-1; MON, monocyte count; NEU, neutrophil count.

Adaptive immune divergence became apparent during the early post-traumatic phase. A lower CD4/CD8 ratio was associated with higher odds of the culture-positivity endpoint at 24 h and 48 h (↓CD4/CD8; AUC 0.68 at both time points), and reduced CD4^+^ T-cell proportions at 48 h showed apparent discrimination of endpoint status (↓CD4^+^%; AUC 0.73).

By 96 h, the directional pattern shifted toward lower monocyte-related measures and higher IL-8. The combined late model of low monocyte CBC and high IL-8 showed the highest apparent discrimination among the 96-h culture-positivity patterns (↓monocyte CBC + ↑IL-8; AUC 0.78), forming a combined late high-odds signature characterized by monocyte reduction and IL-8 elevation. These findings are summarized in **Table 2**.

**Table 2:** Time-resolved directional biomarker patterns associated with microbiological culture positivity.

Category	Marker	ER	24 h	48 h	96 h	Description
Clinical / Physiologic	Base Excess	↓ 0.68 (0.55–0.82); <−2.70; OR 6.2 (1.6–26.0), p=0.0086				Lower base excess at admission characterized an early high-odds culture-positivity pattern.
	Body temperature	↓ 0.67 (0.53–0.80); <35.6; OR 5.0 (1.4–19.6), p=0.016				Lower admission temperature characterized an early high-odds culture-positivity pattern.
Innate immunity	Monocyte CBC				↓ 0.71 (0.58–0.84); <6.13; OR 11.0 (2.6–59.8), p=0.0022†	Lower monocyte CBC values at 96 h characterized a late high-odds culture-positivity pattern.
	Monocyte Count				↓ 0.68 (0.54–0.82); <26.0; OR 6.2 (1.6–26.0), p=0.0086†	Lower monocyte counts at 96 h paralleled the CBC-based monocyte signal within the late high-odds pattern.
	Monocyte Median HLA-DR expression				↓ 0.68 (0.53–0.82); <5186; OR 6.2 (1.6–26.0), p=0.0086†	Lower monocyte HLA-DR expression at 96 h was associated with higher odds of the culture-positivity endpoint.
Adaptive immunity	CD4/CD8 ratio		↓ 0.68 (0.54–0.83); <0.959; OR 6.2 (1.6–26.0), p=0.0086	↓ 0.68 (0.55–0.82); <1.20; OR 6.2 (1.6–26.0), p=0.0086†		Lower CD4/CD8 ratios at 24 and 48 h characterized a high-odds culture-positivity pattern.
	CD4^+^ %			↓ 0.73 (0.59–0.85); <2.79; OR 10.3 (2.6–48.1), p=0.0014		Lower CD4^+^ T-cell proportions at 48 h were associated with higher odds of the culture-positivity endpoint.
Cytokines	IL-8				↑ 0.68 (0.55–0.82); >4.79; OR 6.2 (1.6–26.0), p=0.0086†	Higher IL-8 at 96 h characterized the late high-odds culture-positivity pattern.
Multivariable model	Monocyte CBC ↓ + IL-8 ↑				0.78 (0.60–0.91); Monocyte CBC <6.13: OR 10.2 (2.1–65.4), p=0.0073 †; IL-8 >4.79: OR 6.3 (1.1–43.2), p=0.044 †	The combination of lower monocyte CBC and higher IL-8 defined the late multivariable high-odds culture-positivity signature.

↑, upper quartile or ↓, lower quartile of the respective parameter associated with higher odds of meeting the microbiological culture-positivity endpoint during hospitalization. Values indicate AUC (95% CI), the corresponding quartile-derived threshold, OR (95% CI), and p value at the respective sampling time point. ^†^placed after the p value indicates that the corresponding coefficient estimate showed reduced bootstrap stability, with the 95% BCa bootstrap confidence interval including zero; in multivariable models, ^†^refers only to the individually marked coefficient. AUCs represent apparent within-cohort discrimination and should not be interpreted as prospective predictive performance. Only directionally interpretable quartile-based associations with AUC ≥0.65 are shown. Multivariable models are displayed separately when all included variables retained clear upper/lower quartile directionality. “High-odds” denotes an association with higher odds of the endpoint and does not imply temporal prediction or causality.

### Time-resolved immune and physiologic signatures associated with creatinine-defined renal impairment

Among the 52 trauma patients included in the final analysis, 14 patients met the creatinine-defined renal impairment endpoint (serum creatinine >1.2 mg/dL) and were characterized by time-resolved associations involving physiologic, innate immune, adaptive immune, and cytokine markers (**Figure 2B**). At emergency room admission (ER), the endpoint was associated with lower pH (↓pH; AUC 0.71) and lower base excess (↓base excess; AUC 0.67). At the same time, reduced monocyte proportions (↓% monocytes; AUC 0.67) and lower CD4/CD8 ratios (↓CD4/CD8; AUC 0.72) were observed. Among cytokines, IL-8 showed the strongest admission signal (↑IL-8; AUC 0.72), while IL-10 and IL-6 were also elevated (both AUC 0.67). A combined admission signature comprising elevated IL-8 and a reduced CD4/CD8 ratio showed an apparent within-cohort AUC of 0.88 (↑IL-8 + ↓CD4/CD8; AUC 0.88).

At 48 h, the retained associations more prominently involved adaptive and inflammatory markers. Creatinine-defined renal impairment was associated with reduced CD4^+^ percentages (↓CD4^+^%; AUC 0.72) and lower CD4^+^ cell counts (↓CD4^+^ cell count; AUC 0.67). Higher IL-8 (↑IL-8; AUC 0.67) and IL-1ra (↑IL-1ra; AUC 0.67) accompanied this 48-h pattern.

At 96 h, the retained associations were predominantly myeloid and leukocyte-related and included higher IL-8, IL-1ra, and MCP-1. Increased monocyte median CD64 expression (↑monocyte CD64; AUC 0.72), neutrophil proportions (↑neutrophil CBC; AUC 0.70), granulocyte percentages (↑% granulocytes; AUC 0.67), and granulocyte median CD64 expression (↑granulocyte CD64; AUC 0.67) were associated with the creatinine-defined renal endpoint. In parallel, monocyte median HLA-DR expression and lymphocyte CBC were lower (↓monocyte HLA-DR expression; AUC 0.67; ↓lymphocyte CBC; AUC 0.70).

Overall, creatinine-defined renal impairment was characterized by an admission high-odds pattern comprising lower pH, base excess, monocyte proportion, and CD4/CD8 ratio together with higher IL-6, IL-8, and IL-10, with additional 48-h associations involving lower CD4^+^ T-cell proportions and counts and a 96-h profile characterized predominantly by higher myeloid CD64 expression and neutrophil measures alongside a lower lymphocyte fraction and monocyte HLA-DR expression. These findings are summarized in **Table 3**, with detailed results of the primary quartile-based analysis provided in **Supplementary Table S1**.

**Table 3:** Time-resolved directional biomarker patterns associated with creatinine-defined renal impairment.

Category	Marker	ER	24 h	48 h	96 h	Description
Clinical / Physiologic	pH	↓ 0.71 ( 0.55–0.85); <7.27; OR 7.1 (1.9–30.0), p=0.0051				Lower admission pH characterized an early high-odds renal pattern.
	Base Excess	↓ 0.67 (0.53–0.83); <−2.70; OR 5.3 (1.4–22.0), p=0.016				Lower base excess at admission characterized an early high-odds renal pattern
Innate immunity	% Monocytes	↓ 0.67 (0.50–0.81); <2.34; OR 5.3 (1.4–22.0), p=0.016				Reduced monocyte proportions at admission were associated with higher odds of creatinine-defined renal impairment.
	Monocyte Median CD64 expression				↑ 0.72 (0.56–0.85); >19,923; OR 8.8 (2.2–39.4), p=0.0026	Higher monocyte CD64 expression at 96 h was associated with higher odds of creatinine-defined renal impairment.
	Neutrophil CBC				↑ 0.70 (0.56–0.83); >78.48; OR 8.5 (2.0–40.9), p=0.0044	Higher neutrophil CBC at 96 h was associated with higher odds of creatinine-defined renal impairment.
	% Granulocytes				↑ 0.67 (0.53–0.82); >81.9; OR 5.3 (1.4–22.0), p=0.016	Higher granulocyte proportions at 96 h characterized the late high-odds renal pattern.
	Monocyte Median HLA-DR expression				↓ 0.67 (0.52–0.81); <5,186; OR 5.3 (1.4–22.0), p=0.016 †	Reduced late monocyte HLA-DR expression was associated with higher odds of creatinine-defined renal impairment..
	Granulocyte Median CD64 expression				↑ 0.67 (0.51–0.81); >1,590; OR 5.3 (1.4–22.0), p=0.016 †	Higher late granulocyte CD64 expression characterized the late high-odds renal pattern.
Adaptive immunity	CD4/CD8 ratio	↓ 0.72 (0.56–0.85); <0.768; OR 8.8 (2.2–39.4), p=0.0026				Lower CD4/CD8 ratio at admission characterized an early high-odds renal pattern.
	CD4^+^ %			↓ 0.72 (0.57–0.84); <2.79; OR 8.8 (2.2–39.4), p=0.0026		Reduced CD4^+^ T-cell proportions at 48 h were associated with higher odds of creatinine-defined renal impairment.
	CD4^+^ cell count			↓ 0.67 (0.52–0.81); <22.8; OR 5.3 (1.4–22.0), p=0.016 †		Lower CD4^+^ cell counts at 48 h were associated with higher odds of creatinine-defined renal impairment.
	Lymphocyte CBC				↓ 0.70 (0.56–0.85); <11.0; OR 8.5 (2.0–40.9), p=0.0044	Late lymphopenia characterized the late high-odds renal pattern.
Cytokines	IL-8	↑ 0.72 (0.57–0.86); >6.69; OR 8.8 (2.2–39.4), p=0.0026		↑ 0.67 (0.52–0.83); >2.82; OR 5.3 (1.4–22.0), p=0.016	↑ 0.67 (0.53–0.82); >4.79; OR 5.3 (1.4–22.0), p=0.016	Higher IL-8 was associated with higher odds of creatinine-defined renal impairment at ER, 48 h, and 96 h.
	IL-10	↑ 0.67 (0.54–0.81); >8.54; OR 5.3 (1.4–22.0), p=0.016 †				Higher IL-10 at admission was associated with higher odds of creatinine-defined renal impairment.
	IL-6	↑ 0.67 (0.51–0.82); >46.7; OR 5.3 (1.4–22.0), p=0.016 †				Higher admission IL-6 was associated with higher odds of creatinine-defined renal impairment.
	IL-1ra			↑ 0.67 (0.49–0.80); >166; OR 5.3 (1.4–22.0), p=0.016	↑ 0.67 (0.52–0.81); >361; OR 5.3 (1.4–22.0), p=0.016	Elevated IL-1ra at 48 and 96 h characterized the intermediate-to-late high-odds renal pattern.
	MCP-1				↑ 0.67 (0.51–0.82); >37.6; OR 5.3 (1.4–22.0), p=0.016 †	Late MCP-1 elevation was associated with higher odds of creatinine-defined renal impairment.
Multivariable model	IL-8 ↑ + CD4/CD8 ratio ↓	**0.88** (0.72–0.95); IL-8 >6.69: OR 26.7 (3.7–557.3), p=0.0050; CD4/CD8 ratio <0.77: OR 33.6 (4.6–715.9), p=0.0030				The combination of higher IL-8 and lower CD4/CD8 ratio defined the admission multivariable high-odds renal signature and showed the highest apparent discrimination among the renal signatures.

↑, upper or ↓, lower range quartile-based associations, respectively, with higher odds of meeting the creatinine-defined renal impairment endpoint during the predefined observation period. Values indicate AUC (95% CI), the corresponding quartile-derived threshold, OR (95% CI), and p value at the respective sampling time point. ^†^placed after the p value indicates that the corresponding coefficient estimate showed reduced bootstrap stability, with the 95% BCa bootstrap confidence interval including zero; in multivariable models, ^†^refers only to the individually marked coefficient. AUCs represent apparent within-cohort discrimination and should not be interpreted as prospective predictive performance. Only directionally interpretable quartile-based associations with AUC ≥0.65 are shown. Multivariable models are displayed separately when all included variables retained clear upper/lower directionality. “High-odds” denotes an association with higher odds of the endpoint and does not imply temporal prediction or causality.

AUC values ≥0.80 are shown in bold.

### Time-resolved immune and physiologic signatures associated with lower risk of berlin-defined ARDS

Among the 52 trauma patients included in the final analysis, 28 (53.8%) met the Berlin-defined ARDS endpoint during the observation period. According to maximum recorded severity, 13 patients had mild, 10 moderate, and 5 severe ARDS. A PaO_2_/FiO_2_ ratio <200 mmHg was observed in 15 patients, all of whom also fulfilled Berlin ARDS criteria; thus, the additional P/F-ratio criterion of the original endpoint definition did not alter patient classification. In contrast to microbiological culture positivity and creatinine-defined renal impairment, the refined lung analysis predominantly identified directional associations with lower odds of Berlin-defined ARDS (**Figure 2C**).

Within the first 24 h, higher pH values were associated with lower odds of ARDS (↑pH; AUC 0.68 at ER; AUC 0.72 at 24 h). At 24 h, phagocytosis-related markers, i.e., phagocytic capacity (↑phagocytic capacity) and phagocyte mean platelet signal (↑phagocyte mean platelet signal), were likewise associated with lower odds of the endpoint and showed apparent discrimination of endpoint status (both AUC 0.66). At 48 h, a higher neutrophil proportion in the CBC (↑ neutrophil CBC; AUC 0.66), and a higher CD4/CD8 ratio were associated with lower odds of ARDS (↑CD4/CD8; AUC 0.68). At 96 h, lower lymphocyte percentage showed apparent discrimination of endpoint status in the low-odds direction (↓% lymphocytes; AUC 0.69). The 96-h multivariable model combining lower phagocyte median CD64 expression with lower lymphocyte percentage showed the highest apparent within-cohort discrimination among the pulmonary low-odds signatures (AUC 0.80).

Overall, lower odds of Berlin-defined ARDS were associated with higher early pH, higher phagocytic capacity and phagocyte-platelet interaction signal at 24 h, a higher CD4/CD8 ratio and neutrophil CBC at 48 h, and a late low-odds pattern combining lower phagocyte CD64 expression with lower lymphocyte percentage. These findings are summarized in **Table 4**, with detailed results of the primary quartile-based analysis provided in **Supplementary Table S1**.

**Table 4:** Time-resolved immune and physiologic biomarker patterns associated with lower odds of berlin-defined ARDS.

Category	Marker	ER	24 h	48 h	96 h	Description
Clinical / Physiologic	pH	↑ 0.68 (0.52–0.84); >7.37; OR 0.17 (0.03–0.76), p=0.023	↑ 0.72 (0.55–0.87); >7.44; OR 0.12 (0.02–0.54), p=0.0075			Higher pH at admission and 24 h was associated with lower odds of Berlin-defined ARDS.
Innate immunity	Phagocytic capacity		↑ 0.66 (0.48–0.81); >51.7; OR 0.22 (0.05–0.95), p=0.045 †			Higher phagocytic capacity at 24 h was associated with lower odds of Berlin-defined ARDS.
	Neutrophil CBC			↑ 0.66 (0.49–0.81); >82.2; OR 0.22 (0.05–0.95), p=0.045 †		Higher neutrophil CBC at 48 h was associated with lower odds of Berlin-defined ARDS.
Adaptive immunity	CD4/CD8 ratio			↑ 0.68 (0.51–0.83); >2.07; OR 0.17 (0.03–0.76), p=0.023		Higher CD4/CD8 ratio at 48 h was associated with lower odds of Berlin-defined ARDS.
	% Lymphocytes				↓ 0.69 (0.50–0.84); <11.6; OR 0.19 (0.04–0.81), p=0.028	Lower lymphocyte percentage at 96 h was associated with lower odds of Berlin-defined ARDS.
Thromboinflammation	Phagocyte Mean Platelet signal		↑ 0.66 (0.50–0.82); >528; OR 0.22 (0.05–0.95), p=0.045 †			Higher platelet-associated phagocyte signal at 24 h was associated with lower odds of Berlin-defined ARDS.
Multivariable model	Phagocyte Median CD64 ↓ + % Lymphocytes ↓				**0.80** (0.60–0.91); Phagocyte Median CD64 expression <13,596: OR 0.083 (0.008–0.561), p=0.017 †; % Lymphocytes <11.64: OR 0.087 (0.010–0.475), p=0.0092 †	The combination of lower phagocyte CD64 expression and lower lymphocyte percentage defined the 96-h multivariable low-odds ARDS signature.

↑ and ↓ indicate upper- and lower-range quartile-based associations, respectively, with lower odds of meeting the Berlin-defined ARDS endpoint during the observation period. Values indicate AUC (95% CI), the corresponding quartile-derived threshold, OR (95% CI), and p value at the respective sampling time point. ^†^placed after the p value indicates that the corresponding coefficient estimate showed reduced bootstrap stability, with the 95% BCa bootstrap confidence interval including zero; in multivariable models, ^†^refers only to the individually marked coefficient. AUCs represent apparent within-cohort discrimination and should not be interpreted as prospective predictive performance. Only directionally interpretable quartile-based associations with AUC ≥0.65 are shown. Multivariable models are displayed separately when all included variables retained clear upper/lower quartile directionality. “Low-odds” denotes an association with lower odds of the endpoint and does not imply protection, temporal prediction, or causality.

AUC values ≥0.80 are shown in bold.

### Time-resolved immune signatures associated with lower odds of hepatic enzyme elevation

Among the 52 trauma patients included in the final analysis, 34 (65.4%) met the hepatic enzyme elevation endpoint, defined as GPT/ALAT ≥35 U/L. As for Berlin-defined ARDS, the refined hepatic endpoint analysis predominantly identified directional associations with lower odds of hepatic enzyme elevation (**Figure 3A**).

**Figure 3:**
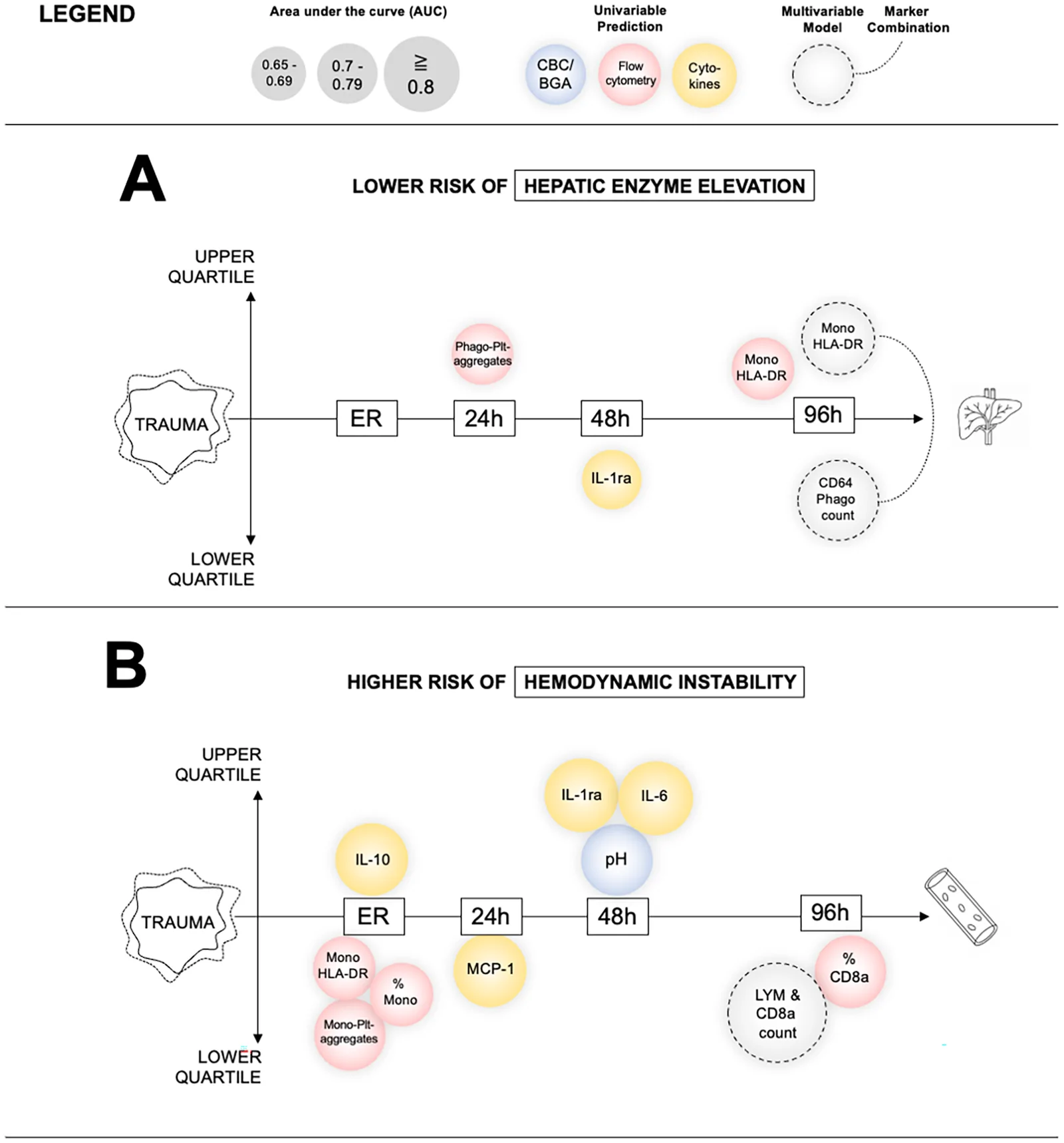
Time-resolved biomarker patterns associated with hepatic enzyme elevation and hemodynamic endpoints. Temporal distribution of biomarker patterns associated lower odds of hepatic enzyme elevation **(A)** and higher odds of hemodynamic endpoints **(B)** after severe trauma. Biomarkers are positioned according to the sampling time point at which the respective association was observed: emergency room admission (ER), 24 h, 48 h, and 96 h after admission. The vertical axis indicates the direction of the association with markers above the timeline representing values in the upper quartile and markers below the timeline representing values in the lower quartile. Bubble size indicates apparent within-cohort discrimination according to the area under the receiver operating characteristic curve (AUC): small bubbles, AUC 0.65–0.69; medium bubbles, AUC 0.70–0.79; large bubbles, AUC ≥0.80. Corresponding AUCs with 95% confidence intervals are reported in the accompanying main-text tables; the AUCs shown here represent internally estimated, apparent within-cohort discrimination and should not be interpreted as externally validated predictive performance. Bubble color denotes biomarker domain: blue, routine clinical laboratory or blood gas analysis (CBC/BGA); red, flow-cytometric cellular immune markers; yellow, cytokines. Solid bubbles represent univariable associations. Dashed bubbles represent multivariable models or marker combinations; dotted connector lines indicate the univariable markers included in the corresponding multivariable model. In panel **(A)**, biomarker patterns are shown in the direction associated with lower odds of hepatic enzyme elevation; in panel **(B)**, patterns are shown in the direction associated with higher odds of the respective hemodynamic endpoint. Hepatic enzyme elevation was defined as GPT/ALAT ≥35 U/L. Hemodynamic endpoints comprised tachycardia (HR ≥100/min), catecholamine requirement, hypotension (MAP ≤60 mmHg), and lactate increase relative to admission. ALAT, alanine aminotransferase; AUC, area under the curve; BGA, blood gas analysis; CBC, complete blood count; CD64, cluster of differentiation 64; CD8a, cluster of differentiation 8 alpha; ER, emergency room; GPT, glutamate pyruvate transaminase; HLA-DR, human leukocyte antigen-DR; IL, interleukin; LYM, lymphocyte count; MCP-1, monocyte chemoattractant protein-1; Mono, monocyte; Phago, phagocyte.

At 24 h, higher phagocyte mean platelet signal was associated with lower odds of hepatic enzyme elevation (↑phagocyte mean platelet signal; AUC 0.69). At 48 h, lower IL-1ra concentration was associated with lower odds of the endpoint (↓IL-1ra; AUC 0.68) and a similar association was observed at 96 h for higher monocyte median HLA-DR expression (↑monocyte median HLA-DR expression; AUC 0.69). The corresponding 96-h multivariable model combining higher monocyte HLA-DR expression with lower CD64^+^ phagocyte counts showed the highest apparent within-cohort discrimination among the hepatic low-odds signatures (↑monocyte HLA-DR expression and ↓CD64^+^ phagocyte count; AUC 0.78).

Overall, lower odds of hepatic enzyme elevation were associated with higher monocyte HLA-DR expression at 96 h, lower IL-1ra at 48 h, and higher phagocyte mean platelet signal at 24 h. Across individual and combined signatures, AUCs did not exceed 0.78. These findings are summarized in **Table 5**, with detailed results of the primary quartile-based analysis provided in **Supplementary Table S1**.

**Table 5:** Time-resolved directional biomarker patterns associated with lower odds of hepatic enzyme elevation.

Category	Marker	ER	24 h	48 h	96 h	Description
Innate immunity	Monocyte Median HLA-DR expression				↑ 0.69 ( 0.56–0.82); >10,855; OR 0.13 (0.030–0.51), p=0.0046	Higher monocyte HLA-DR expression at 96 h was associated with lower odds of hepatic enzyme elevation.
Cytokines	IL-1ra			↓ 0.68 (0.54–0.81); <59.1; OR 0.17 (0.043–0.62), p=0.0093 †		Lower IL-1ra at 48 h was associated with lower odds of hepatic enzyme elevation.
Thromboinflammation	Phagocyte Mean Platelet signal		↑ 0.69 (0.55–0.81); >528; OR 0.13 (0.030–0.51), p=0.0046			Higher platelet-associated phagocyte signal at 24 h was associated with lower odds of hepatic enzyme elevation.
Multivariable model	Monocyte Median HLA-DR ↑ + CD64^+^ Phagocyte count ↓				0.78 (0.63–0.88); Monocyte Median HLA-DR expression >10,855: OR 0.11 (0.020–0.52), p=0.0074; CD64^+^ Phagocyte count <24.1: OR 0.11 (0.020–0.52), p=0.0074	The combination of higher monocyte HLA-DR expression and lower CD64^+^ phagocyte counts defined the 96-h multivariable low-odds hepatic signature.

↑ and ↓ indicate upper- and lower-range quartile-based associations, respectively, with lower odds of meeting the hepatic enzyme elevation endpoint, defined as GPT/ALAT ≥35 U/L. Values indicate AUC (95% CI), the corresponding quartile-derived threshold, OR (95% CI), and p value at the respective sampling time point. ^†^placed after the p value indicates that the corresponding coefficient estimate showed reduced bootstrap stability, with the 95% BCa bootstrap confidence interval including zero; in multivariable models, ^†^refers only to the individually marked coefficient. AUCs represent apparent within-cohort discrimination and should not be interpreted as prospective predictive performance. Only directionally interpretable quartile-based associations with AUC ≥0.65 are shown. Multivariable models are displayed separately when all included variables retained clear upper/lower quartile directionality. “Low-odds” denotes an association with lower odds of the endpoint and does not imply protection, temporal prediction, or causality.

### Time-resolved immune and physiologic signatures associated with higher odds of hemodynamic endpoints

Four individual hemodynamic endpoints were evaluated: tachycardia (HR ≥100/min), catecholamine requirement, hypotension (MAP ≤60 mmHg), and lactate increase relative to admission. For all four endpoints, the retained associations were oriented toward higher odds of the respective endpoint (**Figure 3B**). These associations were therefore interpreted as endpoint-specific high-odds patterns rather than as markers of protection or circulatory stability.

For lactate increase relative to admission, higher pH at 48 h was associated with higher odds of the endpoint (↑pH; AUC 0.79). In addition, lower MCP-1 concentration at 24 h was associated with higher odds of this endpoint (↓MCP-1; AUC 0.71).

For tachycardia, several immune-associated markers were retained. At admission, higher IL-10 concentration showed the highest apparent discrimination among individual early tachycardia-associated markers (↑IL-10; AUC 0.78), whereas lower monocyte HLA-DR expression and lower monocyte proportions were also associated with higher odds of tachycardia (↓mean monocyte HLA-DR expression and ↓% monocytes; both AUC 0.69).

At 48 h, higher IL-1ra concentration was associated with higher odds of tachycardia (↑IL-1ra; AUC 0.73) whereas lower CD8a^+^ percentages were associated with higher odds at 96 h (↓CD8a^+^%; AUC 0.71). The 96-h multivariable model combining lower lymphocyte CBC with lower CD8a^+^ cell count showed the highest apparent within-cohort discrimination among the tachycardia-associated signatures (AUC 0.80).

For catecholamine requirement, higher IL-6 concentration at 48 h was associated with higher odds of the endpoint (↑IL-6; AUC 0.75). For hypotension/low MAP, lower P-selectin^+^ monocyte counts at admission were associated with higher odds of MAP ≤60 mmHg (↓P-selectin^+^ monocytes; AUC 0.75).

Overall, the four hemodynamic endpoints were associated with distinct time-resolved patterns involving metabolic, cytokine, cellular, and platelet-associated markers rather than a single shared immune signature. These findings are summarized in **Table 6**, with detailed results of the primary quartile-based analysis provided in **Supplementary Table S1**.

**Table 6:** Time-resolved directional biomarker patterns associated with higher odds of hemodynamic endpoints.

Category	Complication / endpoint	Marker	ER	24 h	48 h	96 h	Description
Clinical / Physiologic	Lactate increase relative to admission	pH			↑ 0.79 (CI 0.62–0.91); >7.46; OR 21.3 (4.8–125.0), p=0.00017		Higher pH at 48 h was associated with higher odds of lactate increase.
Innate immunity	Tachycardia	Mean Monocyte HLA-DR expression	↓ 0.69 (0.56–0.83); <15,798; OR 7.5 (2.0–33.5), p=0.0046				Lower admission monocyte HLA-DR expression was associated with higher odds of tachycardia..
	Tachycardia	% Monocytes	↓ 0.69 (0.56–0.82); <2.34; OR 7.5 (2.0–33.5), p=0.0046				Lower monocyte proportion at admission was associated with higher odds of tachycardia..
Adaptive immunity	Tachycardia	CD8a^+^ %				↓ 0.71 (0.58–0.83); <2.67; OR 10.3 (2.5–54.7), p=0.0023	Lower CD8a^+^ percentage at 96 h was associated with higher odds of tachycardia..
Cytokines	Tachycardia	IL-10	↑ 0.78 (0.65–0.88); >8.54; OR 25.1 (5.3–188.9), p=0.00023				Higher IL-10 at admission was associated with higher odds of tachycardia..
	Tachycardia	IL-1ra			↑ 0.73 (0.61–0.85); >166; OR 12.9 (3.2–68.8), p=0.00087		Higher IL-1ra at 48 h was associated with higher odds of tachycardia..
	Catecholamine requirement	IL-6			↑ 0.75 (0.58–0.90); >19.2; OR 10.2 (2.4–50.8), p=0.0024		Higher IL-6 at 48 h was associated with higher odds of catecholamine requirement.
	Lactate increase relative to admission	MCP-1		↓ 0.71 (0.58–0.84); <23.3; OR 8.9 (2.2–42.0), p=0.0033			Lower MCP-1 at 24 h was associated with higher odds of lactate increase.
Thromboinflammation	Hypotension / low MAP	P-selectin^+^ Monocytes count	↓ 0.75 (0.54–0.87); <8.97; OR 10.2 (2.4–50.8), p=0.0024				Lower P-selectin^+^ monocyte counts at admission were associated with higher odds of hypotension.
Multivariable model	Tachycardia	Lymphocyte CBC ↓ + CD8a^+^ cell count ↓				**0.80 (**0.61–0.92); Lymphocyte CBC <11.0: OR 10.1 (1.8–73.1), p=0.012; CD8a^+^ cell count <19.2: OR 7.3 (1.4–48.1), p=0.025 †	The combination of lower lymphocyte CBC and lower CD8a^+^ cell count defined the late multivariable high-odds tachycardia signature.

↑ and ↓ indicate upper- and lower-range quartile-based associations, respectively, with higher odds of the corresponding hemodynamic endpoint. Hemodynamic outcomes were evaluated separately as tachycardia (HR ≥100/min), catecholamine requirement, hypotension (MAP ≤60 mmHg), and lactate increase relative to admission. Values indicate AUC (95% CI), the corresponding quartile-derived threshold, OR (95% CI), and p value at the respective sampling time point. AUCs represent apparent within-cohort discrimination and should not be interpreted as prospective predictive performance. Only directionally interpretable quartile-based associations with AUC ≥0.65 are shown. Multivariable models are displayed separately when all included variables retained clear upper/lower quartile directionality. “High-odds” denotes an association with higher odds of the respective endpoint and does not imply temporal prediction or causality. ^†^placed after the p value indicates that the corresponding coefficient estimate showed reduced bootstrap stability, with the 95% BCa bootstrap confidence interval including zero; in multivariable models, ^†^refers only to the individually marked coefficient.

AUC values ≥0.80 are shown in bold.

## Discussion

In this prospective observational cohort of severely injured trauma patients, the evaluated clinical endpoints were not associated with a single uniform inflammatory phenotype. The analyzed endpoints showed two predominant directional patterns: microbiological culture positivity, creatinine-defined renal impairment, and the hemodynamic endpoints were predominantly characterized by high-odds signatures, whereas Berlin-defined ARDS and hepatic enzyme elevation were predominantly characterized by low-odds signatures. By focusing on cohort-derived quartile deviations across serial early sampling points rather than clinically optimized cut-offs, we identified interpretable directional patterns within the evolving early post-traumatic immune–metabolic continuum.

Within this small, deeply phenotyped cohort, this approach allowed biologically coherent patterns to be examined while limiting the complexity of higher-dimensional modeling. Similar concepts have emerged from larger trauma and critical illness studies, in which early immune and metabolic trajectories separated patients with divergent outcomes (22–24, 26, 27). Critically, our approach was not intended to derive clinical prediction rules, but to examine whether serial host-response profiles contained coherent high- and low-odds patterns across different clinical endpoints. This directional separation should be interpreted in the context of endpoint prevalence and quartile-based sampling, as frequent endpoints may preferentially reveal low-odds quartiles, while less frequent endpoints may more readily reveal high-odds quartiles. Accordingly, these directional classes should not be equated with demonstrated biological risk or protection. Rather, they provide a hypothesis-generating framework for identifying candidate host-response phenotypes that can be evaluated in larger independent cohorts.

The serial immune–metabolic pattern associated with microbiological culture positivity during hospitalization recapitulated several recognizable elements of post-traumatic immune dysregulation. Lower base excess and temperature at admission were consistent with an early physiologic and metabolic stress phenotype; associations at 24–48 h included lower CD4/CD8 ratios and reduced CD4-related measures, whereas the 96-h pattern included lower monocyte-related measures together with higher IL-8. Lymphocyte depletion, reduced CD4-related measures, and loss of monocyte HLA-DR expression are established hallmarks of post-traumatic immunosuppression and have repeatedly been associated with secondary infectious complications (28, 29). At the same time, the combination of higher IL-8 at 96 h with lower monocyte measures and reduced monocyte HLA-DR expression was compatible with concurrent inflammatory activity and adaptive/monocytic suppression, a mixed state now recognized as central in trauma and sepsis pathophysiology (11, 30, 31). Thus, the individual components of this signature are not novel in isolation. The hypothesis-generating observation in the present cohort is their serial organization across physiologic, adaptive, and myeloid/inflammatory domains in patients with microbiological culture positivity during hospitalization. This convergence with findings from larger trauma and critical-illness cohorts raises the hypothesis that such serial immune configurations may reflect a host-response state associated with susceptibility to microbiological complications. Because our endpoint was microbiological culture positivity rather than clinically adjudicated infection, this interpretation should ultimately be tested against prospectively adjudicated secondary infectious endpoints.

Creatinine-defined renal impairment showed a different but equally coherent pattern, in which familiar trauma physiology converged with immune phenotyping. Lower pH and base excess at admission, classical features of early post-traumatic metabolic stress and shock physiology, co-occurred with inflammatory cytokine signals, lower monocyte proportions, and a lower CD4/CD8 ratio. At 48 h, the retained pattern included lower CD4-related measures, whereas the 96-h profile was more strongly characterized by myeloid and leukocyte-associated markers, including higher CD64 and neutrophil measures together with lower lymphocyte and monocyte HLA-DR parameters. Acidosis, inflammatory activation, neutrophil responses, and lymphocyte depletion are established components of severe injury and have also been associated with acute kidney injury and organ dysfunction in critical illness (20, 32, 33). The pattern across serial sampling points raises the broader hypothesis that post-traumatic renal vulnerability may reflect the convergence of metabolic stress, inflammatory activation, and evolving cellular immune alterations. Although the pragmatic creatinine-defined endpoint should not be equated with KDIGO-defined AKI, the observed pattern provides a candidate serial immune–metabolic configuration for testing with conventional renal endpoints in larger cohorts.

In contrast to microbiological culture positivity, creatinine-defined renal impairment, and the later discussed hemodynamic patterns, the Berlin-defined ARDS and hepatic enzyme elevation endpoints yielded predominantly low-odds signatures. In the pulmonary endpoint, higher early pH, higher phagocytic capacity, higher CD4/CD8 ratios, and a 96-h combination of lower phagocyte CD64 expression and lower lymphocyte percentage, distinguished patients with lower odds of Berlin-defined ARDS. Dysregulated neutrophil activation together with altered innate and adaptive immune responses are well-established components of trauma-associated acute lung injury and ARDS (34–36). Against this background, the combination of higher early phagocytic activity with lower phagocyte CD64 expression at 96 h is particularly interesting. Rather than demonstrating biological protection, this pattern raises the hypothesis that pulmonary outcome may relate to the balance between cellular immune function and inflammatory activation across the post-traumatic continuum. Whether this low-odds configuration reflects a form of biological resilience or partly the statistical complement of a frequent endpoint cannot be determined from the present cohort and warrants prospective testing.

Hepatic enzyme elevation was similarly characterized predominantly by low-odds signatures. Lower odds of the endpoint were associated with higher phagocyte mean platelet signal at 24 h, lower IL-1ra at 48 h, and, at 96 h, higher monocyte HLA-DR expression together with lower CD64^+^ phagocyte counts. The liver is a central immune-metabolic organ after trauma, and sustained inflammatory burden has been linked to late hepatic dysfunction and multiple organ failure (8, 37). However, GPT/ALAT elevation is a biochemical marker rather than a measure of hepatic functional impairment, and post-traumatic aminotransferase levels may be influenced by direct tissue injury, hypoperfusion, and systemic inflammatory stress. Together, the higher HLA-DR/lower CD64-associated expression pattern raises the hypothesis that hepatic enzyme elevation may occur within broader systemic myeloid and immune–metabolic states. This interpretation remains exploratory, particularly because detailed region-specific hepatic injury information was not available.

Hemodynamic endpoints yielded predominantly high-odds signatures that were overlapping but not identical across tachycardia, hypotension, catecholamine requirement, and lactate increase. Rather than converging on a single “shock signature, “ the individual endpoints mapped to different combinations of cytokine, adaptive immune, platelet–monocyte, and metabolic markers. These markers span established inflammatory, counter-regulatory, and immunosuppressive components of the post-traumatic host response. In our cohort, tachycardia was associated with a mixed phenotype involving higher IL-10 and IL-1ra together with lower monocyte HLA-DR expression, monocyte proportions, and lymphocyte-related measures, whereas higher IL-6 at 48 h was associated with higher odds of catecholamine requirement. Hypotension and lactate increase, in turn, were associated with different platelet–monocyte and metabolic/cytokine signals. Catecholamine requirement should not be equated with hemorrhage or transfusion requirement, as it reflects a broader clinical need for hemodynamic support that may arise from several overlapping processes after severe trauma. Accordingly, the association with higher IL-6 at 48 h is interpreted here as an endpoint-associated inflammatory signal rather than evidence of a specific causal pathway linking IL-6 to vasopressor requirement. Treatment effects, resuscitation status, cardiovascular physiology, and evolving organ dysfunction may all contribute to this relationship.

The hypothesis-generating finding is therefore not that inflammatory mediators accompany post-traumatic circulatory stress, which is well established, but that different manifestations of hemodynamic instability were associated with distinct immune–metabolic configurations within the same early post-traumatic period. This suggests that tachycardia, vasopressor requirement, hypotension, and lactate dynamics may capture partly different dimensions of the host response rather than interchangeable expressions of a single shock phenotype. The association of lactate increase with higher pH at 48 h illustrates this complexity and may reflect time-dependent effects of resuscitation, metabolic recovery, or adrenergic stimulation rather than persistence of admission shock alone (38, 39).

A key methodological implication from our study is that small, deeply phenotyped trauma cohorts can benefit from direction-based serial analysis, especially when its purpose is biological pattern recognition and hypothesis generation rather than clinical prediction. The temporal distribution of the endpoints showed that some complications were already present early, whereas others accumulated during the subsequent 96 h. For endpoints with reconstructable event timing, first-occurrence sensitivity analyses restricted biomarker measurements to those preceding the respective first recorded endpoint occurrence and showed that the number of incident events declined rapidly at later sampling points, making comprehensive landmark modeling statistically uninformative in this cohort. The primary analysis should therefore be understood as integrating four serial snapshots within an evolving early post-traumatic continuum rather than treating ER, 24 h, 48 h, and 96 h as independent prognostic windows. Accordingly, individual biomarkers may reflect antecedent host states, concomitant responses, or the biological and treatment-related evolution of a complication rather than uniformly prospective signals.

Several components identified in the present study, metabolic stress, lymphocyte depletion, altered CD4/CD8 balance, monocyte HLA-DR suppression, cytokine activation, and myeloid remodeling, are established features of the response to severe trauma and should not themselves be considered novel. The contribution of the present dataset lies instead in showing how these familiar components assemble differently across clinical endpoints and serial sampling points within the same deeply phenotyped cohort. Related peri-operative data have likewise associated routinely measured immune-cell numbers and ratios with organ-specific adverse outcomes in transfused surgical patients, further supporting the relevance of cellular immune composition across major physiological insults (40). Experimental and clinical observations likewise underline that post-traumatic immune states cannot always be reduced to simple linear “more inflammation = worse outcome” relationships. For example, splenectomy in murine polytrauma improved survival after secondary sepsis despite mixed immunological effects (41), while in a prospective clinical cohort, serial immune profiles were inconsistent with injury severity score-based grouping and did not predict functional outcome linearly (14). These findings support the use of directionality and temporal context when interpreting complex post-traumatic immune phenotypes. Accordingly, the present signatures are best viewed as biologically informed candidate features for subsequent testing rather than as finished clinical classifiers. Larger datasets can determine whether these features retain their direction, how their relative importance should be weighted, and whether combinations identified here remain informative within more comprehensive longitudinal or multivariable analytical frameworks, including trajectory analyses, clustering approaches, or machine-learning models. This approach is consistent with frameworks emphasizing time-resolved, multiparametric host-response assessment (15, 16). Directionality should therefore be viewed as an interpretable exploratory simplification rather than as evidence that post-traumatic immune relationships are fundamentally binary or monotonic.

Our exploratory study has several limitations. The cohort was small, and the limited number of events for several endpoints restricts statistical power, nonlinear modeling, extensive multivariable adjustment, and subgroup analyses. Two reported multivariable combinations had an EPV of six, further limiting the precision and stability of their estimates. The primary screening comprised 144 directional tests (72 candidate biomarker variables assessed in two directions), and no formal multiplicity correction was applied in the context of exploratory screening and the discrete p-value structure of the small-sample quartile comparisons; all p values should therefore be interpreted as exploratory and unadjusted. The use of internally derived quartile thresholds further increases the possibility of chance findings and selection-induced optimism and limits direct transportability of the reported cut-offs.

The complete model-selection procedure was therefore additionally evaluated by bootstrap resampling. Selection reproducibility of the displayed multivariable combinations was limited, with inclusion probabilities of the displayed multivariable combinations ranging from 0.16 to 0.54, despite optimism-corrected AUCs ranging from 0.69 to 0.85. Selected individual biomarker associations showed higher inclusion probabilities. Thus, apparent discrimination and model-selection reproducibility were not interchangeable. The robustness of individual univariable associations also differed across sensitivity analyses, with selected biomarkers showing reduced selection reproducibility, lower optimism-corrected discrimination, limited coefficient stability, or attenuation after adjustment for baseline covariates. These limitations were not uniformly present across all candidate biomarkers. Continuous-biomarker sensitivity analyses showed consistent coefficient directions but did not uniformly retain nominal statistical evidence or discrimination comparable to the quartile-based models. Firth-penalized analyses generally preserved the direction of retained multivariable associations but yielded wide confidence intervals. These sensitivity analyses reduce, but do not eliminate, concerns regarding discretization and sparse-data bias and do not substitute for independent validation. Formal calibration was not performed because of the limited endpoint event numbers and because the retained combinations were not developed as clinical risk-prediction models. Accordingly, reported AUCs represent apparent within-cohort discrimination rather than externally validated predictive performance.

The longitudinal analysis also depended on a prespecified analytic completeness criterion. Of 62 enrolled patients, 52 were included; One patient died after the initial ER blood collection and clinical assessment and contributed the available ER data; measurements scheduled after death were considered structurally unavailable rather than missing. The remaining four patients who died survived beyond 96 h and had complete scheduled measurements. None of the 10 non-included patients died, and their available baseline characteristics showed no marked descriptive imbalance compared with the analyzed cohort. Nevertheless, detailed longitudinal biomarker and endpoint data were unavailable for these patients, and residual selection bias cannot be excluded.

Residual clinical confounding also remains relevant. Bivariate adjustment for ISS, age, or sex preserved many associations but attenuated selected signals and cannot establish independence from broader clinical confounding. Only 14 women were included, limiting sex-specific interpretation. Enrollment was also markedly imbalanced between the two study sites (49 versus 3 patients), precluding a meaningful assessment of center effects despite shared organizational structures and laboratory procedures. Detailed anatomical injury patterns and trauma mechanism were incompletely available, and operative treatment was documented in 48/52 patients and occurred early in most of these patients, limiting separation of injury-related from perioperative effects. In addition, the study’s prespecified exclusion criteria reduced major known comorbidity-related heterogeneity, but medical conditions beyond those systematically captured by the protocol could not be comprehensively reconstructed and may have contributed residual confounding.

Finally, the clinical endpoints were pragmatic: microbiological culture positivity was not equivalent to adjudicated infection, creatinine-defined renal impairment did not represent KDIGO-defined AKI, and hepatic enzyme elevation did not establish hepatic dysfunction. The exact timing of first microbiological culture positivity could not be reconstructed reliably, preventing a formal first-occurrence analysis for this endpoint. Early termination of PRIME recruitment during the COVID-19 pandemic further limited cohort size.

In summary, the evaluated clinical endpoints were associated with distinct immune–metabolic signatures following severe trauma. Microbiological culture positivity was associated with a serial pattern spanning early physiologic stress, adaptive immune alterations, and 96-h monocyte-related changes, whereas creatinine-defined renal impairment linked shock-associated metabolic disturbance with time-resolved cellular and inflammatory immune alterations. Berlin-defined ARDS and hepatic enzyme elevation showed predominantly low-odds patterns, while the hemodynamic endpoints were characterized by distinct high-odds configurations rather than a single common shock phenotype. Many individual components reflect established trauma and critical-illness biology; the hypothesis-generating contribution lies in their endpoint-specific organization and changing relative prominence across the serial post-traumatic continuum. These biologically informed candidate signatures require independent validation in larger cohorts to determine their reproducibility, relative weighting, and potential clinical relevance.

## Data Availability

The raw data supporting the conclusions of this article will be made available by the authors, without undue reservation.
