# Supplementary material for: Directional immuno–metabolic signatures following severe trauma are associated with organ-specific clinical endpoints in a prospective cohort
Source: Front Immunol. 2026 Oct 28;17:1964036. doi: 10.3389/fimmu.2026.1964036 (PMC13751341; doi:10.3389/fimmu.2026.1964036)
Supplement: Supplementary file 1 [file DataSheet1.pdf]

frontiers

The International Society for the Advancement of Cytometry (ISAC) has highlighted the importance of including comprehensive methodological information to ensure data reproducibility and reliability. In line with this, Frontiers in Immunology now requires authors to submit a checklist for manuscripts that involve flow or mass cytometry. This checklist helps standardize the reporting process, improving the quality and transparency of published data. By doing so, we support scientific progress, making it easier for other researchers to replicate and validate experiments.

This form should be submitted with any manuscripts using flow or mass cytometry.

Sample/specimen/material description

☑ Total blood

☐ PBMCs

☐ Organ digests

Other____

Did the samples suffer any treatment before or after incubation with the antibodies?

☐ Drug ____________________________________

☐ Cell permeabilization ______________________

☐ Dye _____________________________________

☐ Propidium iodine

☑ Not applicable

Other _______________________________________

Instrument and antibodies

Name of the Cytometer Beckman Coulter Cytoflex

Antibodies and targets	Fluorochrome/ Metal	Catalog number/Company
e.g. anti-CD4	FITC	Cat. XXX/ XXX ltd.
anti-CD14	PE-Cyanine7	25-0149-42 /Life Techn.

anti-HLA-DR	PE	12-9952-42 /Life Techn.
anti-CD11b	FITC	11-0118-42 /Life Techn.
anti-CD64	PerCP-eFluor™ 710	46-0649-42 /Life Techn.
anti-CD4	FITC	11-0049-42 /Life Techn.
anti-CD8a	PE-Cyanine7	25-0086-42 /Life Techn.
anti-CD25	PE-Cyanine5	15-0259-42 /Life Techn.
anti-CD127	PE	12-1278-42 /Life Techn.
anti-CD62p	PE	12-0626-80 /Life Techn.
pHrodo bioparticles	Deep Red	10581135 /Life Techn.

Data analyses

1. Name of the software Beckman Coulter CytExpert 1.1 ______
2. Reference gating strategy in the manuscript or supplementary material

Gating strategy in (eg Figure X) Suppl_Fig-1_Gating_Strategy_R1__
