# Supplementary material for: Directional immuno–metabolic signatures following severe trauma are associated with organ-specific clinical endpoints in a prospective cohort
Source: Front Immunol. 2026 Oct 28;17:1964036. doi: 10.3389/fimmu.2026.1964036 (PMC13751341; doi:10.3389/fimmu.2026.1964036)
Supplement: Supplementary file 2 [file DataSheet2.pdf]

Suppl. Fig. 1 A. Monocyte gating strategy

Representative CytExpert PDF exports from the monocyte panel. The fully stained sample is shown with an exemplar all-minus-HLA-DR FMO and an unstained control; histogram-only and duplicated views were omitted.

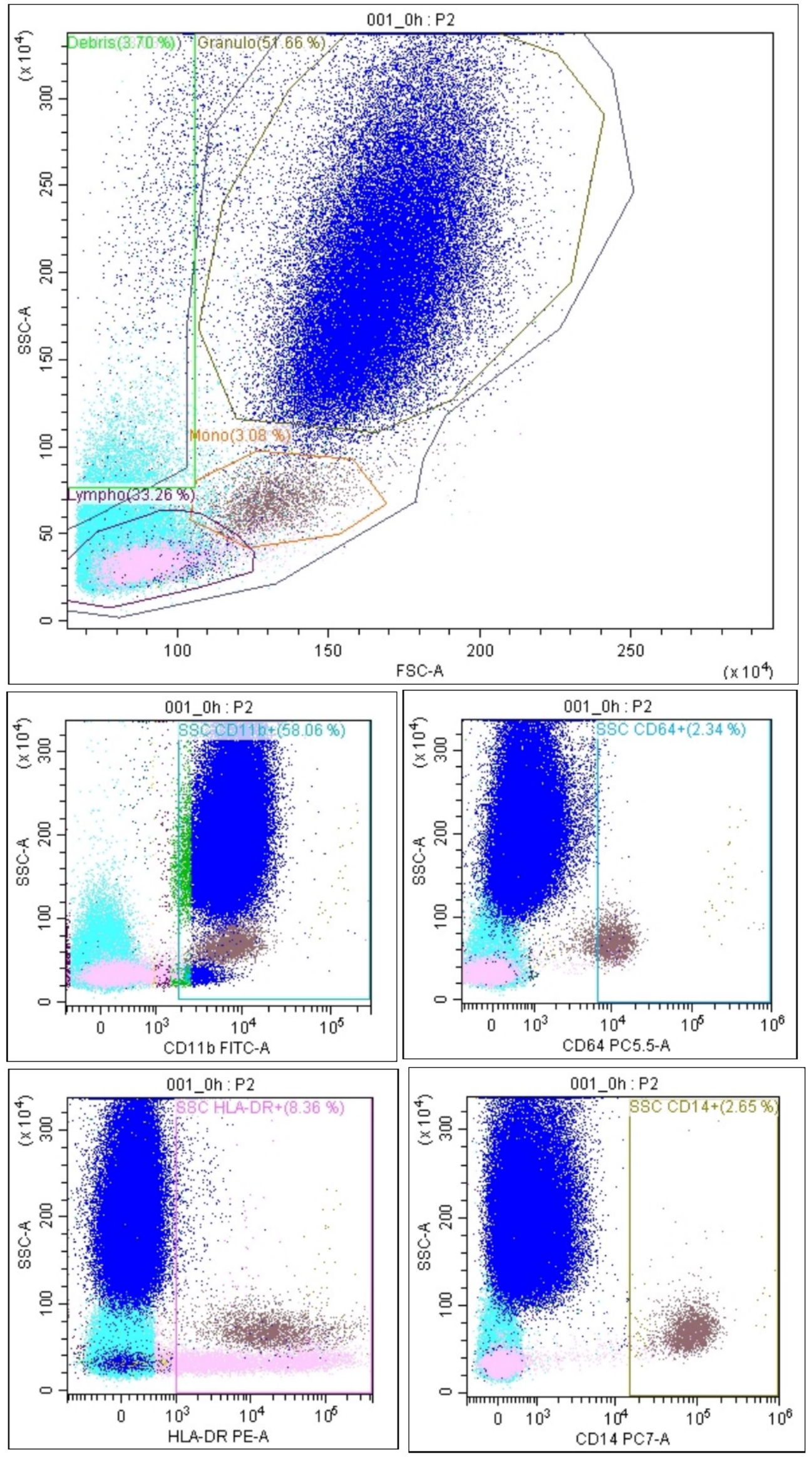

1. Fully stained monocyte panel showing the FSC/SSC population gates and downstream CD11b, CD64, HLA-DR, and CD14 marker gates.

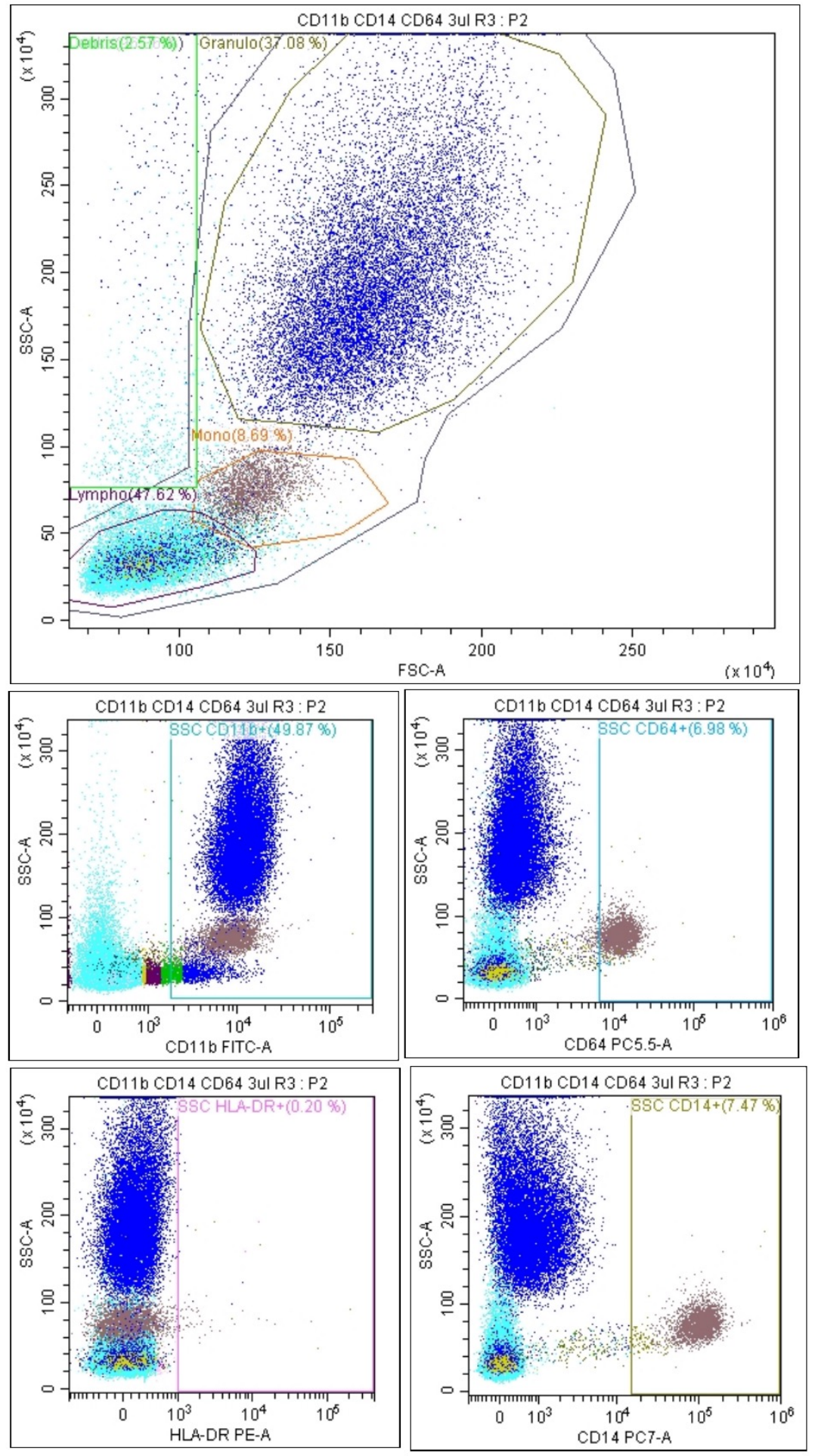

2. Representative all-minus-HLA-DR FMO control used for HLA-DR threshold orientation.

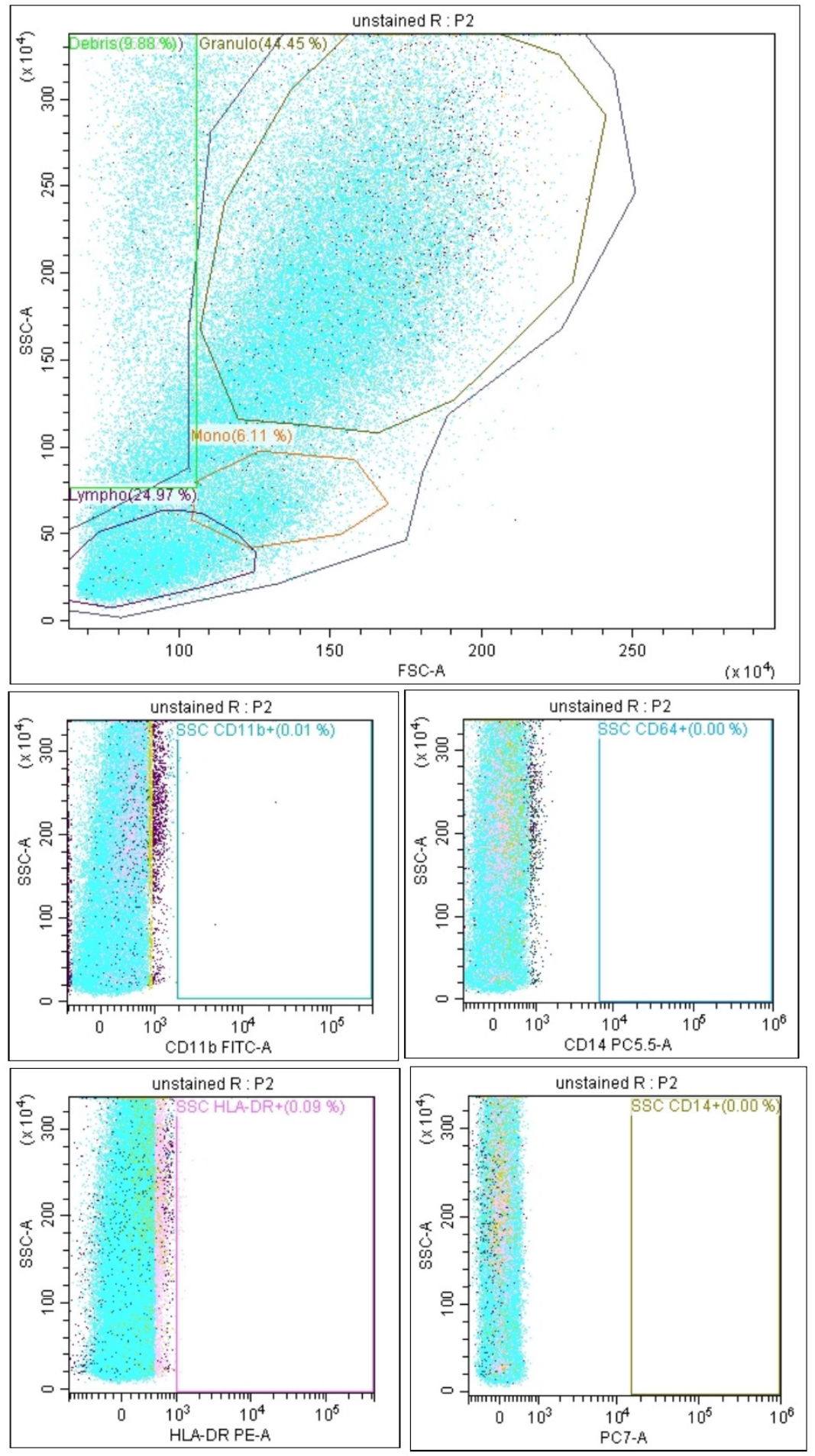

3. Unstained monocyte-panel control.

Histogram-only and tabular pages were omitted; all displayed panels show CytExpert exports.

Suppl. Fig. 1 B. Lymphocyte gating strategy

Representative CytExpert PDF exports from the lymphocyte panel. Two fully stained pages capture initial lymphocyte gating and downstream CD4/CD8/CD25/CD127 phenotyping; an all-minus-CD127 FMO and an unstained control are included.

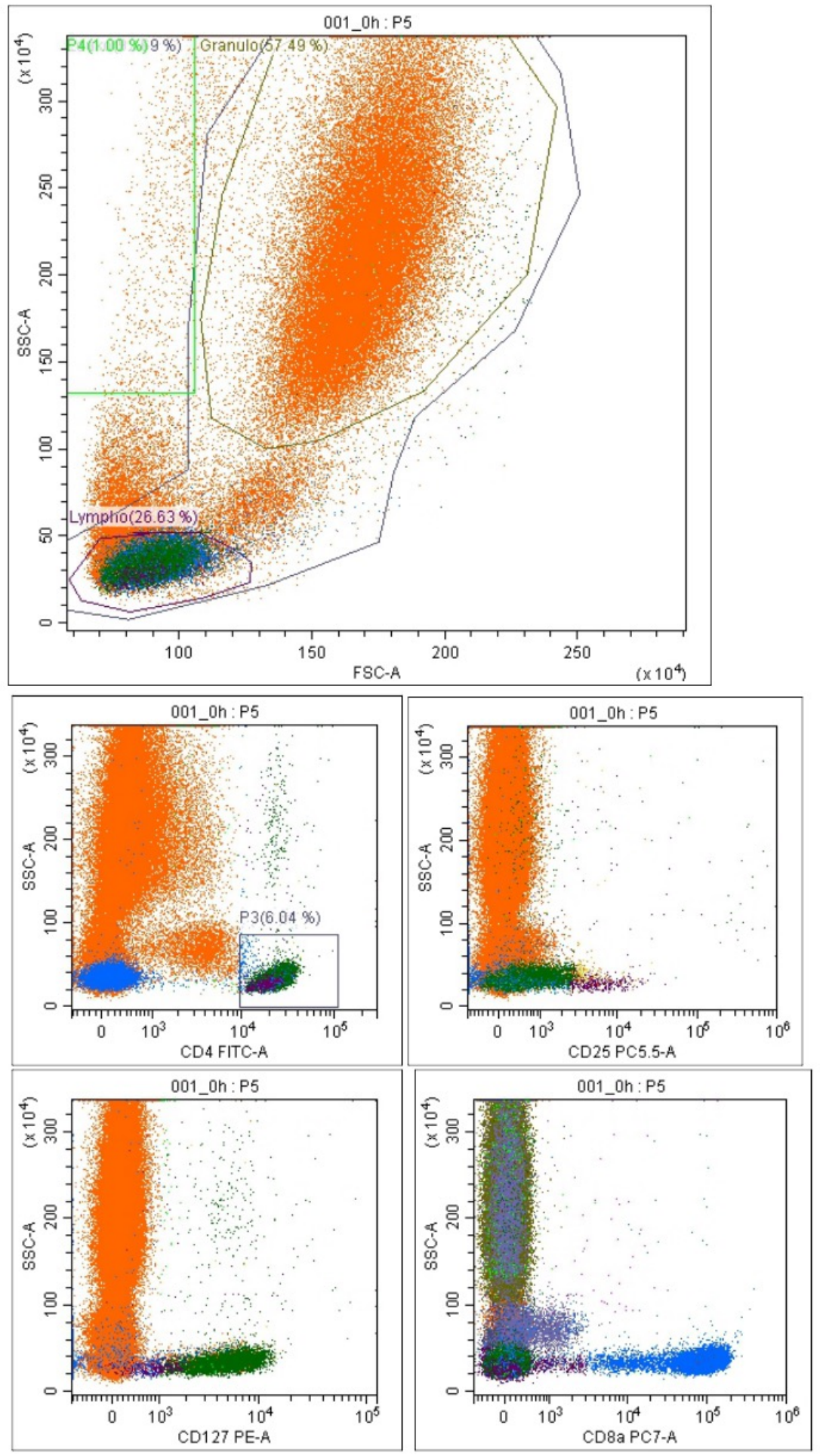

1. Fully stained lymphocyte panel showing the FSC/SSC lymphocyte gate and marker-specific overview plots.

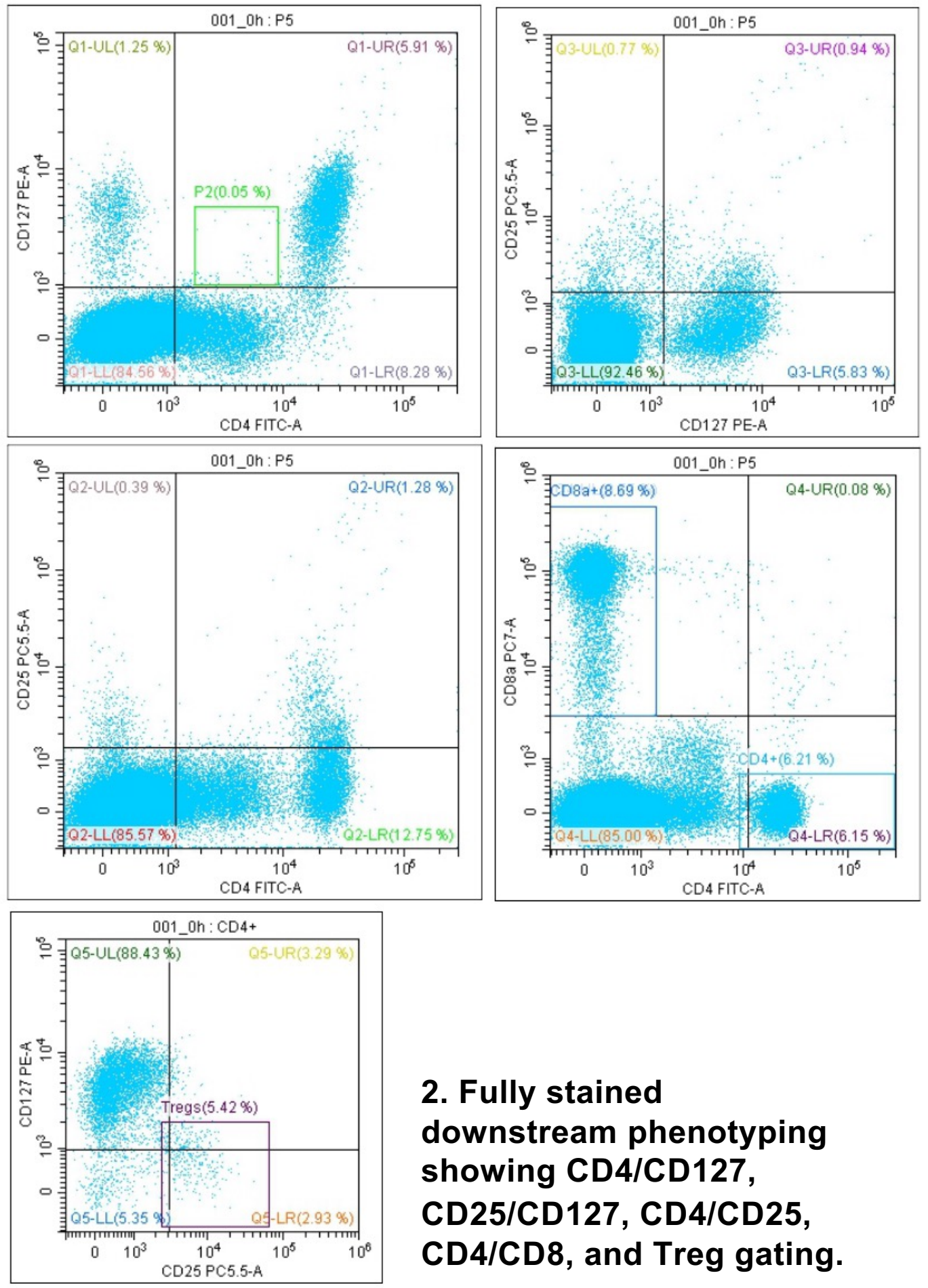

2. Fully stained downstream phenotyping showing CD4/CD127, CD25/CD127, CD4/CD25, CD4/CD8, and Treg gating.

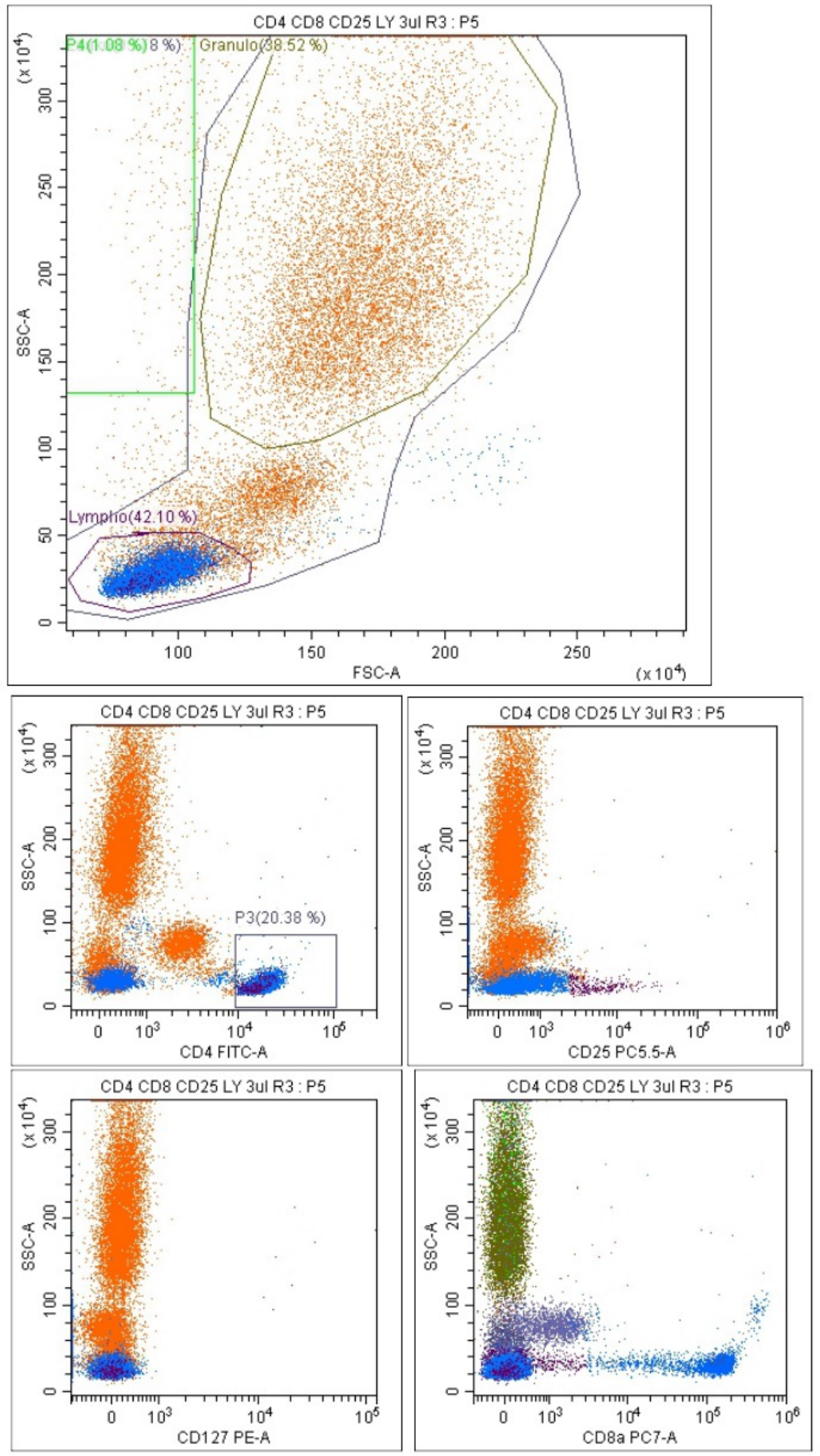

3. Representative all-minus-CD127 FMO control used for CD127 threshold orientation.

Controls are shown as representative examples where available; histogram-only panels and duplicated views were omitted.

Suppl. Fig. 1 C. Phagocytic capacity gating strategy

Representative CytExpert PDF exports from the phagocytosis assay. Ice-incubated and room-temperature conditions are shown together with representative e. coli pHrodo single-stained and PMA-activated controls.

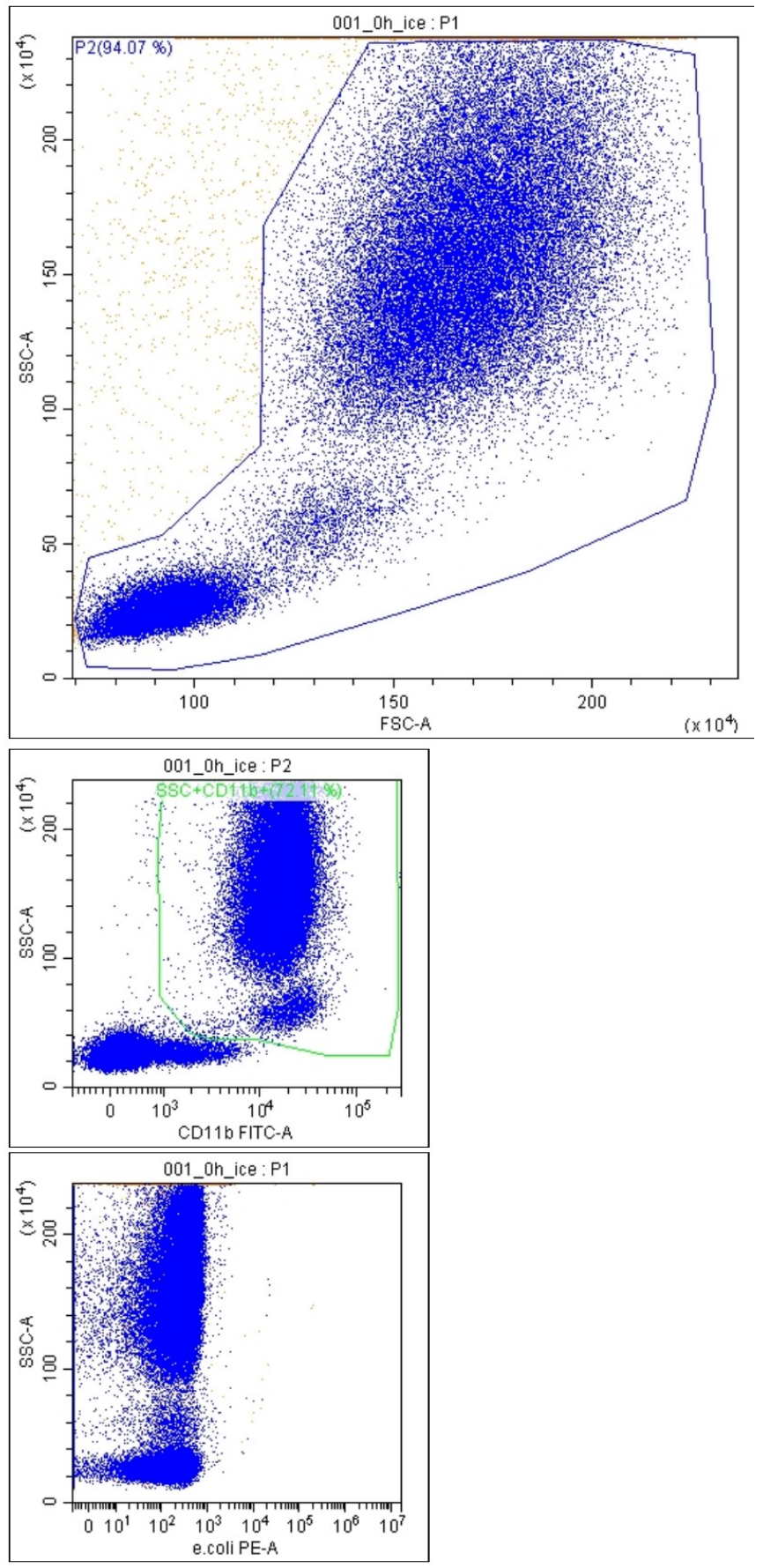

1. Ice-incubated sample showing initial gating, the CD11b+ phagocyte gate, and the e. coli pHrodo readout.

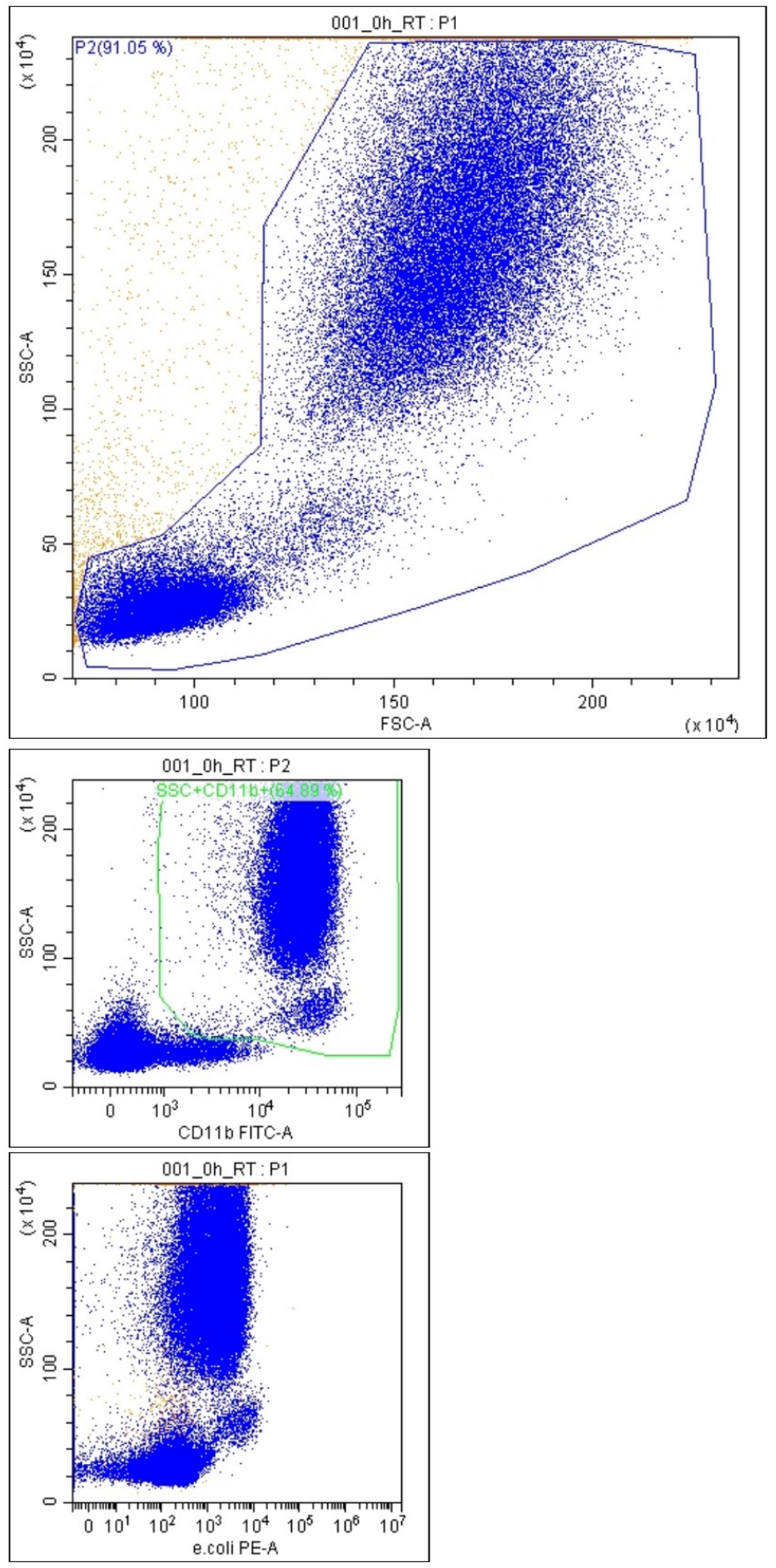

2. Room-temperature control showing the corresponding gating sequence and e. coli pHrodo uptake readout.

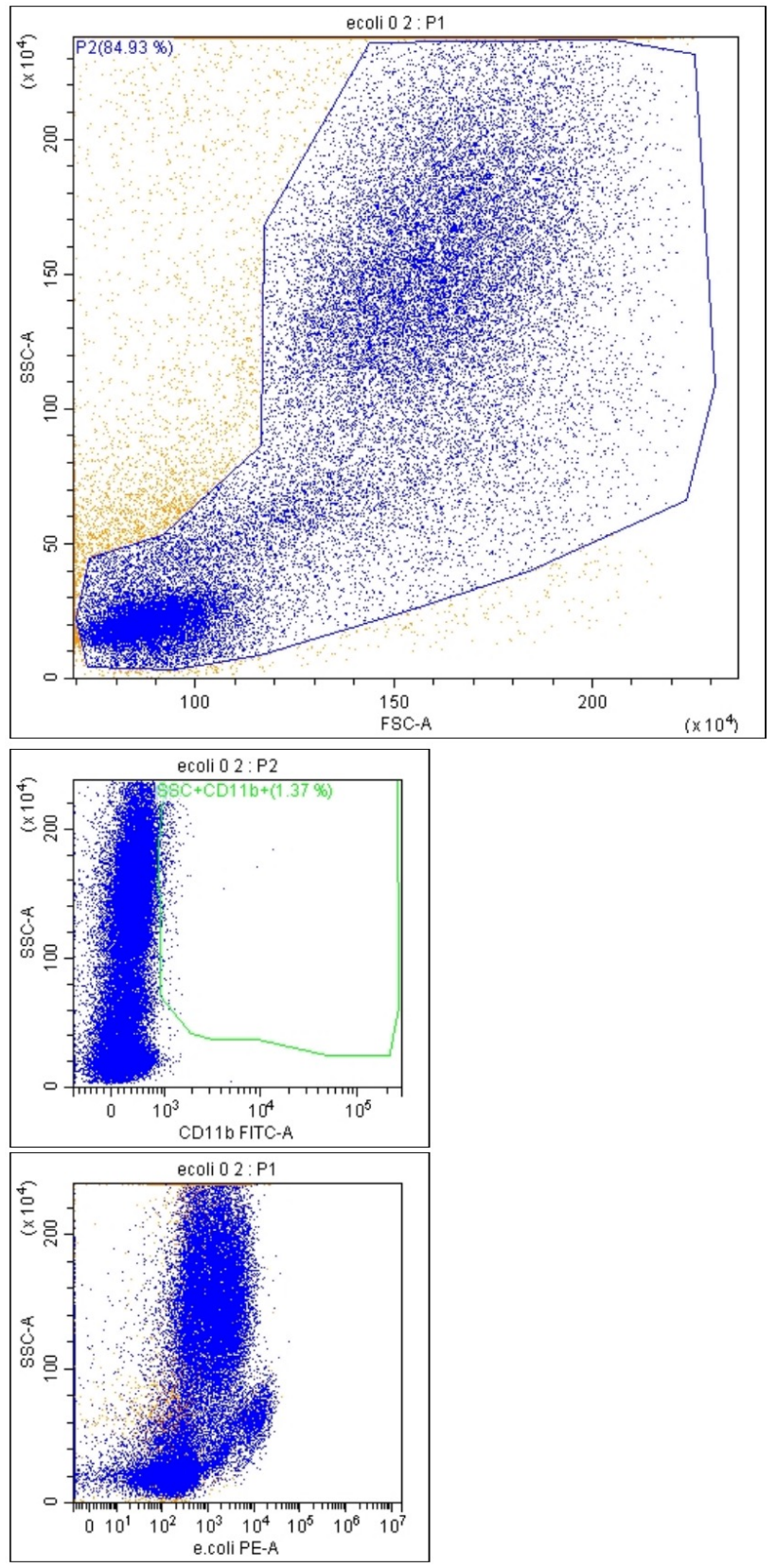

3. Representative e. coli pHrodo single-stained control.

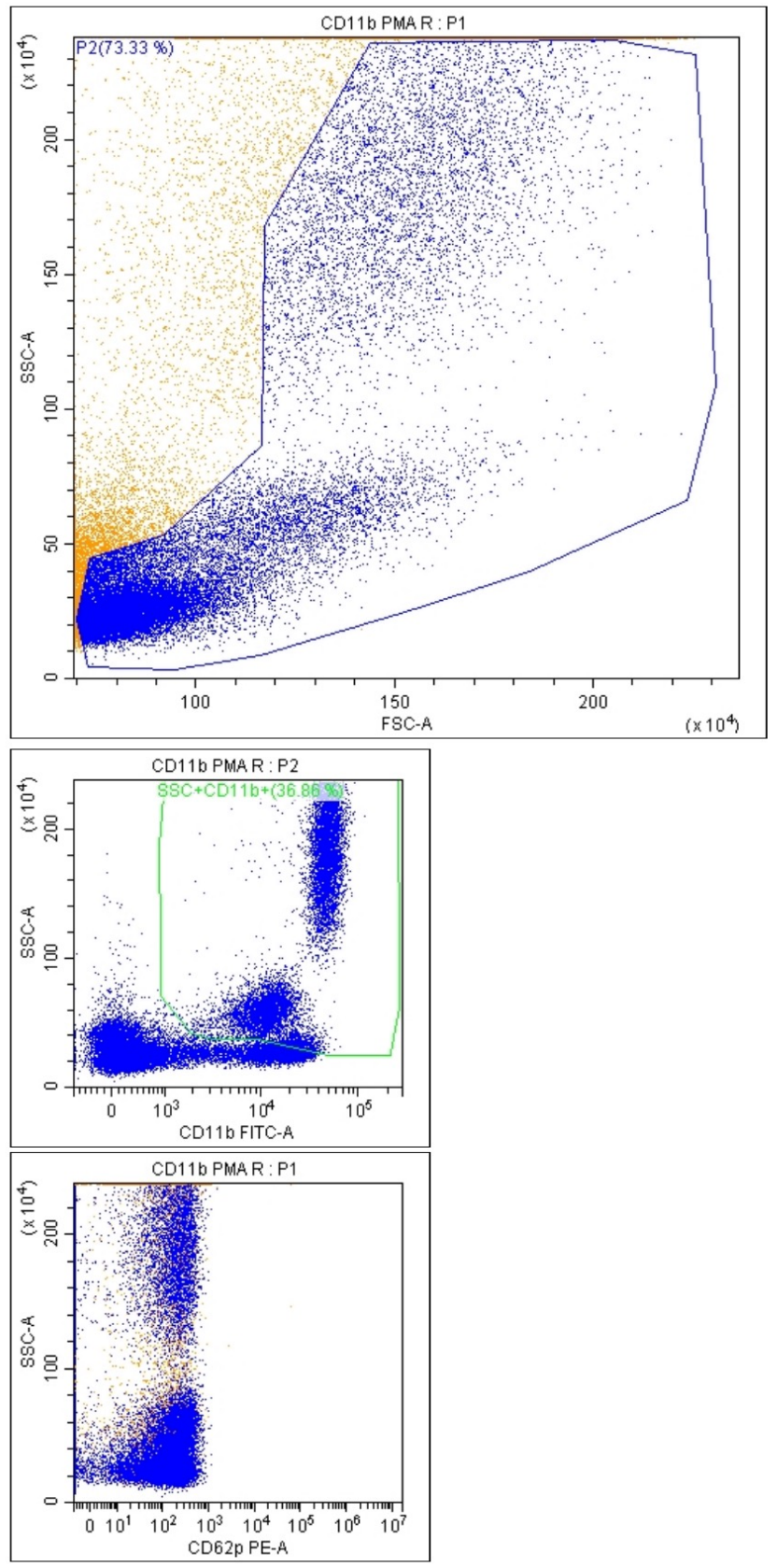

4. PMA-activated positive control.

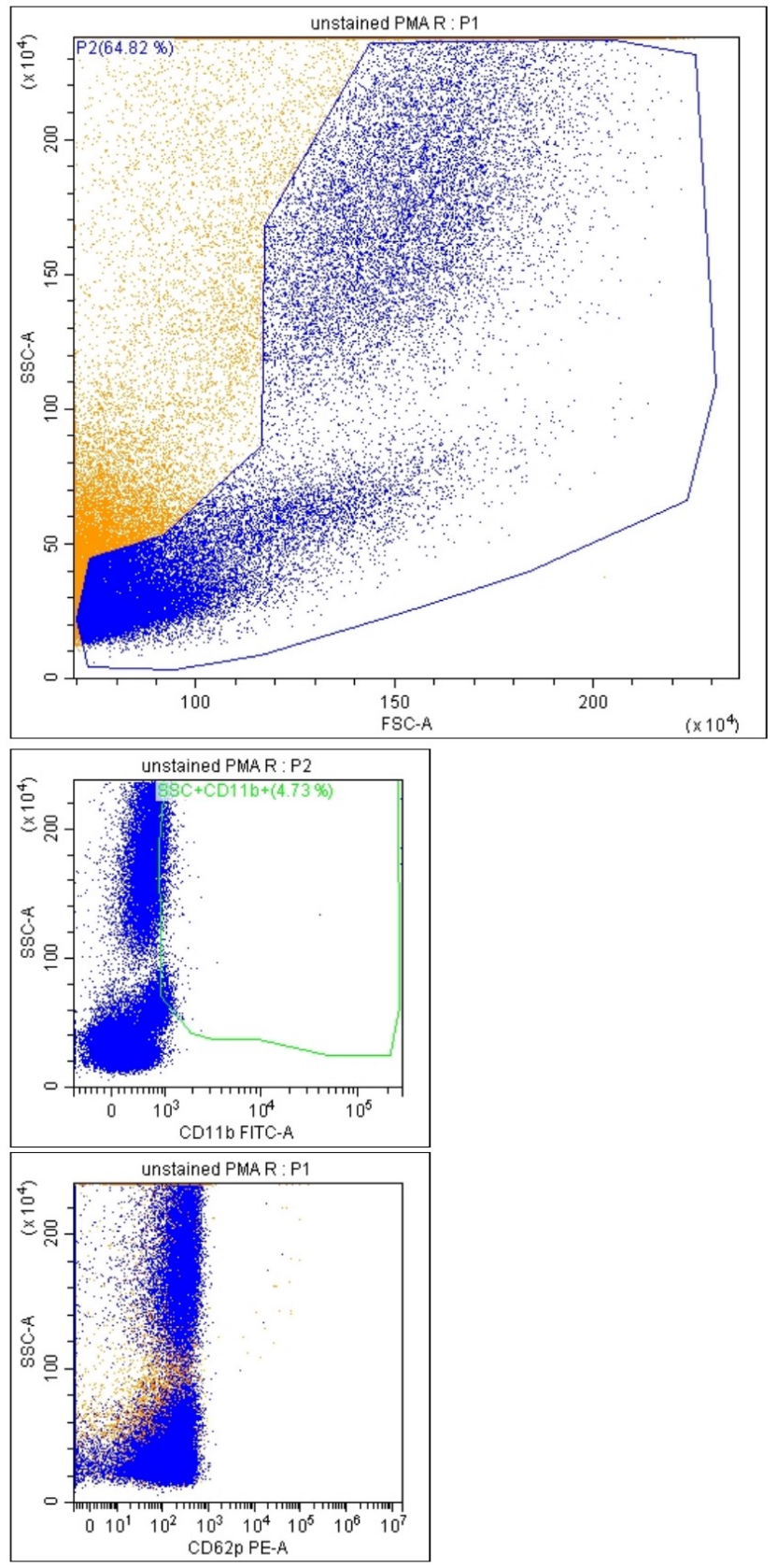

5. Unstained PMA-activated control.

Histogram-only and tabular pages were omitted; all displayed panels show CytExpert exports.

Suppl. Fig. 1 D. Platelet–leukocyte aggregate (PLA) gating strategy

Representative CytExpert PDF exports from the PLA panel. The fully stained sample shows CD11b/CD14-defined myeloid populations and CD62P/P-selectin-associated events; an all-minus-CD62P FMO and CD11b and CD14 single-stained controls are included.

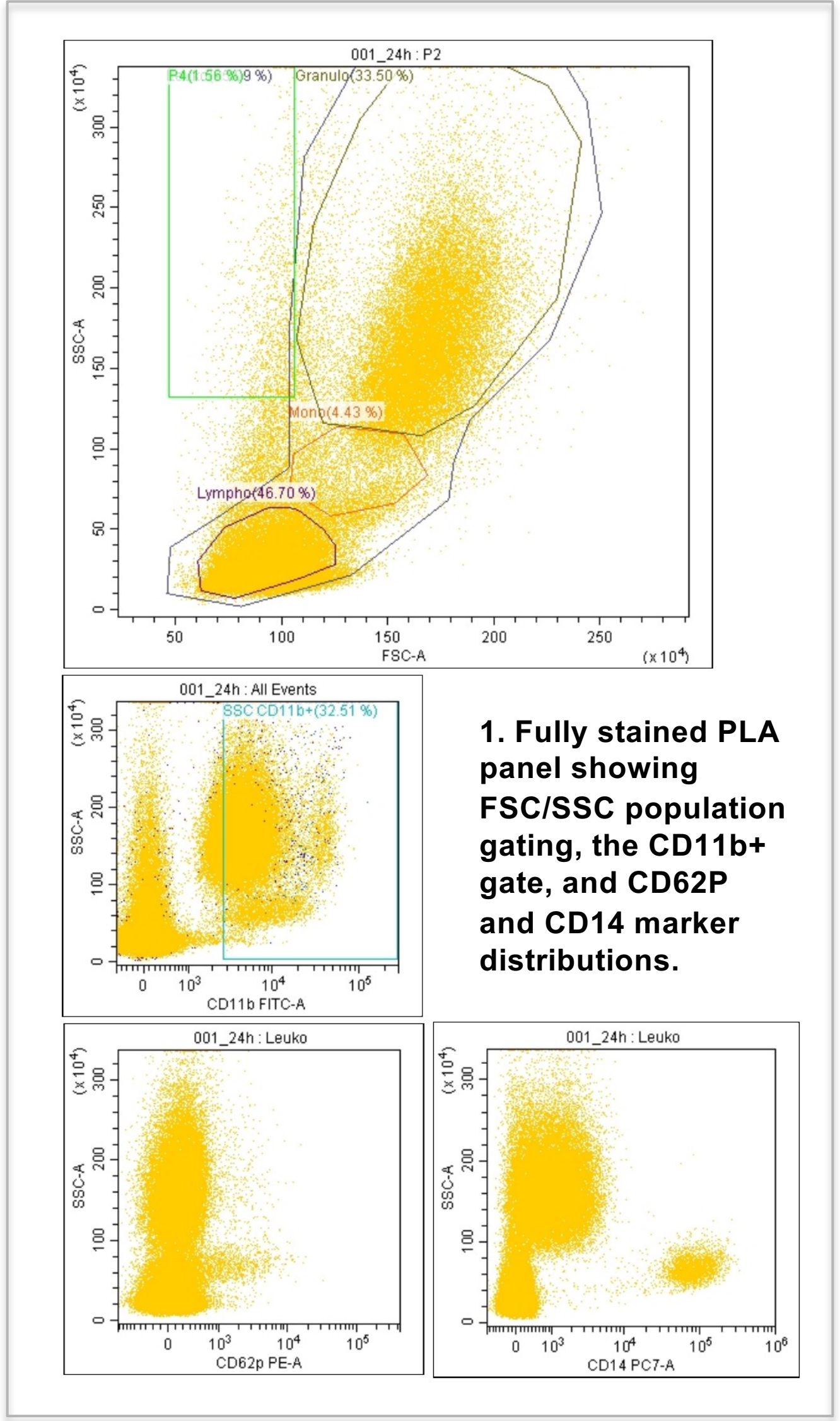

1. Fully stained PLA panel showing FSC/SSC population gating, the CD11b+ gate, and CD62P and CD14 marker distributions.

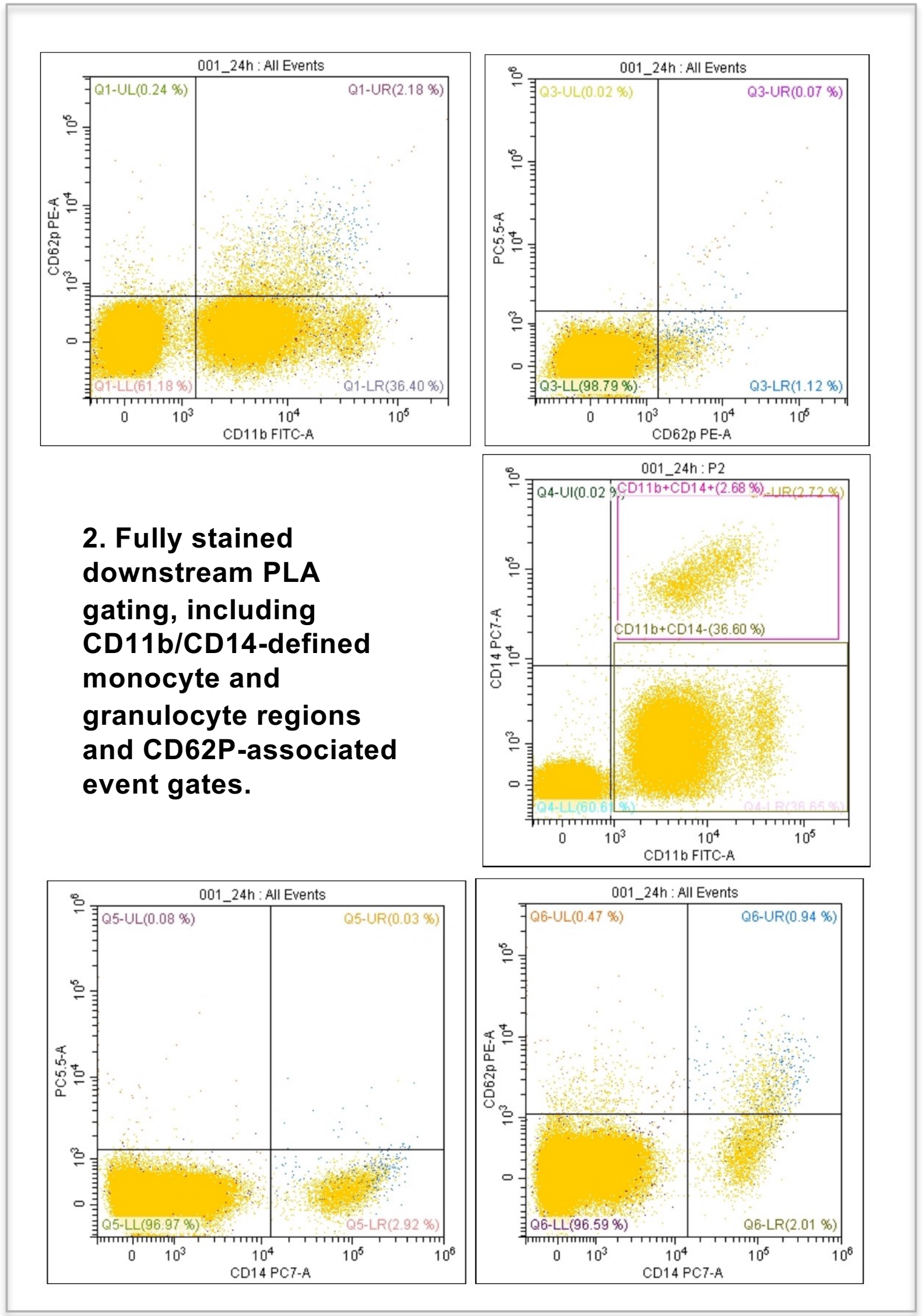

2. Fully stained downstream PLA gating, including CD11b/CD14-defined monocyte and granulocyte regions and CD62P-associated event gates.

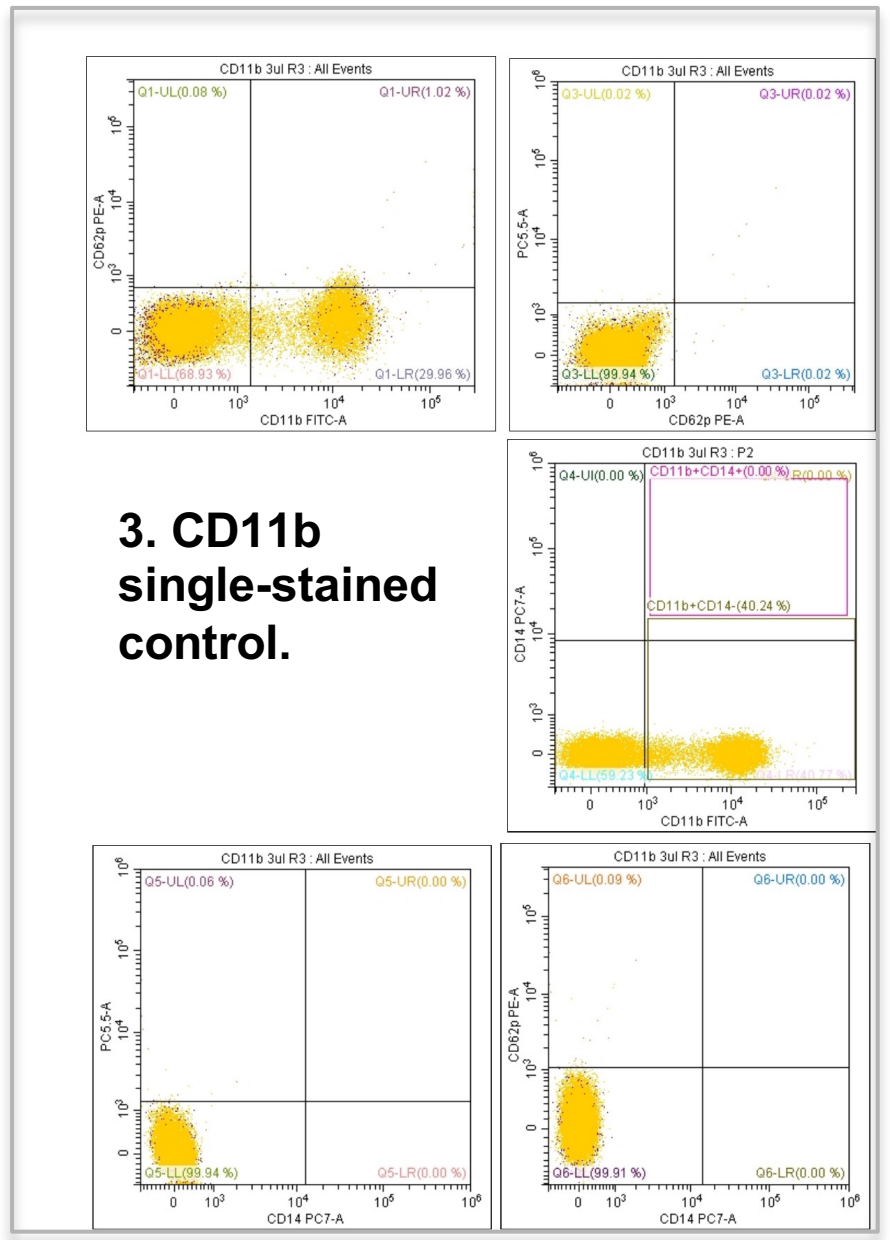

3. CD11b single-stained control.

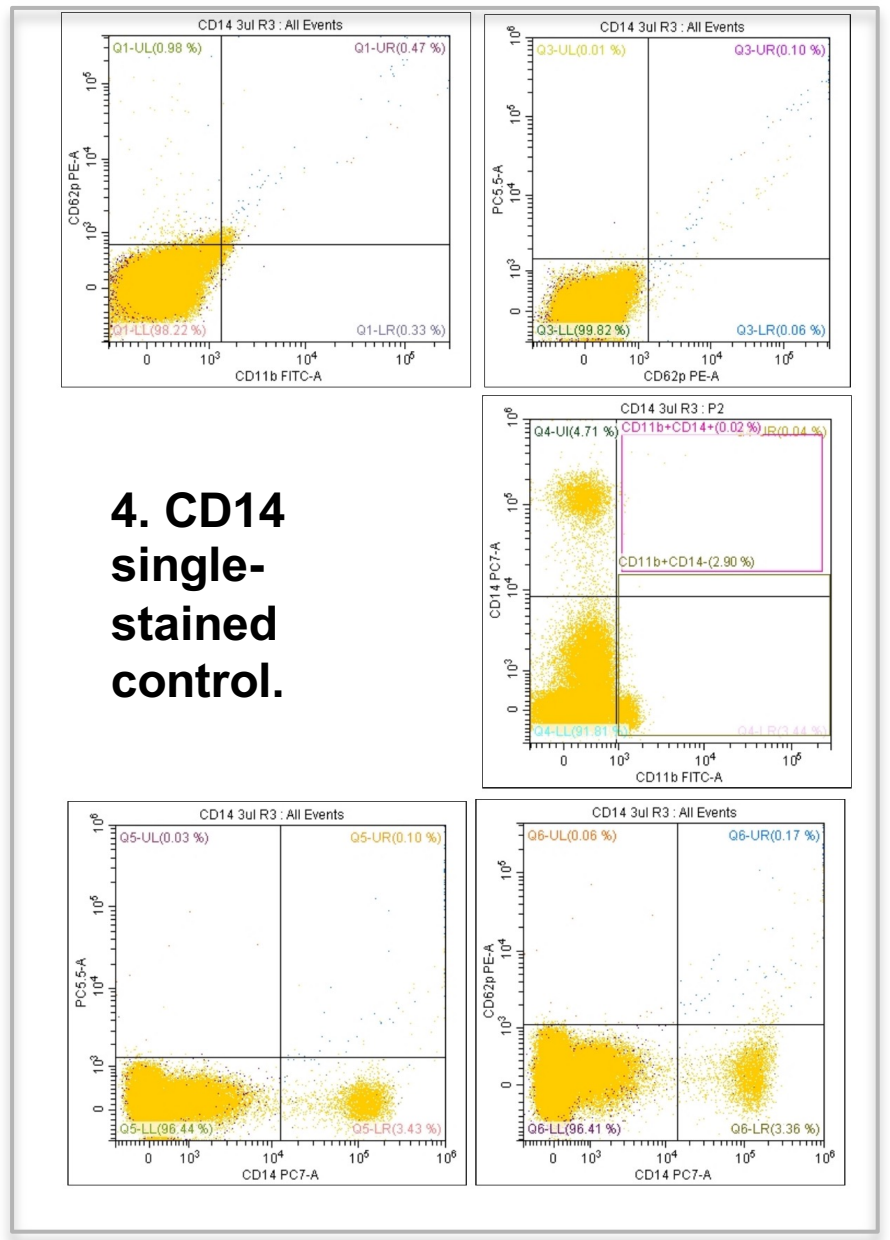

4. CD14 single-stained control.

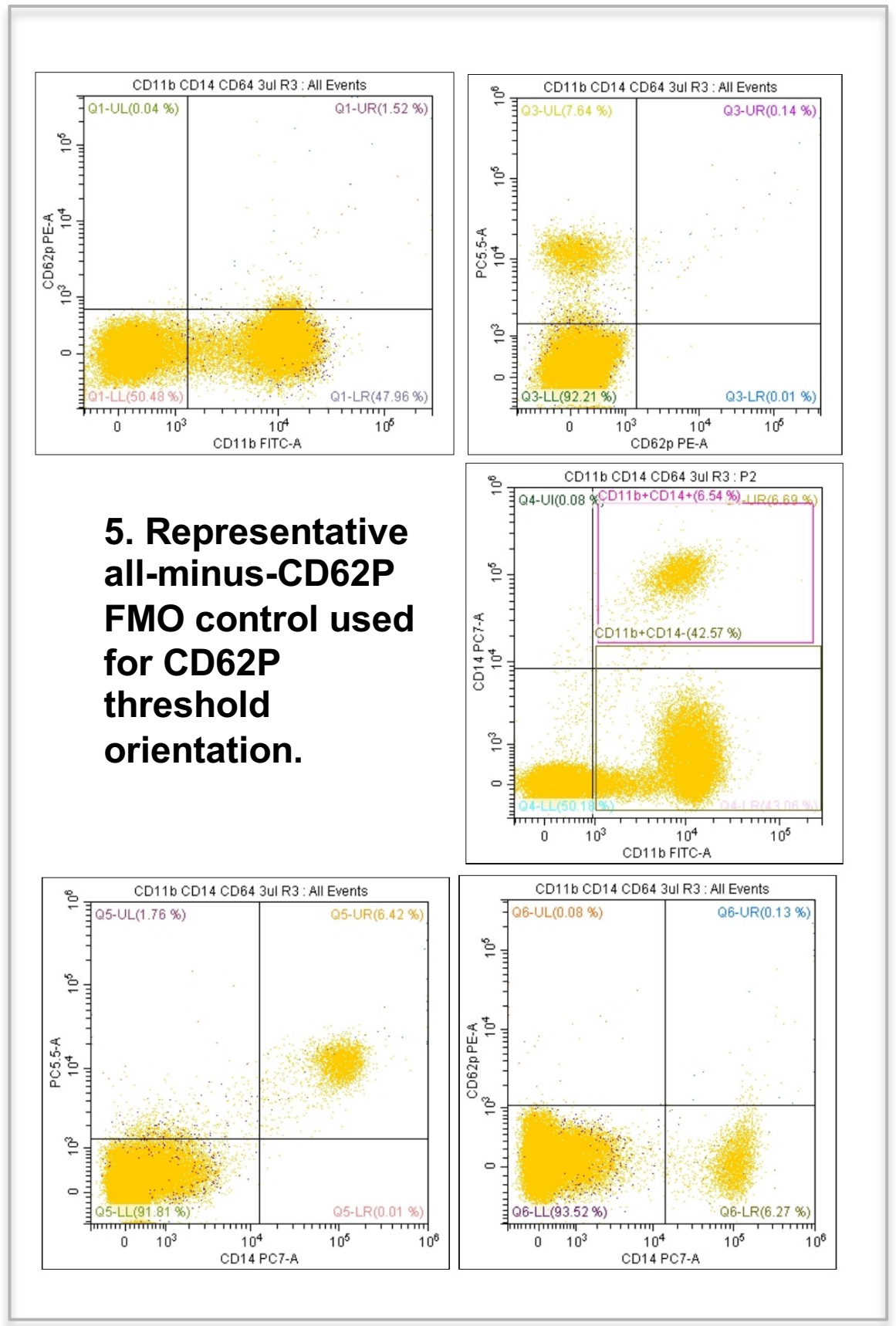

5. Representative all-minus-CD62P FMO control used for CD62P threshold orientation.

Histogram-only and tabular pages were omitted; all displayed panels show CytExpert exports.

Suppl. Fig. 1 E. Gating strategy: Common singlet-discrimination gate

A representative FSC-A versus FSC-H gate is shown once because the same singlet-discrimination strategy was applied before downstream gating in the monocyte, lymphocyte, phagocytosis, and platelet–leukocyte aggregate panels.

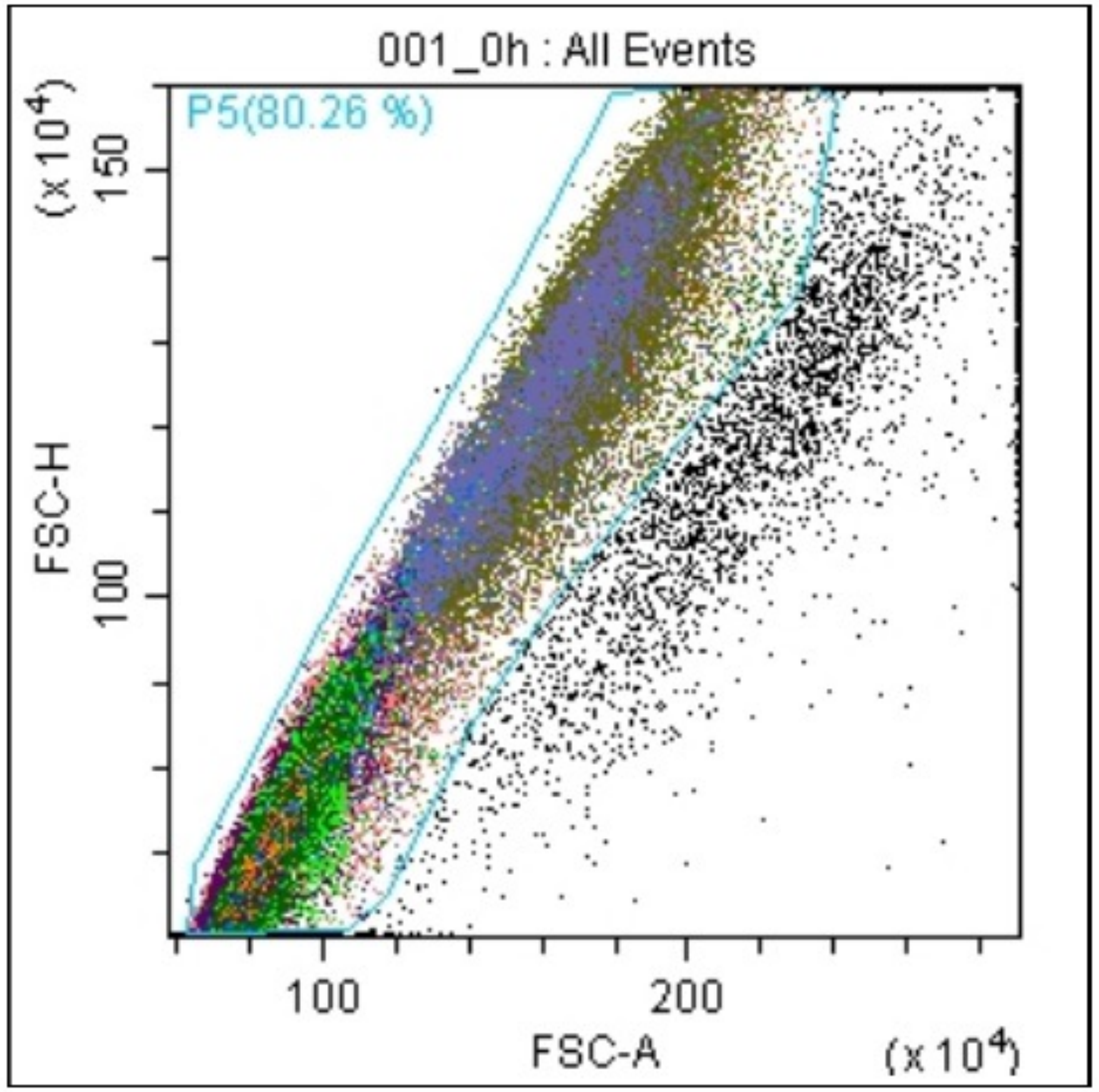

Representative FSC-A vs FSC-H singlet gate (P5), applied identically before panel-specific population and marker gating.

Supplementary Figure S1. Representative flow-cytometric gating strategies and assay controls. (A) Representative monocyte-panel gating, including FSC/SSC-based population gating and downstream assessment of CD11b, CD14, CD64, and HLA-DR, together with an exemplary all-minus-HLA-DR fluorescence-minus-one (FMO) control and an unstained control. (B) Representative lymphocyte-panel gating, including FSC/SSC-based lymphocyte identification and downstream CD4, CD8a, CD25, and CD127 phenotyping and regulatory T-cell gating, together with an exemplary all-minus-CD127 FMO control. (C) Representative phagocytic-capacity assay gating, including initial population gating, identification of CD11b-positive phagocytes, and the pHrodo-labeled E. coli readout. Ice-incubated and room-temperature conditions are shown together with a representative E. coli pHrodo single-stained control, a PMA-activated positive control, and an unstained PMA-activated control. (D) Representative platelet–leukocyte aggregate (PLA) gating, including FSC/SSC-based population gating, CD11b/CD14-defined myeloid populations, and CD62P/P-selectin-associated events, together with an exemplary all-minus-CD62P FMO control and CD11b and CD14 single-stained controls. (E) Representative FSC-A versus FSC-H singlet-discrimination gate. The same singlet-discrimination strategy was applied before panel-specific downstream gating in the monocyte, lymphocyte, phagocytosis, and PLA assays and is therefore shown once as a common gating step. All plots are representative CytExpert exports from the gating and control strategies used in the study.
