# Supplementary material for: Directional immuno–metabolic signatures following severe trauma are associated with organ-specific clinical endpoints in a prospective cohort
Source: Front Immunol. 2026 Oct 28;17:1964036. doi: 10.3389/fimmu.2026.1964036 (PMC13751341; doi:10.3389/fimmu.2026.1964036)
Supplement: Supplementary file 3 [file DataSheet3.pdf]

Event	Parameter	Time	</> threshold	unit	EPV	AIC	R^2	AUC	Youden I.	Sensitivity	Specifity	PPV	NPV	+LR	+LR	coeff. Intercept	coeff. Predictor	odds intercept	OR predictor	p intercept	p pred.
Hepatic enzyme elevation	Monocyte Median HLA-DR expression_upper +	96	> 10 855	MFI (a.u.)	16	54,23	0,36	0,78 [0,63; 0,88]	0,48	0,86	0,62	0,75	0,77	2,26	0,23	1,859 [0,912, 3,083]	-2,184 [-3,915; -0,663]	6,418 [2,489; 21,82]	0,113 [0,020; 0,516]	0,001	0,007
	+ CD64+ Phagocyte count_lower		< 24,08	Events/µL													-2,184 [-3,915; -0,663]		0,113 [0,020; 0,516]		0,007
	Phagocyte Mean Platelet signal	24	> 528	MFI (a.u.)	33	62,18	0,22	0,69 [0,55; 0,81]	0,46	0,77	0,69	0,88	0,50	2,48	0,33	1,204 [0,500, 2,009]	1,204 [0,500; 2,009]	3,333 [1,649; 7,455]	0,133 [0,030; 0,508]	0,002	0,005
	Monocyte Median HLA-DR expression	96	> 10 855	MFI (a.u.)	32	62,18	0,22	0,69 [0,56; 0,82]	0,46	0,77	0,69	0,88	0,50	1,77	0,33	1,204 [0,500, 2,009]	-2,015 [-3,513; -0,678]	3,333 [1,649; 7,455]	0,133 [0,030; 0,508]	0,002	0,005
	IL-1ra	48	< 59,06	pg/mL	32	63,85	0,18	0,68 [0,54; 0,81]	0,40	0,76	0,64	0,85	0,50	1,71	0,38	1,170 [0,462, 1,977]	-1,758 [-3,156; -0,471]	3,222 [1,588; 7,223]	0,172 [0,043; 0,624]	0,002	0,009
Microbiological culture positivity	C_I~Monocyte CBC_lower +	96	< 6,125	%	7	47,1	0,37	0,78 [0,60; 0,91]	0,48	0,65	0,83	0,73	0,77	3,82	0,42	-1,681 [-2,915, -0,714]	2,318 [0,724; 4,180]	0,186 [0,054; 0,490]	10,16 [2,063; 65,37]	0,002	0,007
	+ IL-8_upper		> 4,790	pg/mL													1,837 [0,117; 3,766]		6,276 [1,124; 43,21]		0,044
	CD4+ %	48	< 2,790	%	16	56,76	0,28	0,73 [0,59; 0,85]	0,51	0,69	0,82	0,56	0,89	3,83	0,38	-1,520 [-2,423, -0,762]	2,331 [0,959; 3,873]	0,219 [0,089; 0,467]	10,29 [2,610; 48,07]	<0,001	0,001
	Monocyte CBC	96	< 6,125	%	15	57,36	0,27	0,71 [0,58; 0,84]	0,54	0,73	0,81	0,50	0,92	3,84	0,33	-1,417 [-2,262, -0,695]	2,398 [0,945; 4,091]	0,242 [0,104; 0,499]	11,00 [2,574; 59,83]	<0,001	0,002
	Base Excess	0	< -2,70	mmol/L	16	60,90	0,18	0,68 [0,55; 0,82]	0,42	0,62	0,80	0,50	0,86	3,10	0,48	-1,355 [-2,203, -0,627]	1,825 [0,497; 3,257]	0,258 [0,110; 0,534]	6,200 [1,644; 25,96]	0,001	0,009
	CD4/CD8 ratio	24	< 0,959	-	16	60,90	0,18	0,68 [0,54; 0,83]	0,42	0,62	0,80	0,50	0,86	3,10	0,48	-1,355 [-2,203, -0,627]	1,825 [0,497; 3,257]	0,258 [0,110; 0,534]	6,200 [1,644; 25,96]	0,001	0,009
	CD4/CD8 ratio	48	< 1,201	-	16	60,90	0,18	0,68 [0,55; 0,82]	0,42	0,62	0,80	0,50	0,86	3,10	0,48	-1,355 [-2,203, -0,627]	1,825 [0,497; 3,257]	0,258 [0,110; 0,534]	6,200 [1,644; 25,96]	0,001	0,009
	IL-8	96	> 4,790	pg/mL	16	60,90	0,18	0,68 [0,55; 0,82]	0,42	0,62	0,80	0,50	0,86	3,10	0,48	-1,355 [-2,203, -0,627]	1,825 [0,497; 3,257]	0,258 [0,110; 0,534]	6,200 [1,644; 25,96]	0,001	0,009
	Monocyte Count	96	< 26,04	Events/µL	16	60,90	0,18	0,68 [0,54; 0,82]	0,42	0,62	0,80	0,50	0,86	3,10	0,48	-1,355 [-2,203, -0,627]	1,825 [0,497; 3,257]	0,258 [0,110; 0,534]	6,200 [1,644; 25,96]	0,001	0,009
	Monocyte Median HLA-DR expression	96	< 5 186	MFI (a.u.)	16	60,90	0,18	0,68 [0,53; 0,82]	0,42	0,62	0,80	0,50	0,86	3,10	0,48	-1,355 [-2,203, -0,627]	1,825 [0,497; 3,257]	0,258 [0,110; 0,534]	6,200 [1,644; 25,96]	0,001	0,009
	Body temperature	0	< 35,60	°C	16	62,24	0,15	0,67 [0,53; 0,80]	0,36	0,57	0,79	0,50	0,83	2,71	0,54	-1,723 [-2,803, -0,858]	2,175 [0,886; 3,604]	0,267 [0,114; 0,554]	5,000 [1,373; 19,65]	0,001	0,016
Creatinine-defined renal impairment	IL-8_upper +	0	> 6,690	pg/mL	6	38,4	0,57	0,88 [0,72; 0,95]	0,54	0,67	0,87	0,62	0,89	5,15	0,38	-3,446 [-6,334, -1,890]	3,284 [1,320; 6,323]	0,032 [0,002; 0,151]	26,68 [3,744; 557,3]	0,001	0,005
	+ CD4/CD8 ratio_lower		< 0,768	-													3,514 [1,527; 6,574]		33,58 [4,605; 715,9]		0,003
	IL-8	0	> 6,690	pg/mL	14	56,76	0,25	0,72 [0,57; 0,86]	0,47	0,62	0,85	0,57	0,87	4,13	0,45	-1,705 [-2,679, -0,908]	2,175 [0,803; 3,673]	0,182 [0,069; 0,403]	8,800 [2,232; 39,37]	<0,001	0,003
	CD4/CD8 ratio	0	< 0,768	-	13	57,36	0,25	0,72 [0,56; 0,85]	0,47	0,62	0,85	0,57	0,87	4,13	0,45	-1,705 [-2,679, -0,908]	2,175 [0,803; 3,673]	0,182 [0,069; 0,403]	8,800 [2,232; 39,37]	<0,001	0,003
	CD4+ %	48	< 2,790	%	13	60,90	0,25	0,72 [0,57; 0,84]	0,47	0,62	0,85	0,57	0,87	4,13	0,45	-1,705 [-2,679, -0,908]	2,175 [0,803; 3,673]	0,182 [0,069; 0,403]	8,800 [2,232; 39,37]	<0,001	0,003
	Monocyte Median CD64 expression	96	> 19 923	MFI (a.u.)	13	60,90	0,25	0,72 [0,56; 0,85]	0,47	0,62	0,85	0,57	0,87	4,13	0,45	-1,705 [-2,679, -0,908]	2,175 [0,803; 3,673]	0,182 [0,069; 0,403]	8,800 [2,232; 39,37]	<0,001	0,003
	pH	0	< 7,271	-	14	60,90	0,21	0,71 [0,55; 0,85]	0,41	0,57	0,84	0,57	0,84	3,56	0,51	-1,674 [-2,650, -0,874]	1,962 [0,624; 3,401]	0,188 [0,071; 0,417]	7,111 [1,867; 29,99]	<0,001	0,005
	Lymphocyte CBC	96	< 11,03	%	11	60,90	0,22	0,70 [0,56; 0,85]	0,47	0,64	0,83	0,50	0,90	3,76	0,43	-1,580 [-2,481, -0,828]	2,140 [0,712; 3,710]	0,206 [0,084; 0,437]	8,500 [2,038; 40,87]	<0,001	0,004
	Neutrophil CBC	96	> 78,48	%	11	60,90	0,22	0,70 [0,56; 0,83]	0,47	0,64	0,83	0,50	0,90	3,76	0,43	-1,580 [-2,481, -0,828]	2,140 [0,712; 3,710]	0,206 [0,084; 0,437]	8,500 [2,038; 40,87]	<0,001	0,004
	IL-10	0	> 8,540	pg/mL	14	60,90	0,16	0,67 [0,54; 0,81]	0,36	0,54	0,82	0,50	0,84	3,00	0,56	-1,520 [-2,423, -0,762]	1,674 [0,328; 3,090]	0,219 [0,089; 0,467]	5,333 [1,388; 21,98]	<0,001	0,016
	Base Excess	0	< -2,70	mmol/L	14	62,24	0,16	0,67 [0,53; 0,83]	0,36	0,54	0,82	0,50	0,84	3,00	0,56	-1,520 [-2,423, -0,762]	1,674 [0,328; 3,090]	0,219 [0,089; 0,467]	5,333 [1,388; 21,98]	<0,001	0,016
	IL-6	0	> 46,70	pg/mL	14	58,65	0,16	0,67 [0,51; 0,82]	0,36	0,54	0,82	0,50	0,84	3,00	0,56	-1,520 [-2,423, -0,762]	1,674 [0,328; 3,090]	0,219 [0,089; 0,467]	5,333 [1,388; 21,98]	<0,001	0,016
	% Monocytes	0	< 2,340	%	13	58,65	0,16	0,67 [0,50; 0,81]	0,36	0,54	0,82	0,50	0,84	3,00	0,56	-1,520 [-2,423, -0,762]	1,674 [0,328; 3,090]	0,219 [0,089; 0,467]	5,333 [1,388; 21,98]	<0,001	0,016
	IL-8	48	> 2,820	pg/mL	12	58,65	0,16	0,67 [0,52; 0,83]	0,36	0,54	0,82	0,50	0,84	3,00	0,56	-1,520 [-2,423, -0,762]	1,674 [0,328; 3,090]	0,219 [0,089; 0,467]	5,333 [1,388; 21,98]	<0,001	0,016
	CD4+ cell count	48	< 22,79	Events/µL	13	58,65	0,16	0,67 [0,53; 0,81]	0,36	0,54	0,82	0,50	0,84	3,00	0,56	-1,520 [-2,423, -0,762]	1,674 [0,328; 3,090]	0,219 [0,089; 0,467]	5,333 [1,388; 21,98]	<0,001	0,016
	IL-1ra	48	> 166,3	pg/mL	12	58,65	0,16	0,67 [0,49; 0,80]	0,36	0,54	0,82	0,50	0,84	3,00	0,56	-1,520 [-2,423, -0,762]	1,674 [0,328; 3,090]	0,219 [0,089; 0,467]	5,333 [1,388; 21,98]	<0,001	0,016
	IL-8	96	> 4,790	pg/mL	12	58,65	0,16	0,67 [0,53; 0,82]	0,36	0,54	0,82	0,50	0,84	3,00	0,56	-1,520 [-2,423, -0,762]	1,674 [0,328; 3,090]	0,219 [0,089; 0,467]	5,333 [1,388; 21,98]	<0,001	0,016
	% Granulocytes	96	> 81,86	%	13	58,65	0,16	0,67 [0,53; 0,82]	0,36	0,54	0,82	0,50	0,84	3,00	0,56	-1,520 [-2,423, -0,762]	1,674 [0,328; 3,090]	0,22 [0,09; 0,47]	5,33 [1,39; 22,0]	<0,001	0,016
	Monocyte Median HLA-DR expression	96	< 5 186	MFI (a.u.)	13	58,65	0,16	0,67 [0,52; 0,81]	0,36	0,54	0,82	0,50	0,84	3,00	0,56	-1,520 [-2,423, -0,762]	1,674 [0,328; 3,090]	0,219 [0,089; 0,467]	5,333 [1,388; 21,98]	<0,001	0,016
	IL-1ra	96	> 361,2	pg/mL	12	58,65	0,16	0,67 [0,52; 0,81]	0,36	0,54	0,82	0,50	0,84	3,00	0,56	-1,520 [-2,423, -0,762]	1,674 [0,328; 3,090]	0,219 [0,089; 0,467]	5,333 [1,388; 21,98]	<0,001	0,016
	Granulocyte Median CD64 expression	96	> 1 590	MFI (a.u.)	13	58,65	0,16	0,67 [0,51; 0,81]	0,36	0,54	0,82	0,50	0,84	3,00	0,56	-1,520 [-2,423, -0,762]	1,674 [0,328; 3,090]	0,219 [0,089; 0,467]	5,333 [1,388; 21,98]	<0,001	0,016
	MCP-1	96	> 37,63	pg/mL	12	58,65	0,16	0,67 [0,51; 0,82]	0,36	0,54	0,82	0,50	0,84	3,00	0,56	-1,520 [-2,423, -0,762]	1,674 [0,328; 3,090]	0,219 [0,089; 0,467]	5,333 [1,388; 21,98]	<0,001	0,016
Berlin-defined ARDS	Phagocyte Median CD64 expression_lower +	96	< 13 596	MFI (a.u.)	14	41,92	0,39	0,80 [0,6; 0,91]	0,47	0,91	0,56	0,71	0,83	2,07	0,16	2,386 [1,156, 4,222]	-2,483 [-4,782; -0,577]	10,87 [3,176; 68,15]	0,083 [0,008; 0,561]	0,001	0,017
	+ % Lymphocytes_lower		< 11,64	%													-2,444 [-4,557; -0,745]		0,087 [0,010; 0,475]		0,009
	pH	24	> 7,441	-	28	45,08	0,25	0,72 [0,55; 0,87]	0,47	0,83	0,64	0,86	0,58	2,31	0,27	1,569 [0,687, 2,657]	-2,128 [-3,796; -0,624]	4,800 [1,988; 14,26]	0,119 [0,022; 0,536]	0,001	0,008
	% Lymphocytes	96	< 11,64	%	28	47,82	0,17	0,69 [0,50; 0,84]	0,36	0,82	0,54	0,79	0,58	1,78	0,33	1,482 [0,590, 2,576]	-1,636 [-3,168; -0,208]	4,400 [1,804; 13,14]	0,195 [0,042; 0,812]	0,003	0,028
	pH	0	> 7,368	-	28	47,48	0,18	0,68 [0,52; 0,84]	0,40	0,80	0,60	0,86	0,50	2,00	0,33	1,386 [0,557, 2,379]	-1,792 [-3,431; -0,278]	4,000 [1,745; 10,80]	0,167 [0,032; 0,757]	0,002	0,023
	CD4/CD8 ratio	48	> 2,072	-	28	47,48	0,18	0,68 [0,51; 0,83]	0,40	0,80	0,60	0,86	0,50	2,00	0,33	1,386 [0,557, 2,379]	-1,792 [-3,431; -0,278]	4,000 [1,745; 10,80]	0,167 [0,032; 0,757]	0,002	0,023
	Phagocyte Mean Platelet signal	24	> 528,0	MFI (a.u.)	28	48,73	0,14	0,66 [0,50; 0,82]	0,34	0,79	0,55	0,82	0,50	1,76	0,38	1,344 [0,509, 2,340]	-1,526 [-3,079; -0,057]	3,83 [1,66; 10,4]	0,22 [0,05; 0,95]	0,003	0,045
	Phagocytic capacity	24	> 51,66	%	28	48,73	0,14	0,66 [0,48; 0,81]	0,34	0,79	0,55	0,82	0,50	1,76	0,38	1,344 [0,509, 2,340]	-1,526 [-3,079; -0,057]	3,833 [1,663; 10,38]	0,217 [0,046; 0,945]	0,003	0,045
	Neutrophil CBC	48	> 82,18	%	24	48,73	0,14	0,66 [0,49; 0,81]	0,34	0,79	0,55	0,82	0,50	1,76	0,38	1,344 [0,509, 2,340]	-1,526 [-3,079; -0,057]	3,833 [1,663; 10,38]	0,217 [0,046; 0,945]	0,003	0,045
Tachycardia	Lymphocyte CBC_lower +	96	< 11,03	%	6	42,74	0,40	0,80 [0,61; 0,92]	0,50	0,70	0,80	0,54	0,89	3,50	0,38	-2,053 [-3,511, -0,976]	2,310 [0,612; 4,292]	0,128 [0,030; 0,377]	10,08 [1,845; 73,14]	0,001	0,012
	+ CD8a+ cell count_lower		< 19,17	Events/µL													1,986 [0,322; 3,873]		7,289 [1,380; 48,09]		0,025
	IL-10	0	> 8,540	pg/mL	18	51,87	0,43	0,78 [0,65; 0,88]	0,67	0,85	0,82	0,61	0,94	4,72	0,18	-1,520 [-2,423, -0,762]	3,225 [1,676; 5,241]	0,219 [0,089; 0,467]	25,14 [5,347; 188,9]	<0,001	<0,001
	IL-1ra	48	> 166,3	pg/mL	15	57,63	0,31	0,73 [0,61; 0,85]	0,57	0,77	0,80	0,56	0,91	3,85	0,29	-1,355 [-2,203, -0,627]	2,559 [1,148; 4,232]	0,258 [0,110; 0,534]	12,92 [3,152; 68,85]	0,001	0,001
	CD8a+ %	96	< 2,670	%	16	60,15	0,26	0,71 [0,58; 0,83]	0,53	0,75	0,78	0,50	0,91	3,41	0,32	-1,237 [-2,040, -0,536]	2,335 [0,921; 4,002]	0,290 [0,130; 0,585]	10,33 [2,512; 54,70]	0,001	0,002
	Mean Monocyte HLA-DR expression	0	< 15 800	MFI (a.u.)	17	62,18	0,22	0,69 [0,56; 0,83]	0,46	0,69	0,77	0,50	0,88	3,00	0,40	-1,204 [-2,009, -0,500]	2,015 [0,678; 3,513]	0,300 [0,134; 0,606]	7,500 [1,969; 33,55]	0,002	0,005
	% Monocytes	0	< 2,340	%	17	62,18	0,22	0,69 [0,56; 0,82]	0,46	0,69	0,77	0,50	0,88	3,00	0,40	-1,204 [-2,009, -0,500]	2,015 [0,678; 3,513]	0,300 [0,134; 0,606]	7,500 [1,969; 33,55]	0,002	0,005
Catecholamine requirement	IL-6	48	> 19,24	pg/mL	10	47,74	0,27	0,75 [0,58; 0,90]	0,44	0,54	0,90	0,64	0,85	5,40	0,51	-2,169 [-3,377, -1,252]	2,323 [0,871; 3,928]	0,147 [0,050; 0,343]	10,21 [2,389; 50,79]	<0,001	0,002
Lactate increase	pH	48	> 7,459	-	15	47,35	0,42	0,79 [0,62; 0,91]	0,64	0,77	0,87	0,67	0,91	5,92	0,26	-1,856 [-2,930, -1,004]	3,060 [1,570; 4,829]	0,156 [0,053; 0,367]	21,33 [4,807; 125,0]	<0,001	<0,001
	MCP-1	24	< 23,29	pg/mL	15	55,58	0,25	0,71 [0,58; 0,84]	0,49	0,67	0,82	0,53	0,89	3,72	0,40	-1,488 [-2,393, -0,727]	2,181 [0,782; 3,738]	0,226 [0,091; 0,483]	8,857 [2,187; 42,00]	<0,001	0,003
Hypotension / low MAP	P-Selectin+ Monocytes count	0	< 8,97	Events/µL	11	47,74	0,27	0,75 [0,54; 0,87]	0,44	0,54	0,90	0,64	0,85	5,40	0,51	-2,169 [-3,377, -1,252]	2,323 [0,871; 3,928]	0,114 [0,034; 0,286]	10,21 [2,389; 50,79]	<0,001	0,002

TABLE LEGEND. Quartile-Based Directional Associations Between Biomarkers and Clinical Endpoints. Thresholds represent cohort-derived lower- or upper-quartile cutoffs used in the primary directional analysis and should not be interpreted as clinically validated cutoffs. For multivariable two-marker models, both predictors are displayed with their respective regression coefficients, odds ratios, and p values, whereas model-level measures are reported for the combined model. Odds ratios are presented with 95% confidence intervals. AUC values represent apparent within-cohort discrimination. Sensitivity, specificity, positive predictive value (PPV), negative predictive value (NPV), positive and negative likelihood ratios (+LR and −LR), and the Youden index are reported descriptively for the displayed thresholds. All p values are exploratory and unadjusted for multiplicity. AIC, Akaike information criterion; AUC, area under the receiver operating characteristic curve; EPV, events per variable; OR, odds ratio; PPV, positive predictive value; NPV, negative predictive value; LR, likelihood ratio.
