# Supplementary material for: Directional immuno–metabolic signatures following severe trauma are associated with organ-specific clinical endpoints in a prospective cohort
Source: Front Immunol. 2026 Oct 28;17:1964036. doi: 10.3389/fimmu.2026.1964036 (PMC13751341; doi:10.3389/fimmu.2026.1964036)
Supplement: Supplementary file 4 [file DataSheet4.pdf]

Clinical endpoint	Biomarker	Time	transformation	EPV	β(biomarker)	p (Biomarker)	AUC (cont.)	high-odds </>	low-odds </>
Hepatic enzyme elevation	Phagocyte Mean Platelet signal	24	log	33	-1,81 [-3,69; -0,156]	0,041	0,65 [0,47; 0,84]		>
	Monocyte Median HLA-DR expression	96	log	32	-0,983 [-2,16; -0,007]	0,067	0,68 [0,52; 0,84]		>
	IL-1ra	48	log	32	1,08 [0,280; 2,13]	0,020	0,76 [0,62; 0,90]		<
Microbiological culture positivi	CD4+ %	48	log	16	-1,41 [-2,70; -0,304]	0,019	0,71 [0,55; 0,87]	<	
	Monocyte CBC	96	-	15	-0,407 [-0,823; -0,064]	0,032	0,72 [0,53; 0,90]	<	
	Base Excess	0	-	16	-0,291 [-0,570; -0,089]	0,015	0,80 [0,67; 0,93]	<	
	CD4/CD8 ratio	24	-	16	-1,12 [-2,31; -0,189]	0,035	0,71 [0,56; 0,87]	<	
	CD4/CD8 ratio	48	log	16	-2,05 [-3,95; -0,470]	0,019	0,71 [0,55; 0,86]	<	
	IL-8	96	log	16	0,382 [-0,041; 0,852]	0,087	0,63 [0,47; 0,80]	>	
	Monocyte Count	96	log	16	-0,664 [-1,62; 0,219]	0,149	0,59 [0,40; 0,78]	<	
	Monocyte Median HLA-DR expression	96	log	16	-1,10 [-2,25; -0,120]	0,039	0,76 [0,61; 0,91]	<	
	Body temperature	0	-	16	-0,584 [-1,23; -0,022]	0,053	0,65 [0,46; 0,84]	<	
Creatinine-defined renal impair	IL-8	0	log	14	0,525 [0,084; 1,02]	0,025	0,74 [0,58; 0,89]	>	
	CD4/CD8 ratio	0	log	13	-2,95 [-5,37; -1,09]	0,006	0,82 [0,66; 0,97]	<	
	CD4+ %	48	log	13	-1,39 [-2,73; -0,238]	0,026	0,69 [0,49; 0,89]	<	
	Monocyte Median CD64 expression	96	log	13	2,74 [0,393; 5,79]	0,042	0,75 [0,58; 0,92]	>	
	pH	0	-	14	-18,5 [-33,0; -8,19]	0,003	0,82 [0,69; 0,96]	<	
	Lymphocyte CBC	96	-	11	-0,159 [-0,307; -0,038]	0,018	0,76 [0,58; 0,94]	<	
	Neutrophil CBC	96	-	11	0,126 [0,030; 0,252]	0,022	0,78 [0,59; 0,96]	>	
	IL-10	0	log	14	0,664 [0,165; 1,25]	0,014	0,72 [0,55; 0,90]	>	
	Base Excess	0	-	14	-0,332 [-0,636; -0,117]	0,010	0,76 [0,62; 0,91]	<	
	IL-6	0	log	14	0,397 [0,054; 0,812]	0,036	0,75 [0,60; 0,90]	>	
	% Monocytes	0	-	13	-0,501 [-1,07; 0,005]	0,064	0,66 [0,44; 0,88]	<	
	IL-8	48	log	12	0,814 [0,287; 1,48]	0,007	0,70 [0,50; 0,90]	>	
	CD4+ cell count	48	-	13	-0,023 [-0,063; 0,008]	0,193	0,64 [0,45; 0,84]	<	
	IL-1ra	48	log	12	0,893 [0,150; 1,80]	0,029	0,74 [0,58; 0,91]	>	
	IL-8	96	log	12	0,699 [0,218; 1,28]	0,009	0,75 [0,58; 0,93]	>	
	% Granulocytes	96	-	13	0,089 [0,009; 0,200]	0,064	0,70 [0,52; 0,88]	>	
	Monocyte Median HLA-DR expression	96	log	13	-1,24 [-2,48; -0,202]	0,029	0,71 [0,52; 0,90]	<	
	IL-1ra	96	log	12	0,554 [0,055; 1,15]	0,043	0,71 [0,52; 0,90]	>	
	Granulocyte Median CD64 expression	96	log	13	0,869 [0,039; 1,80]	0,048	0,67 [0,49; 0,86]	>	
	MCP-1	96	log	12	0,273 [-0,202; 0,804]	0,279	0,60 [0,36; 0,84]	>	
Berlin-defined ARDS	pH	24	-	28	-22,5 [-43,8; -7,19]	0,014	0,80 [0,65; 0,95]		>
	% Lymphocytes	96	log	28	1,88 [0,394; 3,85]	0,029	0,74 [0,58; 0,90]		<
	pH	0	log	28	-69,3 [-145,9; -9,19]	0,041	0,70 [0,52; 0,88]		>
	CD4/CD8 ratio	48	log	28	-1,22 [-3,19; 0,488]	0,185	0,67 [0,44; 0,89]		>
	Phagocyte Mean Platelet signal	24	log	28	-0,926 [-2,74; 0,821]	0,296	0,55 [0,32; 0,79]		>
	Phagocytic capacity	24	-	28	-0,018 [-0,048; 0,011]	0,226	0,61 [0,39; 0,83]		>
	Neutrophil CBC	48	-	24	-0,002 [-0,093; 0,082]	0,961	0,56 [0,31; 0,81]		>
Tachycardia	IL-10	0	log	18	0,754 [0,263; 1,34]	0,005	0,74 [0,58; 0,90]	>	
	IL-1ra	48	log	15	1,93 [0,863; 3,47]	0,003	0,82 [0,66; 0,97]	>	
	CD8a+ %	96	log	16	-1,71 [-3,27; -0,476]	0,014	0,75 [0,58; 0,92]	<	
	Mean Monocyte HLA-DR expression 1	0	log	17	-1,19 [-2,73; 0,150]	0,099	0,67 [0,49; 0,86]	<	
	% Monocytes	0	-	17	-0,891 [-1,55; -0,350]	0,003	0,77 [0,61; 0,92]	<	
Catecholamine requirement	IL-6	48	log	10	0,648 [0,201; 1,22]	0,011	0,80 [0,62; 0,98]	>	
Lactate increase	pH	48	-	15	32,0 [13,2; 55,8]	0,003	0,82 [0,67; 0,96]	>	
	MCP-1	24	log	15	-0,510 [-1,24; 0,092]	0,122	0,72 [0,54; 0,89]	<	
Hypotension / low MAP	P-Selectin+ Monocytes count	0	log	11	-1,01 [-1,86; -0,311]	0,009	0,76 [0,56; 0,95]	<	

TABLE LEGEND: Continuous-Biomarker Sensitivity Analyses of Principal Directional Associations. Biomarkers retained in the primary quartile-based analysis were re-evaluated as continuous variables, with logarithmic transformation applied where indicated. AUC values represent apparent within-cohort discrimination obtained from the continuous-biomarker analyses and are presented with 95% confidence intervals. These analyses were performed to assess whether the principal endpoint-associated signals identified in the quartile-based analysis were also evident when biomarkers were considered on a continuous scale. AUC, area under the receiver operating characteristic curve; EPV, events per variable.
