# Supplementary material for: Directional immuno–metabolic signatures following severe trauma are associated with organ-specific clinical endpoints in a prospective cohort
Source: Front Immunol. 2026 Oct 28;17:1964036. doi: 10.3389/fimmu.2026.1964036 (PMC13751341; doi:10.3389/fimmu.2026.1964036)
Supplement: Supplementary file 5 [file DataSheet5.pdf]

Clinical endpoint	Biomarker 1	Biomarker 2	Time	Method	EPV	p pred. 1	p pred. 2	OR pred. 1	OR pred. 2	AUC	Predicted probabilities			
Hepatic enzyme elevation	Monocyte Median HLA-DR expression_upper	CD64 Phagocyte count_lower	96	Firth	16	0,006	0,006	0,132 [0,026; 0,563]	0,132 [0,026; 0,563]	0,78 [0,65; 0,91]	none (Intercept)	Median HLA-DR expressi	64 Phagocyte count_l	both
				-	16	0,007	0,007	0,113 [0,020; 0,516]	0,113 [0,020; 0,516]	0,78 [0,65; 0,91]	0,87	0,42	0,42	0,08
Microbiological culture positivity	Monocyte CBC_lower	IL-8_upper	96	Firth	7	0,005	0,043	8,28 [1,84; 47,0]	5,34 [1,06; 32,4]	0,78 [0,64; 0,93]	none (Intercept)	Monocyte CBC_lower	IL-8_upper	both
				-	7	0,007	0,044	10,2 [2,06; 65,4]	6,28 [1,12; 43,2]	0,78 [0,64; 0,93]	0,16	0,65	0,54	0,92
Creatinine-defined renal impairment	IL-8_upper	CD4/CD8 ratio_lower	0	Firth	6	0,001	<0,001	17,4 [3,01; 190]	21,3 [3,63; 240]	0,88 [0,77; 0,99]	none (Intercept)	IL-8_upper	CD4/CD8 ratio_lower	both
				-	6	0,005	0,003	26,7 [3,74; 557]	33,6 [4,61; 716]	0,88 [0,77; 0,99]	0,03	0,46	0,52	0,97
Berlin-defined ARDS	Phagocyte Median CD64 expression_lower	% Lymphocytes_lower	96	Firth	14	0,013	0,006	0,108 [0,014; 0,634]	0,111 [0,016; 0,540]	0,80 [0,65; 0,94]	none (Intercept)	te Median CD64 expressi	% Lymphocytes_lowe	both
				-	14	0,017	0,009	0,083 [0,008; 0,561]	0,087 [0,010; 0,475]	0,80 [0,65; 0,94]	0,92	0,48	0,49	0,07
Tachycardia	Lymphocyte CBC_lower	CD8a cell count_lower	96	Firth	6	0,009	0,023	8,04 [1,65; 49,9]	6,07 [1,27; 35,0]	0,80 [0,65; 0,95]	none (Intercept)	Lymphocyte CBC_lower	CD8a cell count_lowe	both
				-	6	0,012	0,025	10,1 [1,84; 73,1]	7,29 [1,38; 48,1]	0,80 [0,65; 0,95]	0,11	0,56	0,48	0,90

TABLE LEGEND: Supplementary Table S3. Comparison of Standard and Firth-Penalized Logistic Regression for Multivariable Candidate Models. Firth-penalized logistic regression was performed as a sensitivity analysis to assess the influence of limited event numbers and potential sparse-data bias on the retained two-marker models. For each candidate model, estimates from Firth-penalized and standard logistic regression are shown. Odds ratios are presented with 95% confidence intervals. AUC values represent apparent within-cohort discrimination and should not be interpreted as externally validated predictive performance. EPV, events per variable; AUC, area under the receiver operating characteristic curve; OR, odds ratio.
