# Supplementary material for: Directional immuno–metabolic signatures following severe trauma are associated with organ-specific clinical endpoints in a prospective cohort
Source: Front Immunol. 2026 Oct 28;17:1964036. doi: 10.3389/fimmu.2026.1964036 (PMC13751341; doi:10.3389/fimmu.2026.1964036)
Supplement: Supplementary file 6 [file DataSheet6.pdf]

	C	D	E	F	G	H	I	J	K	L
1	**Clinical endpoint**	**Biomarker**	**Timepoint**	**Threshold**	**EPV**	**Biomarker p value**	**ISS p value**	**Biomarker β (95% CI)**	**ISS β (95% CI)**	
2	Hepatic enzyme elevation	Phagocyte Mean Platelet signal	24	> 528	16	0,007	0,175	-1,99 [-3,55; -0,590]	0,065 [-0,025; 0,166]	
3		Monocyte Median HLA-DR expression	96	> 10855	16	0,015	0,433	-1,85 [-3,45; -0,406]	0,039 [-0,055; 0,142]	
4		IL-1ra	48	< 59,06	16	0,023	0,255	-1,60 [-3,06; -0,252]	0,056 [-0,036; 0,159]	
5										
6	Microbiological culture positivity	CD4+ %	48	< 2,79	8	0,006	0,860	2,18 [0,702; 3,86]	0,008 [-0,086; 0,103]	
7		Monocyte CBC	96	< 6,125	8	0,008	0,655	2,15 [0,630; 3,89]	0,021 [-0,072; 0,118]	
8		Base Excess	0	< -2,7	8	0,017	0,557	1,70 [0,333; 3,16]	0,025 [-0,059; 0,113]	
9		CD4/CD8 ratio	24	< 0,95912	8	0,011	0,121	1,88 [0,490; 3,42]	0,069 [-0,015; 0,164]	
10		CD4/CD8 ratio	48	< 1,20141	8	0,013	0,159	1,79 [0,423; 3,30]	0,062 [-0,022; 0,154]	
11		IL-8	96	> 4,79	8	0,087	0,403	1,25 [-0,178; 2,74]	0,036 [-0,048; 0,124]	
12		Monocyte Count	96	< 26,04	7	0,010	0,107	1,99 [0,551; 3,66]	0,080 [-0,010; 0,189]	
13		Monocyte Median HLA-DR expression	96	< 5185,8	8	0,033	0,600	1,58 [0,159; 3,11]	0,024 [-0,067; 0,114]	
14		Body temperature	0	< 35,6	8	0,032	0,542	1,49 [0,143; 2,91]	0,026 [-0,059; 0,109]	
15										
16	Creatinine-defined renal impairment	IL-8	0	> 6,69	7	0,022	0,578	1,90 [0,327; 3,65]	0,029 [-0,075; 0,131]	
17		CD4/CD8 ratio	0	< 0,7684	6	**0,003**	**0,046**	2,52 [0,975; 4,36]	0,108 [0,010; 0,226]	
18		CD4+ %	48	< 2,79	6	0,007	0,627	2,15 [0,642; 3,82]	0,024 [-0,074; 0,125]	
19		Monocyte Median CD64 expression	96	> 19923	6	0,005	0,357	2,14 [0,689; 3,72]	0,046 [-0,050; 0,151]	
20		pH	0	< 7,271	7	0,014	0,171	1,78 [0,387; 3,25]	0,064 [-0,024; 0,162]	
21		Lymphocyte CBC	96	< 11,025	5	0,006	0,594	2,34 [0,737; 4,14]	0,028 [-0,074; 0,135]	
22		Neutrophil CBC	96	> 78,475	5	0,006	0,525	2,33 [0,743; 4,11]	0,034 [-0,072; 0,142]	
23		IL-10	0	> 8,54	7	0,069	0,194	1,34 [-0,111; 2,82]	0,059 [-0,029; 0,153]	
24		Base Excess	0	< -2,7	7	0,044	0,159	1,45 [0,037; 2,91]	0,064 [-0,022; 0,160]	
25		IL-6	0	> 46,7	7	0,074	0,203	1,31 [-0,126; 2,80]	0,060 [-0,031; 0,156]	
26		% Monocytes	0	< 2,34	6	**0,022**	**0,083**	1,72 [0,273; 3,26]	0,081 [-0,007; 0,179]	
27		IL-8	48	> 2,82	6	0,017	0,390	1,81 [0,345; 3,37]	0,041 [-0,051; 0,138]	
28		CD4+ cell count	48	< 22,79	6	0,038	0,383	1,56 [0,096; 3,08]	0,041 [-0,050; 0,138]	
29		IL-1ra	48	> 166,3	6	0,013	0,276	1,87 [0,413; 3,41]	0,051 [-0,038; 0,148]	
30		IL-8	96	> 4,79	6	0,012	0,468	1,99 [0,476; 3,62]	0,035 [-0,059; 0,134]	
31		% Granulocytes	96	> 81,86	6	0,026	0,211	1,63 [0,213; 3,12]	0,056 [-0,032; 0,148]	
32		Monocyte Median HLA-DR expression	96	< 5185,8	6	0,042	0,400	1,54 [0,069; 3,08]	0,040 [-0,055; 0,137]	
33		IL-1ra	96	> 361,21	6	0,043	0,193	1,53 [0,051; 3,05]	0,059 [-0,027; 0,155]	
34		Granulocyte Median CD64 expression	96	> 1590,4	6	0,035	0,325	1,56 [0,119; 3,07]	0,046 [-0,045; 0,143]	
35		**MCP-1**	96	> 37,625	6	0,069	0,325	1,39 [-0,112; 2,93]	0,046 [-0,046; 0,141]	
36										
37	Berlin-defined ARDS	pH	24	> 7,4405	14	0,008	0,988	-2,13 [-3,80; -0,622]	-0,001 [-0,096; 0,103]	
38		% Lymphocytes	96	< 11,64	14	0,028	0,802	-1,67 [-3,25; -0,218]	0,012 [-0,080; 0,113]	
39		pH	0	> 7,368	14	0,021	0,580	-1,88 [-3,60; -0,331]	-0,026 [-0,121; 0,069]	
40		CD4/CD8 ratio	48	> 2,07182	14	0,015	0,753	-2,00 [-3,71; -0,443]	-0,015 [-0,112; 0,085]	
41		Phagocyte Mean Platelet signal	24	> 528	14	0,041	0,628	-1,60 [-3,22; -0,098]	-0,022 [-0,116; 0,070]	
42		Phagocytic capacity	24	> 51,6625	14	0,044	0,819	-1,56 [-3,16; -0,064]	0,010 [-0,078; 0,104]	
43		Neutrophil CBC	48	> 82,175	12	0,192	0,832	-1,04 [-2,66; 0,524]	0,010 [-0,081; 0,106]	
44										
45	Tachycardia	IL-10	0	> 8,54	9	0,001	0,639	3,07 [1,46; 5,14]	0,024 [-0,077; 0,124]	
46		IL-1ra	48	> 166,3	9	0,000	0,268	2,98 [1,45; 4,80]	0,059 [-0,041; 0,171]	
47		CD8a+ %	96	< 2,67	9	0,006	0,579	2,31 [0,763; 4,12]	0,027 [-0,071; 0,123]	
48		Mean Monocyte HLA-DR expression	0	< 15797,575	9	0,008	0,196	1,95 [0,551; 3,49]	0,055 [-0,029; 0,142]	
49		% Monocytes	0	< 2,34	9	0,006	0,128	2,05 [0,649; 3,63]	0,068 [-0,016; 0,162]	
50										
51	Catecholamine requirement	IL-6	48	> 19,24	5	0,005	0,716	2,73 [0,948; 4,86]	-0,021 [-0,142; 0,089]	
52										
53	Lactate increase	pH	48	> 7,4585	7	0,001	0,748	2,73 [1,24; 4,49]	-0,016 [-0,125; 0,079]	
54		MCP-1	24	< 23,285	7	0,004	0,932	2,15 [0,749; 3,71]	0,004 [-0,091; 0,093]	
55										
56	Hypotension / low MAP	P-Selectin+ Monocytes count	0	< 8,97	5	0,003	0,499	2,44 [0,922; 4,17]	-0,034 [-0,140; 0,061]	
57										
58										

TABLE LEGEND: Bivariate sensitivity analyses adjusting biomarker associations for ISS. Each model included the respective dichotomized biomarker and ISS as covariates. Regression coefficients (β) are presented with 95% confidence intervals. EPV denotes events per variable. These analyses were exploratory and were performed to assess whether the direction and statistical evidence of the primary biomarker associations were materially altered after individual adjustment for ISS.

Clinical endpoint	Biomarker	Timepoint	Threshold	EPV	Biomarker p value	Age p value	Biomarker β (95% CI)	Age β (95% CI)
Hepatic enzyme elevation	Phagocyte Mean Platelet signal	24	> 528	16	0,004	0,915	-2,11 [-3,64; -0,743]	-0,002 [-0,042; 0,038]
	Monocyte Median HLA-DR expression	96	> 10855	16	0,004	0,917	-2,06 [-3,58; -0,702]	0,002 [-0,038; 0,043]
	IL-1ra	48	< 59,06	16	0,009	0,871	-1,81 [-3,24; -0,499]	0,003 [-0,036; 0,042]
Microbiological culture positivity	CD4+ %	48	< 2,79	8	0,002	0,459	2,34 [0,925; 3,98]	-0,016 [-0,060; 0,025]
	Monocyte CBC	96	< 6,125	7	0,006	0,860	2,24 [0,728; 3,99]	0,004 [-0,039; 0,048]
	Base Excess	0	< -2,7	8	0,008	0,452	1,91 [0,538; 3,43]	-0,015 [-0,058; 0,024]
	CD4/CD8 ratio	24	< 0,95912	8	0,011	0,385	1,90 [0,500; 3,48]	-0,018 [-0,060; 0,022]
	CD4/CD8 ratio	48	< 1,20141	8	0,014	0,971	1,72 [0,384; 3,16]	-0,001 [-0,040; 0,039]
	IL-8	96	> 4,79	8	0,041	0,982	1,44 [0,081; 2,87]	0,000 [-0,039; 0,039]
	Monocyte Count	96	< 26,04	8	0,014	0,988	1,72 [0,385; 3,16]	0,000 [-0,040; 0,040]
	Monocyte Median HLA-DR expression	96	< 5185,8	8	0,013	0,659	1,76 [0,412; 3,22]	-0,009 [-0,050; 0,030]
	Body temperature	0	< 35,6	8	0,014	0,484	1,69 [0,371; 3,11]	-0,014 [-0,055; 0,024]
Creatinine-defined renal impairment	IL-8	0	> 6,69	7	0,004	0,498	2,10 [0,710; 3,60]	0,014 [-0,027; 0,056]
	CD4/CD8 ratio	0	< 0,7684	6	0,002	0,612	2,29 [0,871; 3,85]	0,011 [-0,033; 0,057]
	CD4+ %	48	< 2,79	6	0,002	0,797	2,27 [0,851; 3,84]	0,006 [-0,038; 0,050]
	Monocyte Median CD64 expression	96	> 19923	6	0,002	0,672	2,27 [0,858; 3,83]	0,009 [-0,034; 0,054]
	pH	0	< 7,271	7	0,004	0,131	2,17 [0,743; 3,79]	0,034 [-0,008; 0,081]
	Lymphocyte CBC	96	< 11,025	5	0,004	0,450	2,80 [1,05; 5,02]	-0,021 [-0,082; 0,031]
	Neutrophil CBC	96	> 78,475	5	0,004	0,566	2,67 [0,990; 4,72]	-0,015 [-0,072; 0,035]
	IL-10	0	> 8,54	7	0,030	0,639	1,56 [0,170; 3,02]	0,010 [-0,032; 0,052]
	Base Excess	0	< -2,7	7	0,026	0,396	1,57 [0,199; 3,00]	0,017 [-0,023; 0,059]
	IL-6	0	> 46,7	7	0,019	0,318	1,65 [0,289; 3,10]	0,020 [-0,019; 0,061]
	% Monocytes	0	< 2,34	6	0,013	0,632	1,77 [0,392; 3,24]	0,010 [-0,030; 0,052]
	IL-8	48	> 2,82	6	0,008	0,585	2,14 [0,624; 3,87]	-0,013 [-0,064; 0,033]
	CD4+ cell count	48	< 22,79	6	0,017	0,965	1,76 [0,336; 3,27]	0,001 [-0,041; 0,042]
	IL-1ra	48	> 166,3	6	0,007	0,599	2,01 [0,590; 3,55]	0,012 [-0,033; 0,058]
	IL-8	96	> 4,79	6	0,004	0,588	2,20 [0,743; 3,81]	0,012 [-0,033; 0,059]
	% Granulocytes	96	> 81,86	6	0,019	0,708	1,90 [0,373; 3,61]	-0,009 [-0,057; 0,036]
	Monocyte Median HLA-DR expression	96	< 5185,8	6	0,015	0,607	1,74 [0,363; 3,21]	0,011 [-0,031; 0,053]
	IL-1ra	96	> 361,21	6	0,026	0,589	1,65 [0,217; 3,17]	0,012 [-0,031; 0,056]
	Granulocyte Median CD64 expression	96	> 1590,4	6	0,014	0,537	1,76 [0,379; 3,23]	0,013 [-0,029; 0,057]
	MCP-1	96	> 37,625	6	0,027	0,657	1,63 [0,200; 3,13]	0,009 [-0,032; 0,051]
Berlin-defined ARDS	pH	24	> 7,4405	14	0,008	0,521	-2,13 [-3,81; -0,611]	0,015 [-0,031; 0,065]
	% Lymphocytes	96	< 11,64	14	0,010	**0,057**	-2,71 [-5,12; -0,856]	0,055 [0,003; 0,118]
	pH	0	> 7,368	14	0,015	0,186	-2,20 [-4,19; -0,532]	0,035 [-0,013; 0,094]
	CD4/CD8 ratio	48	> 2,07182	14	0,015	0,588	-1,98 [-3,68; -0,422]	0,013 [-0,033; 0,060]
	Phagocyte Mean Platelet signal	24	> 528	14	0,056	0,722	-1,47 [-3,05; 0,022]	0,008 [-0,037; 0,054]
	Phagocytic capacity	24	> 51,6625	14	0,021	0,127	-2,15 [-4,20; -0,445]	0,041 [-0,008; 0,100]
	Neutrophil CBC	48	> 82,175	12	0,170	0,566	-1,10 [-2,73; 0,463]	0,013 [-0,031; 0,059]
Tachycardia	IL-10	0	> 8,54	9	0,001	0,126	3,04 [1,45; 5,08]	0,036 [-0,009; 0,085]
	IL-1ra	48	> 166,3	7	<0,001	**0,023**	3,83 [1,98; 6,37]	0,069 [0,015; 0,137]
	CD8a+ %	96	< 2,67	8	0,008	0,774	2,37 [0,737; 4,28]	0,007 [-0,043; 0,056]
	Mean Monocyte HLA-DR expression	0	< 15797,575	8	0,003	**0,022**	2,50 [0,990; 4,32]	0,053 [0,011; 0,103]
	% Monocytes	0	< 2,34	8	0,005	**0,057**	2,23 [0,767; 3,93]	0,044 [0,002; 0,095]
Catecholamine requirement	IL-6	48	> 19,24	5	0,004	0,628	2,43 [0,853; 4,26]	0,014 [-0,044; 0,071]
Lactate increase	pH	48	> 7,4585	7	0,001	0,729	2,79 [1,25; 4,65]	-0,008 [-0,057; 0,038]
	MCP-1	24	< 23,285	7	0,004	0,568	2,14 [0,734; 3,71]	0,012 [-0,029; 0,055]
Hypotension / low MAP	P-Selectin+ Monocytes count	0	< 8,97	5	0,004	0,827	2,35 [0,816; 4,09]	-0,005 [-0,051; 0,040]

TABLE LEGEND: Bivariate Sensitivity Analyses Adjusting Biomarker Associations for age. Each model included the respective dichotomized biomarker and age as covariates. Regression coefficients (β) are presented with 95% confidence intervals. EPV denotes events per variable. These exploratory analyses were performed to assess whether the direction and statistical evidence of the primary biomarker associations were materially altered after individual adjustment for age.

Clinical endpoint	Biomarker	Timepoint	Threshold	EPV	Gender p value	iomarker p valu	Gender β (95% CI)	Biomarker β (95% CI)
Hepatic enzyme elevation	Phagocyte Mean Platelet signal	24	> 528	16	0,044	0,003	-1,57 [-3,20; -0,087]	-2,39 [-4,08; -0,928]
	Monocyte Median HLA-DR expression	96	> 10855	16	0,188	0,007	-0,973 [-2,46; 0,487]	-2,00 [-3,54; -0,604]
	IL-1ra	48	< 59,06	16	0,057	0,009	-1,45 [-3,03; 0,016]	-1,89 [-3,41; -0,516]
Microbiological culture positivity	CD4+ %	48	< 2,79	8	0,445	0,002	0,588 [-0,954; 2,13]	2,29 [0,896; 3,88]
	Monocyte CBC	96	< 6,125	7	0,408	0,006	0,670 [-0,946; 2,29]	2,25 [0,741; 4,00]
	Base Excess	0	< -2,7	8	0,632	0,011	0,350 [-1,14; 1,78]	1,78 [0,443; 3,21]
	CD4/CD8 ratio	24	< 0,95912	8	0,690	0,014	0,290 [-1,19; 1,71]	1,71 [0,376; 3,15]
	CD4/CD8 ratio	48	< 1,20141	8	0,489	0,012	0,507 [-0,964; 1,96]	1,77 [0,420; 3,23]
	IL-8	96	> 4,79	8	0,460	0,040	0,536 [-0,924; 1,97]	1,45 [0,090; 2,90]
	Monocyte Count	96	< 26,04	8	0,851	0,016	-0,144 [-1,76; 1,31]	1,76 [0,373; 3,29]
	Monocyte Median HLA-DR expression	96	< 5185,8	8	0,914	0,015	0,080 [-1,45; 1,50]	1,71 [0,360; 3,17]
	Body temperature	0	< 35,6	8	0,935	0,019	-0,060 [-1,58; 1,33]	1,62 [0,295; 3,04]
Creatinine-defined renal impairment	IL-8	0	> 6,69	7	0,420	0,003	-0,682 [-2,51; 0,882]	2,21 [0,815; 3,77]
	CD4/CD8 ratio	0	< 0,7684	6	0,493	0,002	-0,591 [-2,45; 1,02]	2,34 [0,915; 3,94]
	CD4+ %	48	< 2,79	6	0,307	0,002	-0,972 [-3,12; 0,755]	2,33 [0,889; 3,93]
	Monocyte Median CD64 expression	96	> 19923	6	0,169	0,002	-1,37 [-3,62; 0,407]	2,52 [1,02; 4,27]
	pH	0	< 7,271	7	0,952	0,007	0,050 [-1,68; 1,64]	1,94 [0,578; 3,41]
	Lymphocyte CBC	96	< 11,025	5	0,197	0,003	-1,43 [-3,93; 0,536]	2,83 [1,14; 4,96]
	Neutrophil CBC	96	> 78,475	5	0,391	0,003	-0,879 [-3,16; 0,988]	2,55 [0,969; 4,38]
	IL-10	0	> 8,54	7	0,460	0,017	-0,591 [-2,33; 0,893]	1,69 [0,328; 3,14]
	Base Excess	0	< -2,7	7	0,538	0,017	-0,498 [-2,25; 1,00]	1,69 [0,329; 3,14]
	IL-6	0	> 46,7	7	0,460	0,017	-0,591 [-2,33; 0,893]	1,69 [0,328; 3,14]
	% Monocytes	0	< 2,34	6	0,380	0,010	-0,740 [-2,57; 0,809]	1,94 [0,515; 3,50]
	IL-8	48	> 2,82	6	0,080	0,005	-2,16 [-5,28; -0,097]	2,36 [0,817; 4,15]
	CD4+ cell count	48	< 22,79	6	0,316	0,015	-0,906 [-2,97; 0,728]	1,77 [0,378; 3,27]
	IL-1ra	48	> 166,3	6	0,080	0,005	-2,16 [-5,28; -0,097]	2,36 [0,817; 4,15]
	IL-8	96	> 4,79	6	0,110	0,004	-1,95 [-5,07; 0,093]	2,38 [0,834; 4,16]
	% Granulocytes	96	> 81,86	6	0,316	0,015	-0,906 [-2,97; 0,728]	1,77 [0,378; 3,27]
	Monocyte Median HLA-DR expression	96	< 5185,8	6	0,125	0,008	-1,51 [-3,74; 0,229]	2,13 [0,633; 3,86]
	IL-1ra	96	> 361,21	6	0,082	0,016	-2,10 [-5,21; -0,093]	2,00 [0,442; 3,76]
	Granulocyte Median CD64 expression	96	> 1590,4	6	0,206	0,011	-1,18 [-3,30; 0,487]	1,91 [0,484; 3,48]
	MCP-1	96	> 37,625	6	0,130	0,025	-1,76 [-4,80; 0,169]	1,74 [0,251; 3,35]
Berlin-defined ARDS	pH	24	> 7,4405	14	0,468	0,010	0,693 [-1,08; 2,83]	-2,08 [-3,76; -0,560]
	% Lymphocytes	96	< 11,64	14	0,522	0,040	0,591 [-1,13; 2,66]	-1,55 [-3,10; -0,102]
	pH	0	> 7,368	14	0,148	0,016	1,52 [-0,322; 3,93]	-2,21 [-4,27; -0,534]
	CD4/CD8 ratio	48	> 2,07182	14	0,508	0,018	0,628 [-1,13; 2,75]	-1,94 [-3,64; -0,377]
	Phagocyte Mean Platelet signal	24	> 528	14	0,430	0,056	0,719 [-0,959; 2,78]	-1,47 [-3,03; 0,021]
	Phagocytic capacity	24	> 51,6625	14	0,067	0,020	2,38 [0,210; 5,62]	-2,71 [-5,74; -0,742]
	Neutrophil CBC	48	> 82,175	12	0,205	0,190	1,15 [-0,494; 3,21]	-1,05 [-2,68; 0,516]
Tachycardia	IL-10	0	> 8,54	9	0,525	<0,001	0,511 [-1,12; 2,10]	3,20 [1,65; 5,23]
	IL-1ra	48	> 166,3	7	0,866	<0,001	-0,154 [-2,09; 1,58]	3,04 [1,54; 4,84]
	CD8a+ %	96	< 2,67	8	0,778	0,002	-0,233 [-2,00; 1,33]	2,53 [1,04; 4,32]
	Mean Monocyte HLA-DR expression	0	< 15797,575	8	0,134	0,002	1,15 [-0,330; 2,74]	2,41 [0,957; 4,10]
	% Monocytes	0	< 2,34	8	0,660	0,004	0,321 [-1,17; 1,75]	2,06 [0,698; 3,59]
Catecholamine requirement	IL-6	48	> 19,24	5	0,727	0,002	-0,347 [-2,54; 1,52]	2,55 [1,02; 4,32]
Lactate increase	pH	48	> 7,4585	7	0,523	0,001	-0,583 [-2,56; 1,11]	2,74 [1,25; 4,52]
	MCP-1	24	< 23,285	7	0,854	0,004	-0,153 [-1,92; 1,43]	2,13 [0,728; 3,70]
Hypotension / low MAP	P-Selectin+ Monocytes count	0	< 8,97	5	0,762	0,003	0,250 [-1,46; 1,85]	2,26 [0,796; 3,88]

TABLE LEGEND: Bivariate sensitivity analyses adjusting biomarker associations for gender. Each model included the respective dichotomized biomarker and gender. Regression coefficients (β) are presented with 95% confidence intervals. EPV denotes events per variable. These analyses were exploratory and were performed to assess whether the direction and statistical evidence of the primary biomarker associations were materially altered after individual adjustment for gender.
