# Supplementary material for: Directional immuno–metabolic signatures following severe trauma are associated with organ-specific clinical endpoints in a prospective cohort
Source: Front Immunol. 2026 Oct 28;17:1964036. doi: 10.3389/fimmu.2026.1964036 (PMC13751341; doi:10.3389/fimmu.2026.1964036)
Supplement: Supplementary file 7 [file DataSheet7.pdf]

Event	Parameter	Time	n	</> threshold	EPV	AUC	Sensitivity	Specifity	OR predictor	p pred.
Hepatic enzyme elevation	Phagocyte Mean Platelet signal	24	18	> 528	1					
Creatinine-defined renal impairment	Base Excess	0	41	< -2,70	5	0,72 [0,47; 0,96]	0,33	0,94	7,50 [1,04; 67,4]	0,047
	pH	0	41	< 7,271	5	0,62 [0,37; 0,86]	0,25	0,91	3,33 [0,379; 24,8]	0,236
	IL-6	0	41	> 46,70	5	0,82 [0,61; 1,00]	0,40	0,97	20,0 [2,45; 431]	0,013
	IL-8	0	41	> 6,690	5	0,83 [0,63; 1,00]	0,44	0,97	24,8 [2,96; 544]	0,008
	IL-10	0	41	> 8,540	5	0,72 [0,47; 0,96]	0,33	0,94	7,50 [1,04; 67,4]	0,047
	% Monocytes	0	41	< 2,340	5	0,62 [0,37; 0,86]	0,25	0,91	3,33 [0,379; 24,8]	0,236
	CD4/CD8 ratio	0	40	< 0,768	5	0,63 [0,38; 0,88]	0,29	0,91	4,00 [0,445; 31,0]	0,180
Berlin-defined ARDS	pH	0	35	> 7,368	12	0,61 [0,46; 0,76]	0,42	0,82	0,311 [0,041; 1,55]	0,187
	pH	24	32	> 7,441	9	0,64 [0,49; 0,79]	0,36	0,90	0,194 [0,010; 1,33]	0,152
	Phagocytic capacity	24	31	> 51,66	9	0,63 [0,48; 0,78]	0,36	0,89	0,219 [0,011; 1,53]	0,186
	Phagocyte Mean Platelet signal	24	31	> 528,0	9	0,57 [0,39; 0,75]	0,33	0,80	0,500 [0,064; 2,71]	0,449
Tachycardia	IL-10	0	36	> 8,540	3					
	% Monocytes	0	36	< 2,340	3					
	Mean Monocyte HLA-DR expression 1	0	36	< 15 800	3					
Lactate increase	MCP-1	24	38	< 23,29	3					
Hypotension / low MAP	P-Selectin+ Monocytes count	0	48	< 8,97	8	0,74 [0,55; 0,93]	0,45	0,92	9,44 [1,85; 57,4]	0,009

TABLE LEGEND: Exploratory First-Occurrence Sensitivity Analyses Restricted to Biomarker Measurements Preceding the Respective Clinical Endpoint. Patients were excluded from the respective analysis at and after the first recorded occurrence of the endpoint, such that only biomarker measurements preceding a subsequent first occurrence were retained. Quartile thresholds from the primary analysis were carried forward unchanged and were not re-optimized. AUC, sensitivity, and specificity are reported descriptively for these predefined thresholds. Because incident event counts declined markedly after exclusion of prevalent cases, these analyses were exploratory, and models with insufficient incident events were not estimated. Microbiological culture positivity was not subjected to formal first-occurrence analysis because the available study timestamp did not reliably identify the exact time of first culture positivity. EPV, events per variable; AUC, area under the receiver operating characteristic curve; ER, emergency room admission.
