# Supplementary material for: Directional immuno–metabolic signatures following severe trauma are associated with organ-specific clinical endpoints in a prospective cohort
Source: Front Immunol. 2026 Oct 28;17:1964036. doi: 10.3389/fimmu.2026.1964036 (PMC13751341; doi:10.3389/fimmu.2026.1964036)
Supplement: Supplementary file 8 [file DataSheet8.pdf]

Event	Parameter	Time	Inclusion Prob.	Apparent AUC	Mean Optimism	Corrected AUC	Coefficient	original β	BCa CI lower	BCa CI upper	BCa annot.	bootstrap bias (boot-original)	standardized bootstrap bias (bias/SD)	std. bootstr. bias annot.	sign stability	sign stability annot.
Hepatic enzyme elevation	Monocyte Median HLA-DR expression_u	96	0,16	0,78	0,08	**0,70**	(Intercept)	1,859	0,776	3,292		0,447	0,170		1,00	
							Monocyte Mec > 10 855	-2,184	-4,036	-0,399		-0,791	-0,220		1,00	
							CD64+ Phago < 24,08	-2,184	-4,201	-0,278		-0,829	-0,230		1,00	
	**Phagocyte Me**	24	**0,65**	0,70	0,03	**0,67**	(Intercept)	1,288	0,406	2,104		0,056	0,130		1,00	
							Phagocyte Me > 528	-2,099	-3,664	-0,490		-0,439	-0,170		1,00	
	Monocyte Me	96	0,46	0,67	0,06	0,61	(Intercept)	1,135	0,319	2,015		0,065	0,160		1,00	
							Monocyte Mec > 10 855	-1,828	-3,496	-0,162		-0,372	-0,160		0,99	
	IL-1ra	48	0,24	0,63	0,09	0,54	(Intercept)	0,993	0,232	1,825		0,018	0,050		1,00	
							IL-1ra < 59,06	-1,330	-2,914	0,210	**includes 0**	-0,073	-0,070		0,97	
Microbiological culture positivity	C_I~Monocyte CBC_lower + IL-8_upper	96	0,17	0,78	0,09	**0,69**	(Intercept)	-1,681	-3,120	-0,605		-0,347	-0,160		1,00	
							Monocyte CB(< 6,125	2,318	0,091	4,369		1,312	0,280		1,00	
							IL-8_upper > 4,790	1,837	-0,360	5,105	**includes 0**	0,977	0,220		0,98	
	CD4+ %	48	**0,55**	0,69	0,05	0,64	(Intercept)	-1,288	-2,169	-0,530		-0,039	-0,090		1,00	
							CD4+ % < 2,790	1,981	0,292	3,584		0,361	0,160		1,00	
	**Monocyte CB**	96	**0,62**	0,71	0,04	**0,67**	(Intercept)	-1,232	-2,163	-0,435		-0,070	-0,150		1,00	
							Monocyte CB(< 6,125	2,213	-0,099	3,999	**includes 0**	1,054	0,260		1,00	
	Base Excess	0	0,50	0,68	0,05	0,63	(Intercept)	-1,322	-2,212	-0,554		-0,046	-0,100		1,00	
							Base Excess < -2,70	1,792	0,315	3,471		0,224	0,120		0,99	
	CD4/CD8 rat	24	**0,59**	0,69	0,05	0,64	(Intercept)	-1,288	-2,140	-0,499		-0,061	-0,130		1,00	
							CD4/CD8 ratio < 0,959	1,981	0,044	3,719		0,567	0,190		0,99	
	CD4/CD8 rat	48	0,33	0,64	0,07	0,57	(Intercept)	-1,135	-1,946	-0,452		-0,025	-0,060		1,00	
							CD4/CD8 ratio < 1,201	1,471	-0,019	2,790	**includes 0**	0,163	0,110		0,99	
	IL-8	96	0,31	0,64	0,08	0,56	(Intercept)	-1,099	-1,856	-0,279		-0,043	-0,110		1,00	
							IL-8 > 4,790	1,435	-0,278	2,893	**includes 0**	0,157	0,080		0,97	
	Monocyte Co	96	0,35	0,64	0,08	0,56	(Intercept)	-1,135	-2,038	-0,395		-0,043	-0,100		1,00	
							Monocyte Cou < 26,04	1,471	-0,145	3,126	**includes 0**	0,124	0,080		0,97	
	Monocyte Me	96	0,34	0,64	0,07	0,57	(Intercept)	-1,135	-1,856	-0,316		-0,032	-0,080		1,00	
							Monocyte Mec < 5 186	1,471	-0,088	2,970	**includes 0**	0,139	0,090		0,98	
	Body temper	0	0,34	0,65	0,08	0,57	(Intercept)	-1,237	-2,197	-0,496		-0,060	-0,140		1,00	
							Body temperat < 35,60	1,573	0,077	3,271		0,068	0,040		0,99	
Creatinine-defined renal impairment	IL-8_upper + CD4/CD8 ratio_lower	0	0,54	0,88	0,03	**0,85**	(Intercept)	-3,446	-20,780	-1,785		-5,997	-0,680	**0,5 - 1,0**	1,00	
							IL-8 > 6,690	3,284	0,402	20,728		5,892	0,690	**0,5 - 1,0**	1,00	
							CD4/CD8 ratio < 0,768	3,514	1,104	20,982		6,131	0,690	**0,5 - 1,0**	1,00	
	**IL-8**	0	**0,69**	0,72	0,03	**0,68**	(Intercept)	-1,674	-2,836	-0,798		-0,144	-0,110		1,00	
							IL-8 > 6,690	2,144	0,377	3,853		0,269	0,130		1,00	
	**CD4/CD8 rati**	0	**0,84**	0,75	0,02	**0,73**	(Intercept)	-1,856	-2,901	-0,932		-0,158	-0,110		1,00	
							CD4/CD8 ratio < 0,768	2,549	0,799	4,480		0,416	0,170		1,00	
	**CD4+ %**	48	**0,62**	0,70	0,04	**0,66**	(Intercept)	-1,642	-2,773	-0,771		-0,092	-0,120		1,00	
							CD4+ % < 2,790	1,979	0,406	3,644		0,171	0,100		0,99	
	**Monocyte Me**	96	**0,83**	0,75	0,02	**0,73**	(Intercept)	-1,856	-2,970	-0,913		-0,267	-0,150		1,00	
							Monocyte Mec > 19 923	2,549	0,880	4,508		0,509	0,180		1,00	
	**pH**	0	**0,77**	0,73	0,03	**0,70**	(Intercept)	-1,705	-2,565	-0,817		-0,099	-0,130		1,00	
							pH < 7,271	2,398	0,737	4,094		0,405	0,160		1,00	
	**Lymphocyte**	96	**0,80**	0,75	0,03	**0,73**	(Intercept)	-1,910	-3,386	-0,848		-0,383	-0,160		1,00	
							Lymphocyte C < 11,03	2,469	0,623	4,443		0,702	0,200		1,00	
	**Neutrophil C**	96	**0,81**	0,75	0,02	**0,73**	(Intercept)	-1,910	-3,258	-0,847		-0,415	-0,180		1,00	
							Neutrophil CB > 78,48	2,469	0,271	4,249		0,732	0,220		1,00	
	IL-10	0	**0,51**	0,67	0,06	0,61	(Intercept)	-1,488	-2,391	-0,732		-0,056	-0,130		1,00	
							IL-10 > 8,540	1,642	-0,003	3,113	**includes 0**	0,082	0,080		0,99	
	Base Excess	0	0,49	0,67	0,06	0,61	(Intercept)	-1,488	-2,512	-0,693		-0,088	-0,120		1,00	
							Base Excess < -2,70	1,642	0,101	3,177		0,104	0,080		0,99	
	IL-6	0	0,48	0,67	0,05	0,62	(Intercept)	-1,488	-2,398	-0,653		-0,062	-0,140		1,00	
							IL-6 > 46,70	1,642	-0,148	3,185	**includes 0**	0,108	0,080		0,98	
	% Monocytes	0	**0,61**	0,69	0,05	0,64	(Intercept)	-1,642	-2,708	-0,847		-0,157	-0,120		1,00	
							% Monocytes < 2,340	1,796	0,153	3,352		0,166	0,090		0,99	
	**IL-8**	48	**0,71**	0,72	0,04	**0,69**	(Intercept)	-1,856	-2,862	-0,934		-0,184	-0,140		1,00	
							IL-8 > 2,820	2,193	0,508	3,753		0,328	0,180		1,00	
	CD4+ cell co	48	0,37	0,65	0,07	0,57	(Intercept)	-1,455	-2,367	-0,568		-0,040	-0,090		1,00	
							CD4+ cell cou < 22,79	1,455	-0,182	3,121	**includes 0**	-0,043	-0,020		0,96	
	**IL-1ra**	48	**0,75**	0,72	0,03	**0,69**	(Intercept)	-1,856	-2,970	-0,955		-0,187	-0,140		1,00	
							IL-1ra > 166,3	2,193	0,509	3,952		0,268	0,140		1,00	
	**IL-8**	96	**0,74**	0,72	0,04	**0,69**	(Intercept)	-1,825	-2,890	-0,892		-0,180	-0,150		1,00	
							IL-8 > 4,790	2,161	0,415	3,845		0,244	0,170		1,00	
	**% Granulocyt**	96	**0,61**	0,70	0,04	**0,66**	(Intercept)	-1,642	-2,743	-0,749		-0,116	-0,150		1,00	
							% Granulocyte > 81,86	1,979	0,412	3,644		0,154	0,130		1,00	
	Monocyte Me	96	0,36	0,65	0,08	0,57	(Intercept)	-1,455	-2,485	-0,653		-0,073	-0,090		1,00	
							Monocyte Mec < 5 186	1,455	-0,339	3,016	**includes 0**	0,072	0,060		0,97	
	IL-1ra	96	**0,75**	0,67	0,07	0,60	(Intercept)	-1,609	-2,485	-0,773		-0,049	-0,100		1,00	
							IL-1ra > 361,2	1,609	0,262	3,409		0,057	0,060		0,99	
	Granulocyte	96	0,36	0,65	0,07	0,57	(Intercept)	-1,455	-2,398	-0,651		-0,112	-0,110		1,00	
							Granulocyte M > 1 590	1,455	-0,322	3,206	**includes 0**	0,094	0,050		0,97	
	MCP-1	96	0,46	0,67	0,07	0,60	(Intercept)	-1,609	-2,719	-0,735		-0,092	-0,190		1,00	
							MCP-1 > 37,63	1,609	-0,030	3,060	**includes 0**	0,079	0,070		0,99	
Berlin-defined ARDS	Phagocyte Median CD64 expression_lower + %	96	0,16	0,80	0,09	**0,71**	(Intercept)	2,386	0,841	20,498		2,332	0,380		1,00	
							Phagocyte Me < 13 596	-2,483	-20,687	0,299	**includes 0**	-3,170	-0,450		0,99	
							% Lymphocyte < 11,64	-2,444	-19,671	0,300	**includes 0**	-2,407	-0,390		1,00	
	**pH**	24	**0,72**	0,72	0,04	**0,68**	(Intercept)	1,569	0,560	2,674		0,140	0,120		1,00	
							pH > 7,441	-2,128	-3,941	-0,373		-0,404	-0,170		1,00	
	**% Lymphocyt**	96	**0,66**	0,70	0,05	**0,66**	(Intercept)	1,526	0,547	2,565		0,183	0,130		1,00	
							% Lymphocyte < 11,64	-1,863	-3,401	-0,008		-0,328	-0,170		0,99	
	pH	0	**0,53**	0,68	0,06	0,62	(Intercept)	1,386	0,511	2,303		0,101	0,100		1,00	
							pH > 7,368	-1,792	-3,486	-0,128		-0,399	-0,160		0,99	
	**CD4/CD8 rati**	48	**0,63**	0,72	0,05	**0,67**	(Intercept)	1,609	0,742	2,730		0,195	0,120		1,00	
							CD4/CD8 ratio > 2,072	-2,303	-4,357	-0,133		-0,901	-0,230		1,00	
	Phagocyte M	24	0,41	0,66	0,07	0,59	(Intercept)	1,344	0,348	2,455		0,064	0,120		1,00	
							Phagocyte Me > 528,0	-1,526	-3,206	0,478	**includes 0**	-0,148	-0,080		0,96	
	Phagocytic c	24	0,45	0,66	0,07	0,59	(Intercept)	1,344	0,435	2,485		0,098	0,180		1,00	
							Phagocytic cap > 51,66	-1,526	-3,217	0,157	**includes 0**	-0,318	-0,140		0,97	
	Neutrophil C	48	0,22	0,60	0,12	0,48	(Intercept)	0,999	0,080	2,035		0,060	0,120		0,99	
							Neutrophil CB > 82,18	-0,999	-2,887	0,750	**includes 0**	-0,026	-0,020		0,89	**≤0,95**
Tachycardia	Lymphocyte CBC_lower + CD8a+ cell count_lower	96	0,30	0,80	0,06	**0,74**	(Intercept)	-2,053	-3,599	-0,826		-0,882	-0,210		1,00	
							Lymphocyte C < 11,03	2,310	0,167	20,153		1,609	0,300		1,00	
							CD8a+ cell co < 19,17	1,986	-0,354	4,483	**includes 0**	1,217	0,250		0,98	
	**IL-10**	0	**0,88**	0,78	0,02	**0,76**	(Intercept)	-1,488	-2,303	-0,595		-0,066	-0,140		1,00	
							IL-10 > 8,540	3,193	1,025	19,952		2,742	0,430		1,00	
	**IL-1ra**	48	**0,92**	0,80	0,01	**0,79**	(Intercept)	-1,856	-2,970	-0,916		-0,208	-0,120		1,00	
							IL-1ra > 166,3	3,466	1,132	21,202		2,730	0,420		1,00	
	**CD8a+ %**	96	**0,70**	0,73	0,03	**0,70**	(Intercept)	-1,386	-2,335	-0,551		-0,073	-0,150		1,00	
							CD8a+ % < 2,670	2,485	0,701	4,326		0,958	0,240		1,00	
	**Mean Monocy**	0	**0,64**	0,70	0,04	**0,66**	(Intercept)	-1,288	-2,169	-0,537		-0,063	-0,150		1,00	
							Mean Monocy < 15 800	2,099	0,639	3,738		0,360	0,160		1,00	
	**% Monocytes**	0	**0,61**	0,70	0,04	**0,66**	(Intercept)	-1,288	-2,197	-0,509		-0,064	-0,140		1,00	
							% Monocytes < 2,340	2,099	0,378	3,507		0,417	0,190		1,00	
Catecholamine requirement	**IL-6**	48	**0,74**	0,72	0,04	**0,69**	(Intercept)	-2,110	-3,526	-1,036		-0,363	-0,160		1,00	
							IL-6 > 19,24	2,110	0,143	3,907		0,328	0,130		0,99	
Lactate increase relative to admission	**pH**	48	**0,82**	0,75	0,02	**0,73**	(Intercept)	-1,609	-2,803	-0,780		-0,104	-0,110		1,00	
							pH > 7,459	2,708	0,846	4,477		0,966	0,250		1,00	
	**MCP-1**	24	**0,70**	0,71	0,04	**0,67**	(Intercept)	-1,455	-2,512	-0,613		-0,078	-0,100		1,00	
							MCP-1 < 23,29	2,148	0,525	3,814		0,299	0,150		1,00	
Hypotension / low MAP	**P-Selectin+ M**	0	**0,81**	0,74	0,03	**0,71**	(Intercept)	-2,140	-3,450	-1,135		-0,360	-0,170		1,00	
							P-Selectin+ M < 8,97	2,294	0,409	3,925		0,369	0,160		1,00	

TABLE LEGEND: Bootstrap Reproducibility, Optimism, and Coefficient Stability of Candidate Biomarker Models. The complete model-selection procedure was repeated in 500 bootstrap samples. Inclusion probability represents the proportion of bootstrap iterations in which the respective candidate biomarker or multivariable model was retained by the predefined selection procedure. Apparent AUC refers to discrimination in the original analysis cohort. Mean optimism was estimated across bootstrap iterations, and the optimism-corrected AUC was calculated by subtracting mean optimism from the original apparent AUC. Coefficient stability was assessed separately by non-parametric bootstrap resampling with 1,000 iterations. Original β denotes the regression coefficient estimated in the original analysis cohort. BCa 95% confidence intervals were derived from the bootstrap distribution of the coefficient. Bootstrap bias was calculated as the difference between the bootstrap mean coefficient and the original coefficient, and standardized bootstrap bias as this difference divided by the bootstrap standard deviation. Sign stability denotes the proportion of bootstrap coefficient estimates retaining the same direction as the original coefficient. BCa confidence intervals including zero, standardized bootstrap bias >0.5 SD, and sign stability <0.95 were predefined as pragmatic indicators of potentially reduced coefficient stability rather than formal statistical decision criteria.
Bootstrap inclusion probability and optimism-corrected AUC quantify different aspects of model reproducibility and should not be interpreted interchangeably. Likewise, optimism-corrected AUCs represent internal bootstrap estimates and do not constitute external validation or calibrated clinical predictive performance. AUC, area under the receiver operating characteristic curve; BCa, bias-corrected and accelerated; β, regression coefficient; SD, standard deviation.
